# The use of carinated items in the Levantine Aurignacian—Insights from layer D, Hayonim Cave, W. Galilee, Israel

**DOI:** 10.1371/journal.pone.0301102

**Published:** 2024-07-24

**Authors:** Hannah Parow-Souchon, Anna Belfer-Cohen

**Affiliations:** 1 Austrian Archaeological Institute, Austrian Academy of Sciences, Vienna, Austria; 2 William F. Albright Institute of Archaeological Research, Jerusalem, Israel; 3 Department of Bible Studies, Archaeology and the Ancient Near East, Ben-Gurion University of the Negev, Beer Sheva, Israel; 4 Institute of Archaeology, The Hebrew University of Jerusalem, Jerusalem, Israel; Sapienza University of Rome: Universita degli Studi di Roma La Sapienza, ITALY

## Abstract

A longstanding debate concerns the function of carinated elements in both, the Levantine, and European Aurignacian. The present study aims to contribute to this topic with the evaluation of the carinated assemblage from layer D in Hayonim Cave, Western Galilee, Israel, one of the type sites of the Levantine Aurignacian. An operational chain reconstruction with an attribute analysis is paired with a typological approach to the preparation and maintenance products based on artefacts defined as West European Aurignacian. The results of this study are investigated with multivariate statistics offering a methodological contribution. The data is subjected to a transformation into a distance matrix using the Gower distance and tested with the *adonis*-algorithm for significance. The results clearly indicate that the carinated items in Hayonim Cave did fully or partially function as cores. They are accompanied by diagnostic preparation- and- maintenance products known from the literature e.g. Thèmes bladelets. The statistical analysis indicates only a minor correlation with stratigraphy yet supports the techno-typological criteria applied for defining artefact categories (cores, debitage, tools), as well as the proposed differentiation of carinated ‘core’ types. The non-carinated cores in Hayonim Cave are characterised by a high variability in typology and reduction concepts. A curious similarity to the Levallois-concept is observed on some of the flake cores. It is therefore suggested that the frequent recycling of Middle Palaeolithic artefacts in the Levantine Aurignacian might have given the Aurignacian flint-knappers the opportunity to study the Levallois concept and apply an approximation of it in their own core reduction strategies. The notion that Palaeolithic flint-knappers actively observed former technological systems through the discarded artefacts directly opens up a new trajectory for the understanding of lithic reduction concept permanence. The conceptual diversification and variability in Hayonim Cave D indicate a highly dynamic period in the Levantine Upper Palaeolithic which increased the adaptive potential and promoted a rapid cultural change.

## Introduction

Lithic techno-typology is considered a stable marker of cultural coherence and is commonly used to group industries or techno-complexes together. However, for prehistoric people, flint implements had mainly functional value, needed for tasks pertaining to various aspects of daily life (subsistence, maintenance, care taking, etc.). Those people were unaware that in the distant future archaeologists would use these items to assign them to ‘groups’ or ‘cultures’.

Lithic technological knowledge, hence the strategies employed in the fabrication of stone tools, are considered a more reliable cultural marker than typology, i.e. the design and shape of the tools themselves. While at the beginning of Palaeolithic research, the major timeframes have been divided according to typological characteristics, or the *fossil directeur*, these typological distinctions did actually capture changes in the technological method. Thus, they reflect the difference between core tools in the Lower Palaeolithic, the Levallois point in the Middle Palaeolithic and blade tools in the Upper Palaeolithic. Apparently, from the beginning of research, technological principles were recognized as stable and subconsciously used for the distinction between the major Palaeolithic stages. Technological knowledge is often expressed under the notion of ‘concepts’, which are *recipes* or *mental road-maps* to transform a flint nodule into the core that can yield the desired blanks or products. Their implementation can be observed in lithic operational chains, i.e. the steps or gestures [1] employed to actively change the form of the modified flint item. One of the main drivers for innovation in lithic technology during the Palaeolithic is the need for weapons–the blanks for which are most often the central products of the chosen reduction concepts (e.g. Levallois points/flakes of the Levallois concept, el Wad points of the Ahmarian blade production, etc.). Changes in lithic weaponry requirements and thus morphology changes often co-occur with changes in lithic technology and core reduction concepts, although current research is unable to deduce which is the cause and which is the consequence.

Studies of lithic concepts can be used to reconstruct trajectories of technological knowledge, direction of innovation transmission and underlying exchange and contact systems. The present study attempts to do exactly that with the investigation of carinated elements in the Levantine Aurignacian, a distinctive feature of Aurignacian industries overall.

The early Upper Palaeolithic (UP) in the Levant comprises the Ahmarian (earlier; [⁠^2^–^5^]) and the Levantine Aurignacian (later; [6]), implicitly or explicitly assumed to be related to the European Aurignacian [7–14]. Until the 1980s it was implicit that the local UP comprises exclusively Aurignacian industries and it is only with time, after new data emerged, that re-evaluation of some previously-discovered assemblages revealed the existence of yet another UP techno-complex, namely the Ahmarian. Since then, the two-traditions model [5, 10, 15] has shaped lithic research and discourse in the region, being relevant not only for the early UP but also for later lithic assemblages up to the Glacial Maximum.

Commonly, the Ahmarian is considered an endemic Levantine techno-complex, divided into Northern and Southern varieties [16]. The understanding of it as an endemic techno-complex stems from the findings in Boker Tachtit, where an *in situ* technological change from the Middle Palaeolithic to Upper Palaeolithic blade technologies could be observed [17].

The Aurignacian on the other hand appears considerably later (although dating issues exist for both complexes) in the Mediterranean zone of the Southern Levant and is considered an ‘intrusive’ complex, tentatively connected to migrations, cultural contacts, or innovation transfers. Only a few sites with Levantine Aurignacian assemblages are currently known, yet there are several later occurrences assigned to an ‘Aurignacian tradition’, e.g. the ‘Arqov-Divshon’ and the ‘Atlitian’ [18–20]. The European Aurignacian is dated to ca. 42.5 ka [21] and has subsequently been divided into three chronological subphases, an Early, Middle and Younger Aurignacian [22, 23]. An additional earlier phase, the Proto-Aurignacian [24], is contested [25], as are the interrelationships between the three phases and the significance of regional variants [23].

Ahmarian lithic technology is rather straightforward and reasonably well-understood. Several studies have been conducted with detailed assessments of the operational chains for the production of the desired blanks. These are narrow, straight and pointed blades/bladelets, the blanks for the main projectile type, the el-Wad point. The by-products of their operational chain are used for the production of other relevant tool types, burins, endscrapers, etc. [26–29].

While the Levantine Aurignacian as such was defined much earlier, it has been far less intensively studied than the Ahmarian. Several of the known sites have been excavated in the early years of local prehistoric research and are either completely emptied (e.g. el-Wad) or problematic to reassess/access (e.g. Ksar Akil due to political issues in Lebanon). The lithic conceptual knowledge of the Levantine Aurignacian has largely been unknown, with only a few somewhat detailed studies [9, 26, 27] published. Currently, there are some on-going, in-depth studies (e.g. [14, 30–32]) hoping to amend this situation. The present paper is one of them.

### Carinated items—Tools or cores?

Carinated technology is a hallmark of the European Aurignacian, also playing a significant role in the Levantine Aurignacian. From the Levantine Aurignacian onwards, carinated forms can be found in the material record of the region well into the Epipalaeolithic (23,000–11,500 BP). Recently, an innovation transfer of this technology from the carriers of the Levantine Aurignacian onto the local Ahmarian population has been suggested, leading to a mixed technological appearance of Post-Aurignacian cultures and the subsequent variability in technological systems [32–35].

While technological similarities between the carinated technology in European and Levantine Aurignacian are often proposed, repeated, and discussed, so far, no targeted effort has been made to prove it directly on the material. This is the aim of our current project whereby the first step is the meticulous analysis of the material from Hayonim Cave presented in detail here. The following publications will focus on the comparison with European materials, both from hand-on new analyses as well as from the literature. This step-by-step approach is of necessity, taking into account the complexity and minutiae of detail required for the assessment of single individual assemblages on the one hand and the complexity of the comparative protocol on the other. The resulting data and interpretation overwhelmingly exceed the scope of one publication. Accordingly, this is only the initial endeavour to tackle the puzzle of Aurignacian migrations and/or innovation transfer at the beginning of the UP.

Carinated artefacts, usually considered as either burins or endscrapers, have long been thought to be tools comparable to non-carinated burins or endscrapers, aimed at planing/scraping activities and/or hide-working [36, 37]. Thus, they have been included in commonly used European and Levantine tool type lists (e.g. [38–43]). However, recent research in western Europe has fundamentally changed the European traditional view on carinated forms. Detailed technological studies accompanied by refitting have revealed that carinated burins and endscrapers are mainly cores for the production of bladelets. The operational sequences for the preparation, reduction and maintenance of these cores have been understood in detail entailing a detailed typology of the associated products [37, 44–51]. There is therefore little room for doubt that in the European Aurignacian carinated forms served mainly as bladelet cores.

In the Levant carinated forms are most commonly recorded as tools, albeit the inherent problem of addressing them has been a topic of scholarly debate for many years [42, 52–54]. Recognized issues include the problematic differentiation between carinated items and bladelet cores on the one hand and their typological distinction from regular burins and endscrapers on the other hand. Different schools of research address and define carinated tool forms differently [53] and thus no region-wide comparison even of quantitative aspects is currently possible without a total reassessment of all material. Targeted technological studies comparable to the French ones (e.g. [44]) are currently rare and have been conducted so far only on the carinated endscrapers from Hayonim Cave [55]. Therefore, presently neither the function nor a precise typological distinction of carinated forms–tools or cores–is clear. A few attempts have been made to address this issue [34, 35, 53], but these have not yet led to a fundamental re-evaluation of the carinated items in Levantine Aurignacian contexts.

### Innovation transfer of a novel technology

If indeed the technology of carinated core reduction has been introduced into the Levant externally via an innovation transfer, it has taken place in a framework of cultural organisation of the daily routine as well as on group and individual levels in terms of social learning and innovation transmission and acceptance. These two variables, cultural organisation and innovation transmission, interact on different levels shaping the potential of both, innovation transfer [56] and archaeological visibility.

Levantine UP hunter-gatherers have been repeatedly observed to follow a residential system adapted to opportunistic exploitation of the immediately surrounding resources in a temperate Mediterranean environment [40, 57, 58] targeting specific patches of resource abundance [33]. This subsistence pattern has been suggested to have formed as the result of a fragmented but especially favourable environmental situation allowing for close range exploitations of oasis-like environmental niches. The patchy resource availability might have led to cultural fragmentation and thus the development of small regional subgroups during the course of the later UP (as e.g. developed in Fazael IX, [20], and Nahal Rahaf, [30, 32]), exploiting different resource patches with only intermitted cultural contact. The spread of carinated technology on the one hand and the different material records on the other hand were suggested to reflect repeated fragmentation and cohesion processes of the various distinct groups with a differently developed application of carinated technology [33].

### Hayonim Cave

**Fig 1:**
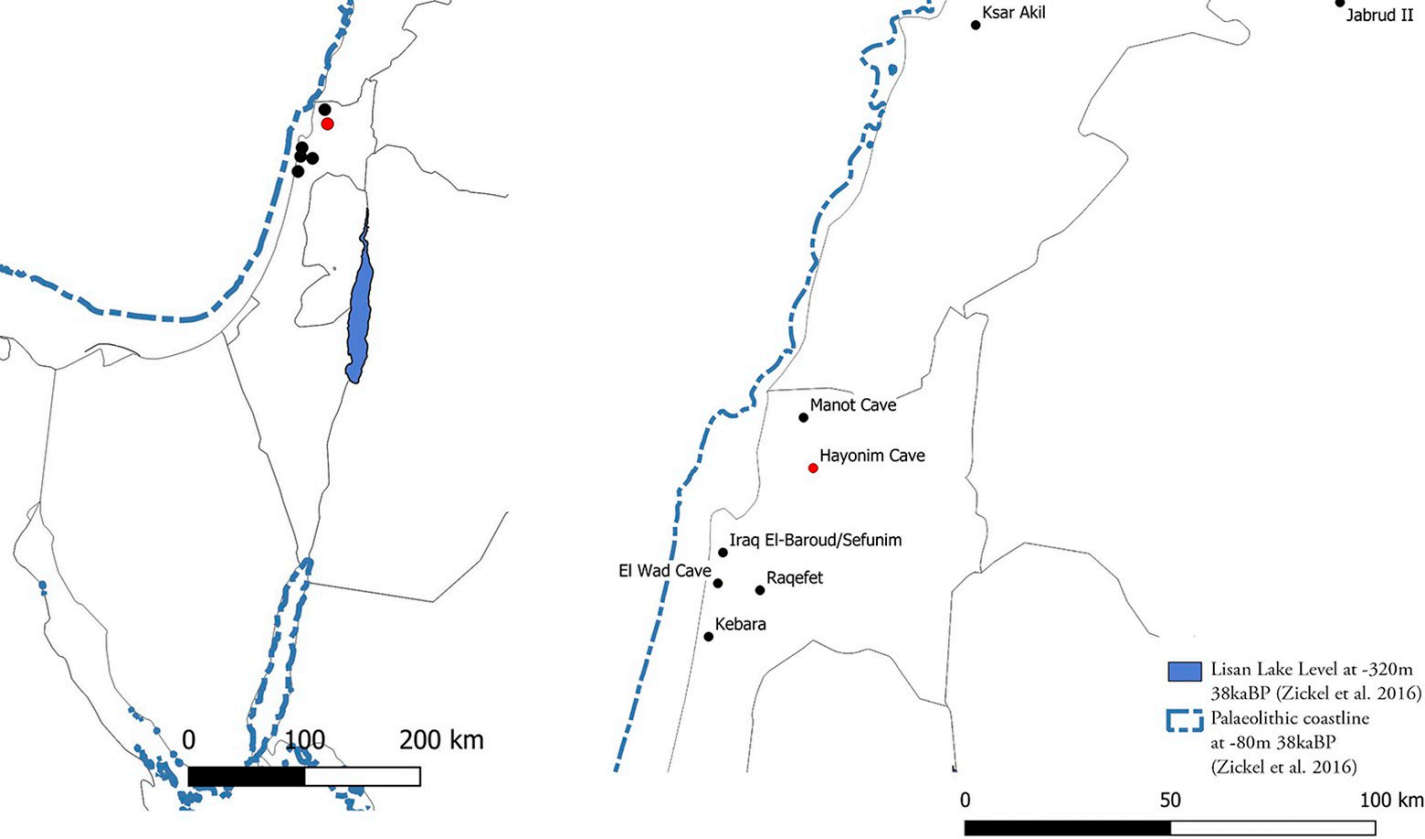
Location of Hayonim Cave. Depicted are the reconstructed shore-line and Lisan lake level at 38ka [117].

**Fig 2:**
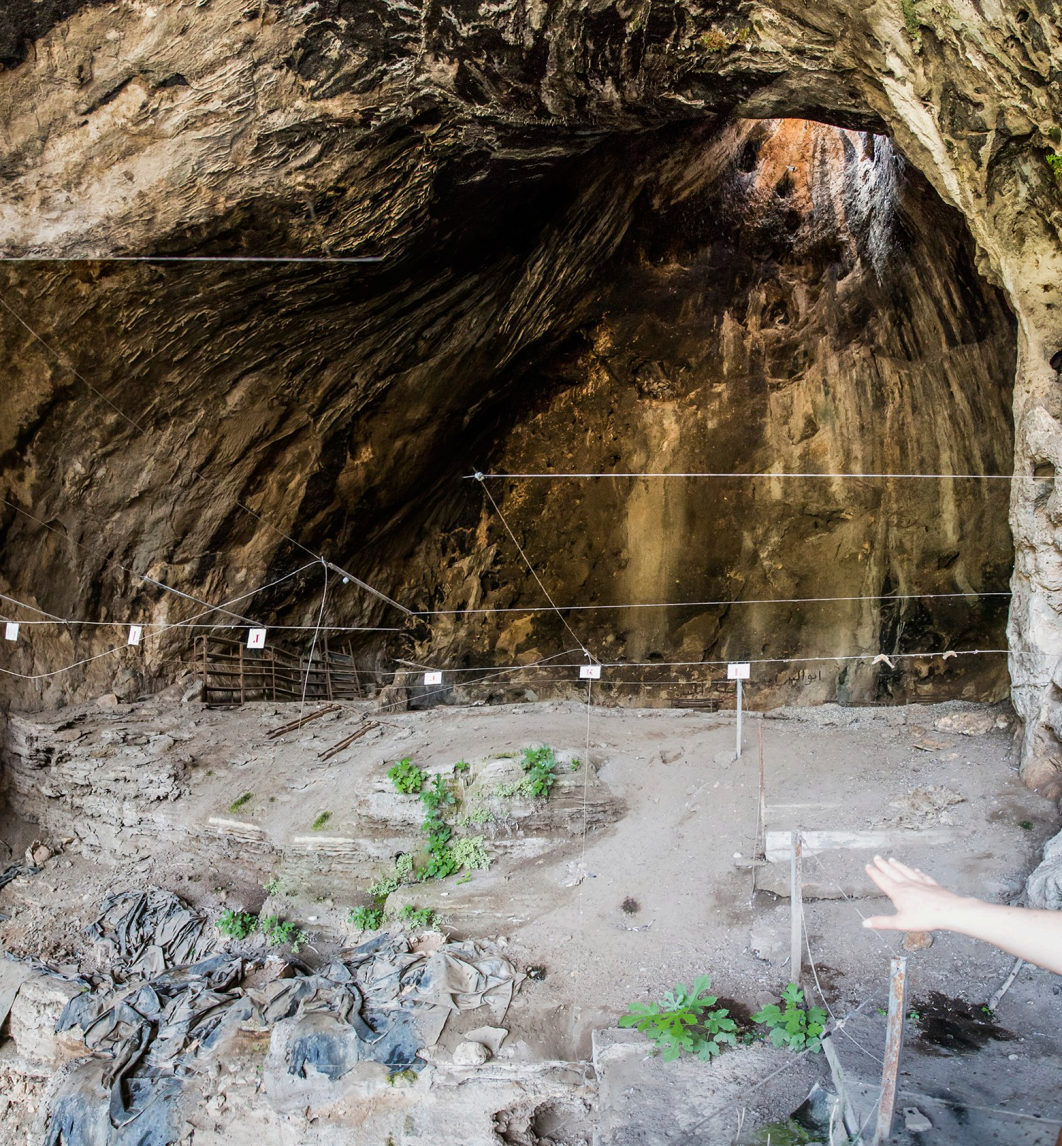
Hayonim Cave. Back chamber with chimney, profile of main excavation trench in front.

Hayonim Cave lies in the Western Galilee on the northern flank of Nahal Meged, one of the many wadis draining the Galilean hills towards the Mediterranean Sea (Fig 1). It is an active karstic cave with originally five different chambers of which three have collapsed and are now eroded. The interior of the cave consists of a large frontal chamber and a smaller posterior chamber which opens up to a chimney (Fig 2). The frontal chamber contains the main excavation trench yielding the material analysed in this study. The cave interior had been systematically excavated by Ofer Bar-Yosef between 1965 and 1979, the Terrace was excavated in the 1970s and 80s by Donald Henry and François Valla respectively. Further excavation seasons in the cave followed (1992–2000, 2022), but the studied assemblage, deriving from the first series of excavations, is the only material currently available [59]. No Aurignacian (or other UP) remains were observed in any of the following seasons.

The site yields occupational remains of the Natufian, Kebaran, Levantine Aurignacian, Mousterian, and Acheulian. The Aurignacian material was analysed and published by Anna Belfer-Cohen and Ofer Bar-Yosef [9].

Excavation techniques included material recovery in 5cm spits following the natural stratigraphy in quarter square meters and wet sieving to recover the small fraction material. Tools, cores, bone tools and other special finds were single plotted by hand.

**Fig 3:**
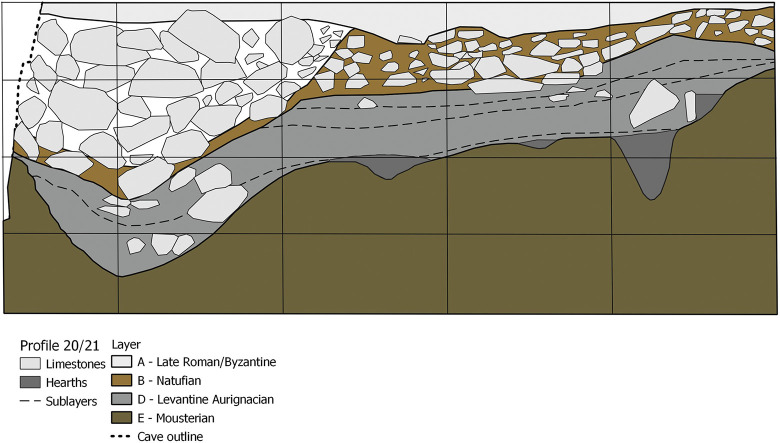
Stratigraphy of Hayonim Cave.

**Fig 4:**
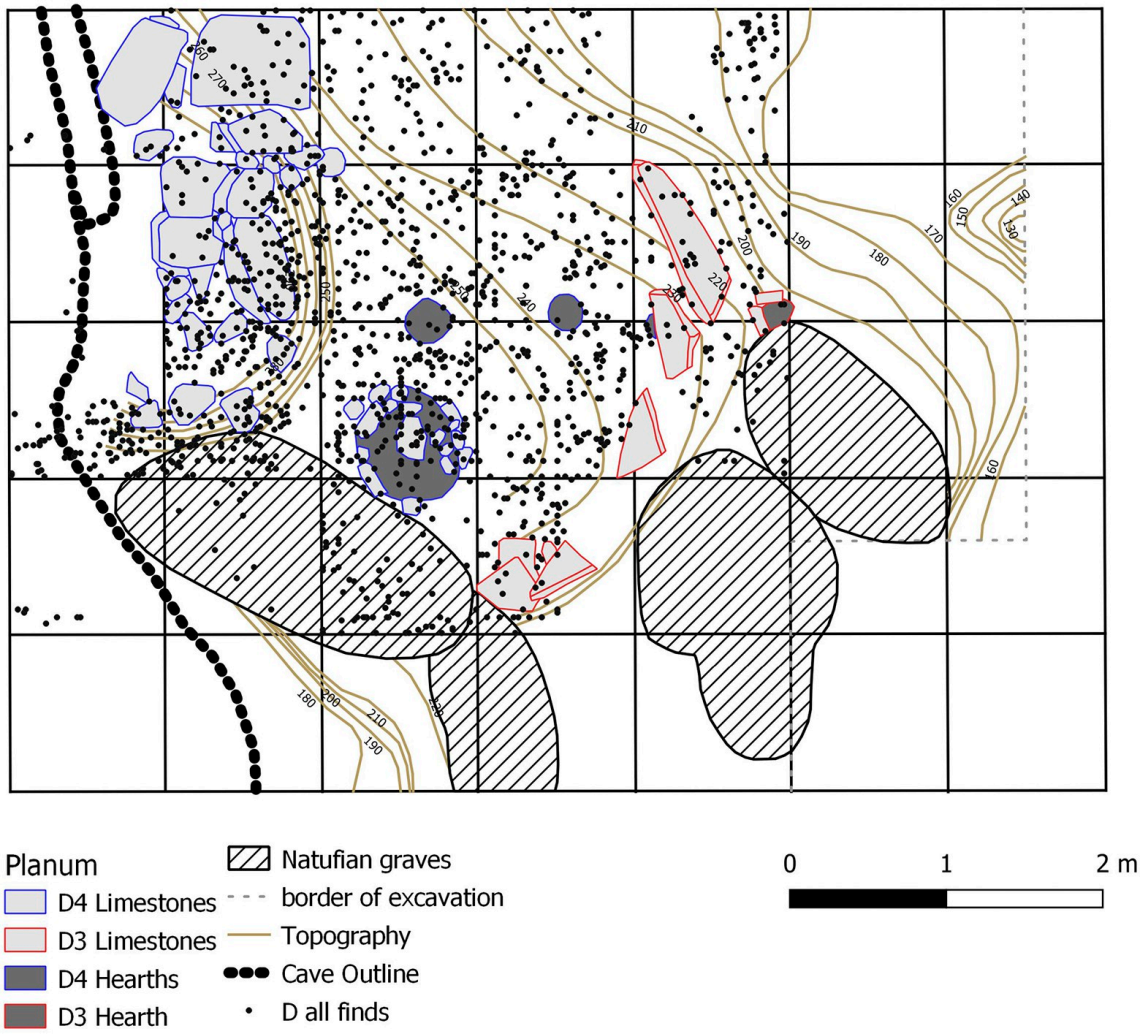
Hayonim Cave D Planum. Evident features and all single plotted finds (after [9].

The stratigraphy of the main excavation trench (Fig 3) includes four layers, of which layer D comprises the Aurignacian occupational horizons. It consists of a light greyish loam and was subdivided into four sublayers D1–D4. Evident features (Fig 4) were recognised during the excavation including five hearths, a ‘kitchen midden’ and a number of limestone blocks indicating construction activities. In total the assemblage of Hayonim Cave D consists of 17,500 lithic artefacts as well as a well-developed bone and antler industry. Furthermore, the site provides evidence for symbolic behaviour with the presence of animal tooth pendants, incised gazelle scapulae and the only figurative limestone engraving from the Levantine Aurignacian [9, 60]. The assemblage has been ^14^C dated to a range of 19,000–34,000 years calBP. The dates were obtained early in the research history, predate modern AMS protocols and are therefore problematic to assess. A more detailed discussion of the matter and the placement of the assemblage within the Levantine Aurignacian chronology will be presented in the Discussion part of the paper.

## Materials and methods

### The Hayonim Cave lithic assemblage

The lithic assemblage of layer D, stored at the Hebrew University of Jerusalem, was sorted, and divided according to the layer D sub-phases, carinated reduction sequences or non-carinated ones. All cores, carinated items and a sample of the bladelets (due to time restrictions) were subjected to an attribute analysis and closer investigation. The remaining bladelets were counted and sorted according to their place in the operational sequence and their product type (see below). Material belonging to carinated reduction sequences includes all carinated items, all retouched and blank bladelets and all identified preparation and maintenance products stemming from carinated reduction sequences. Accordingly, all the rest (debris, chunks, debitage of other reduction sequences and non-carinated tools) were not recorded. If the attribution was doubtful, the artefact was not included in the analysis. In this manner, 757 items of carinated reduction sequences were subjected to a detailed attribute analysis, a further 887 bladelets were counted and 239 non-carinated cores were classified, so the total analysed assemblage comprised 1883 items. A large number of artefact illustrations was done to prove the drawn conclusions directly on the material [61] and provide a data base for future comparisons.

### Operational chain reconstruction

**Fig 5:**
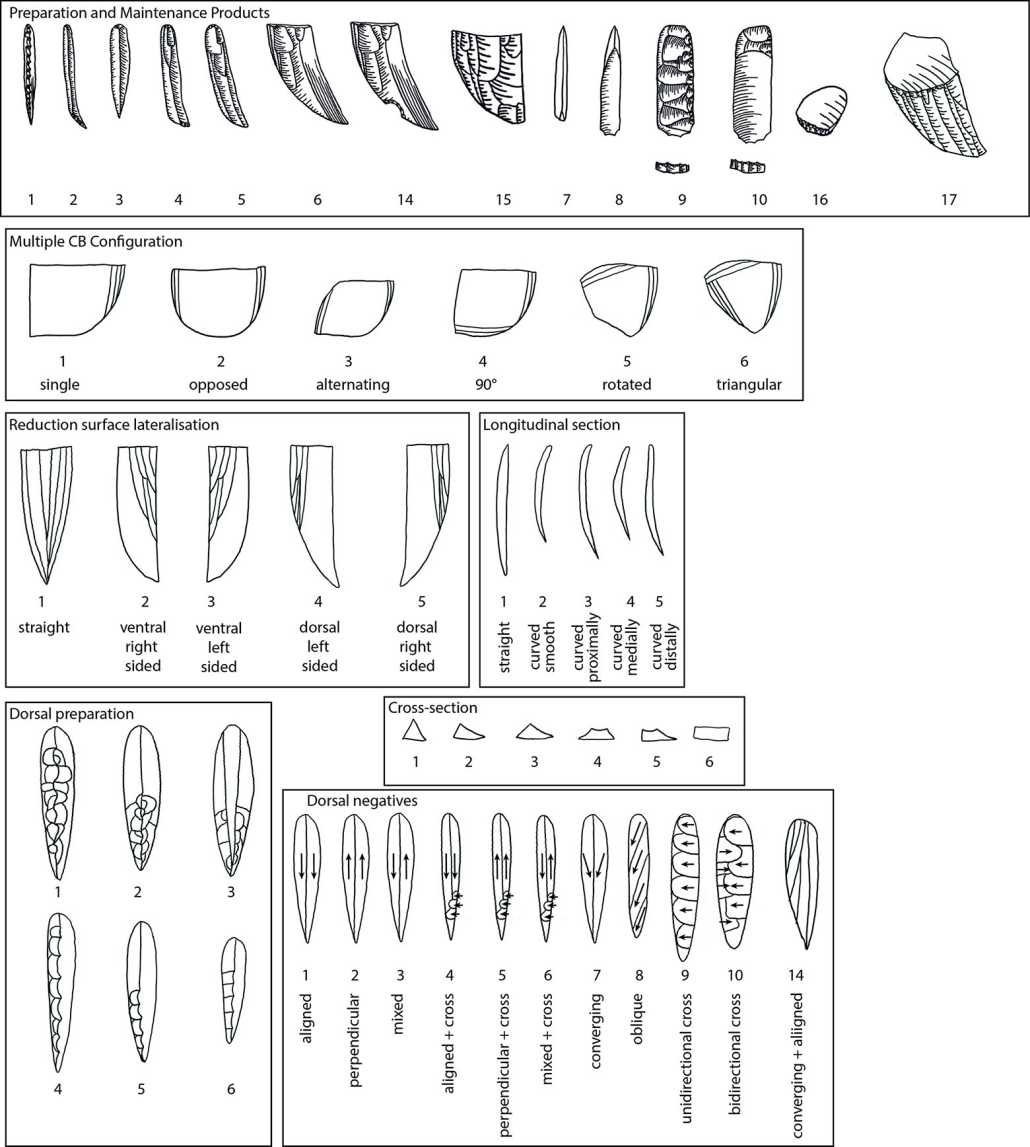
Code sheet for the analysis.

Several lines of evidence can be used as arguments in the debate on the function of carinated items [53, 54]. This study focuses on the reconstruction of the lithic operational chains of carinated reduction in order to find out whether there is a coherent and systematic approach apparent in the production of carinated items. The carinated items were *a priori* divided into two varieties, carinated ‘burins’ and ‘endscrapers’, where the distinction is drawn according to the orientation of the carinated face. If it was in a 90° angle to the ventral face of the blank, it is a carinated ‘endscraper’, if it was on the same horizontal it is a carinated ‘burin’. Previously used definitions as e.g. by Movius und Brooks [62] or Bergman [63] were reviewed and discarded. We feel that these definitions–all made prior to the new assessment of carinated items–are overly metrical and thus fail to capture the different core exploitation stages correctly. In our eyes, a technological definition, based first on the orientation of the carinated edge, and second on the presence of preparation scars, is a more reliable route. This practice is also standard in other core definitions as there is no metrical definition for e.g. Ahmarian narrow-fronted cores or Levallois cores [26, 27, 64]. The primary assumption to explore was the potential function of the carinated items as bladelet cores. An additional or exclusive function of these items as tools can be investigated only through a use-wear study, which was beyond the scope of the present study. Thus, only the arguments for and against a core-function were investigated. To this end several approaches were used: a classic reconstruction of the operational chains preceded all further analyses, i.e. identifying preparation, maintenance, and target products. The items assigned to the operational chain stages were categorized typologically. Classical preparation and maintenance products of Western European carinated reduction concepts [37, 44–51] were identified and type coded (first box in Fig 5). An attribute analysis was conducted (for categories see S3 File) to understand knapping mechanics and identify a systematic approach to core reduction, if present. This attribute analysis additionally targeted the understanding of the correlation of cores and target products through the identification of core and product size, lateralisation, and twisting. The lateralisation is determined by the twist of the flaked off bladelets. Thus, the core is looked at from the platform, a twist to the right is ‘right sided’, a twist to the left is ‘left sided’. It is further differentiated if this twist turns towards the dorsal or ventral face of the ‘core’ in the case of a ‘burin’. If a bladelet or preparation/maintenance product laterally shows remains of a former ventral surface on its dorsal face, it means that it derives from a core-on-blank or a carinated ‘burin’. Such items provide evidence for the possible core function of carinated items. Due to the extremely small size of many of these bladelets, it can sometimes be very hard to see if a surface is a ventral or a dorsal one, and it is very likely that this feature is underrepresented in the analysis. The length of the cores ‘reduction surface’ is measured as well as the length of the last removal negative and the type of fracture termination. These attributes are used to understand reasons for core abandonment and to identify the wanted size of the target products. The negative length can be compared to the length of the target blanks, i.e. bladelets, as well as to that of the retouched bladelets to investigate a correlation of the three groups of artefacts. The elongated material was divided into blades and bladelets, with unretouched bladelets defined as less than 12mm wide [65]. We did not use a definition of ‘microblades’. The retouched bladelets were further subjected to a typological description based on the Levantine type lists ([33] type list see S2 File). Determinations according to European type lists (e.g. [50, 51]) were attempted but were too often unachievable as only complete bladelets could be included. Nonetheless, whenever possible, data, defined according to Le Brun-Ricalens [50] were recorded, and if suitable, will be included in future discussions and comparisons with European material.

### Terminology

Carinated items are commonly defined as blades or flakes, showing one or more parts with steeply converging knapping scars forming a ‘keel’ (carination, Fr. *carène*). The location and direction of this carination determined the formal terminology employed here. Items with a carination orthogonally to the ventral face of the blank were termed ‘carinated endscraper’ while such with the carination parallel to the ventral face of the blank were considered ‘carinated burins’ [50, 51]. This distinction mirrors the common way of addressing burins and endscrapers in lithic assemblages. For the purpose of this study the production of carinated burins and endscrapers is considered as two separate operational chains from the onset, each of which may or may not be subdivided into different sub-chains internally.

The carinated items will occasionally be addressed herein as ‘cores’ to facilitate and increase the readability of the technological description, as the aim of this study is to investigate their potential function as a core. Likewise, common terms to describe core morphology and maintenance (e.g. ‘platform’, ‘reduction surface’, ‘core tablet’) are used, again to increase readability and to streamline the descriptions. The items are divided into those produced during the reduction *vs*. those produced during the preparation and maintenance stages. Reduction bladelets are separated into target and by-products, considering the ‘target’ ones to be the technological aim and the central product of the operational chain setup. By-products originate from the lateral parts of the reduction surface and are not the focus of reduction. Classification as target products does not necessarily imply a correlation to ‘tool’, although it is often the case. The definition as target product is related to the core-setup and the ‘target’ product is the central item of reduction.

A close observation of the material is required for the definition of operational chain stages and the attribution of associated products. Here an 8-stage operational chain is used, and each lithic artefact is sorted to one of those stages. Due to the closely related nature of the two major carinated operational chains discussed here, it is often not possible to differentiate between the products of the two chains, namely that of the carinated burins and the carinated endscrapers. While most of the specific preparation and maintenance products can be attributed to either of them, the bladelets can originate from both sequences and cannot be differentiated. It is thus not possible to separate the entire carinated assemblage neatly into one group, definitely connected to burins, and the other, definitely connected to endscrapers. If one does wishes to evaluate the relative importance and frequency of one chain *vs*. the other, only the ratio between carinated burins and endscrapers can be used for comparisons. Items are sorted into the different stages according to the following criteria (adapted from [51]):

I–Raw Material Acquisition: flint nodules or blanks that fit the desired core shapes. As only the carinated part of the assemblage was re-assessed, this stage was not examined, it is retained here as a reminder of its importance to be applied whenever possible.
II–Initial Opening: completely cortical items, triangular first removal burin spall, first core tablets, Janus bladelets with two ventral faces.
III–Decortication: items with more than 50% cortex and no laminar scars indicating a systematic previous reduction.
IV–Preparation: early plain core tablets with a preserved ridge, primary type Thèmes core tablets, fully prepared crested bladelets. Preparation products generally do not show signs of previous systematic reduction, appear crude and are difficult to reconstruct on a core.
V–Reduction: bladelets with less than 50% cortex coverage and negatives of previous systematic reduction. ‘Target’ bladelets are twisted or straight, blunt or pointed, of regular appearance. ‘By-product’ bladelets are irregular, lateralised, hinged on their dorsal face or are ‘overshots’. Typical for by-product bladelets is the observable evidence for linear systematic reduction on the half of the bladelet previously close to the centre of the reduction surface, while the other half is covered by remains of preparative actions (e.g. Plates 1.6; 2).

**Plate 1:**
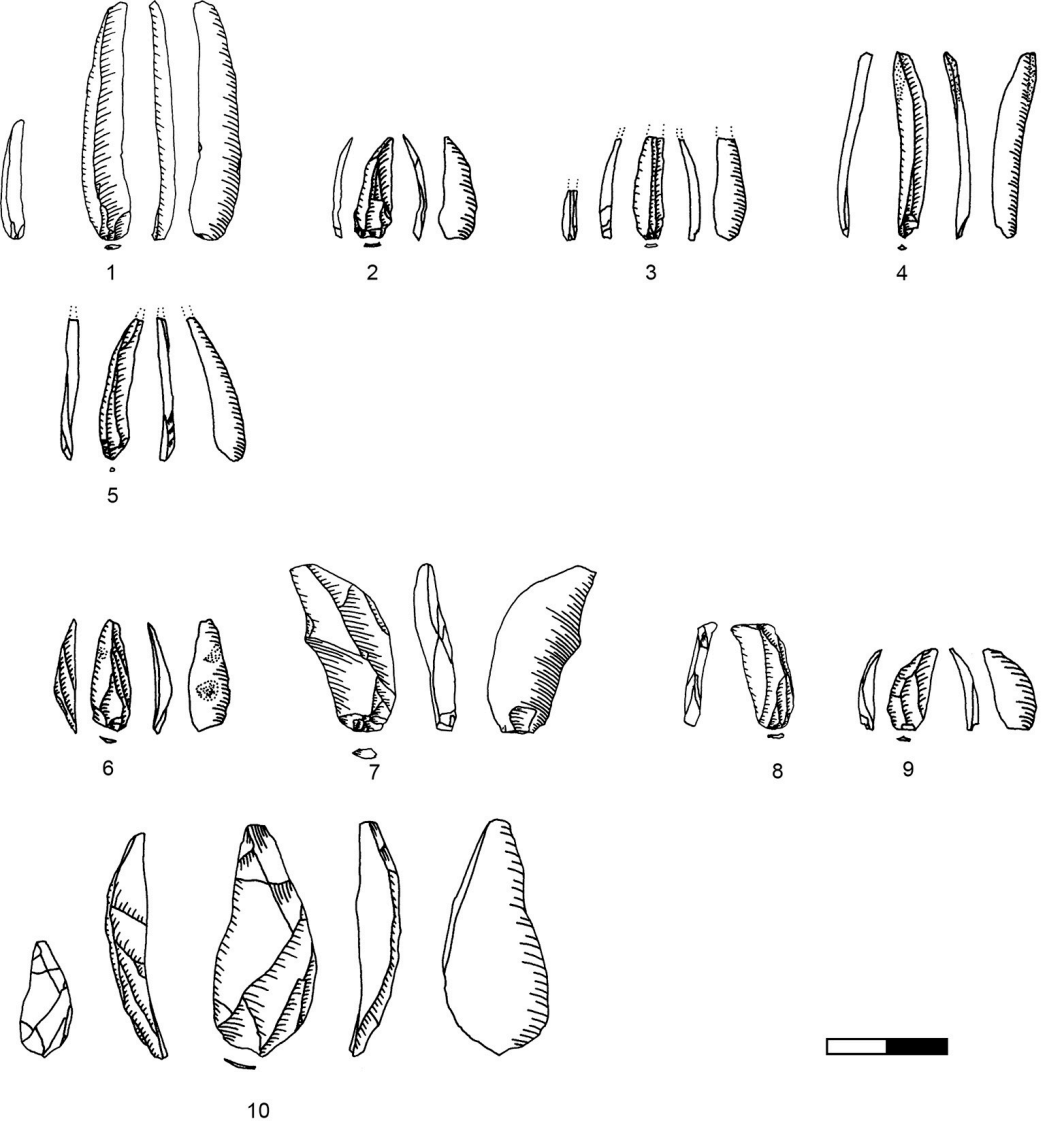
Bladelets and core trimming elements. 1, 4–7, 10 D3; 2–3, 8–9 D1-2. 1 enlarged, original size on the left.

**Plate 2:**
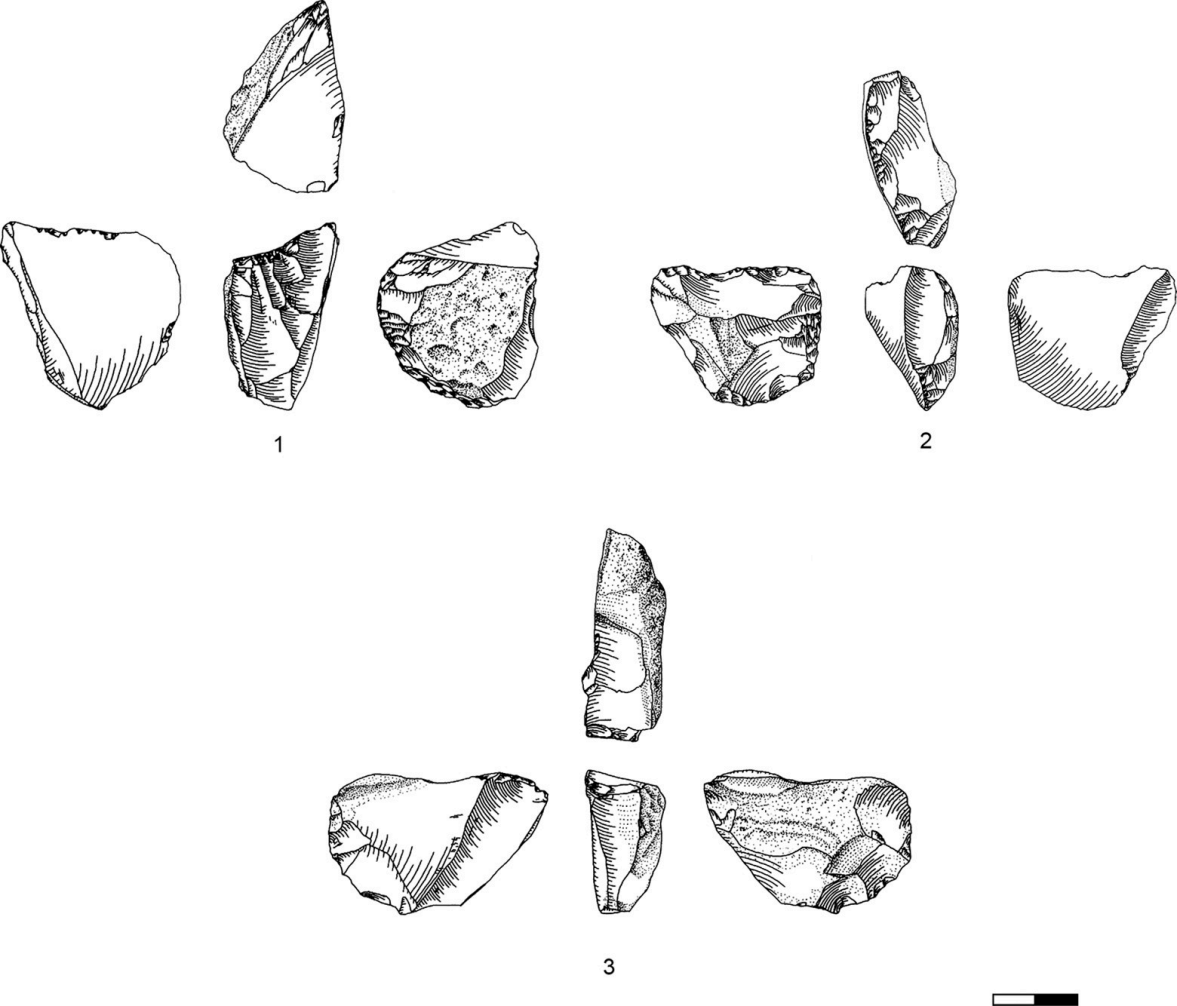
Carinated burins. 1 D1-2; 2–3 D3.

VI–Maintenance: maintenance products are the opposite of preparation products. They show signs of previous systematic reduction and portray faults or defects such as hinges, or other knapping errors preserved on their dorsal face. They include secondary core tablets, plain or of the Thèmes type, neo-crested overshot bladelets, complete ‘caps’ of carinated endscrapers struck off, or re-creation of notches for nosed endscrapers.
VII–Discard: all cores, core fragments or carinated items and preforms of them. Preforms are understood as blanks fitting the need for core reduction with only initial preparation, sometimes called ‘tested cores’.
VIII–Use: all retouched bladelets.

**Fig 6:**
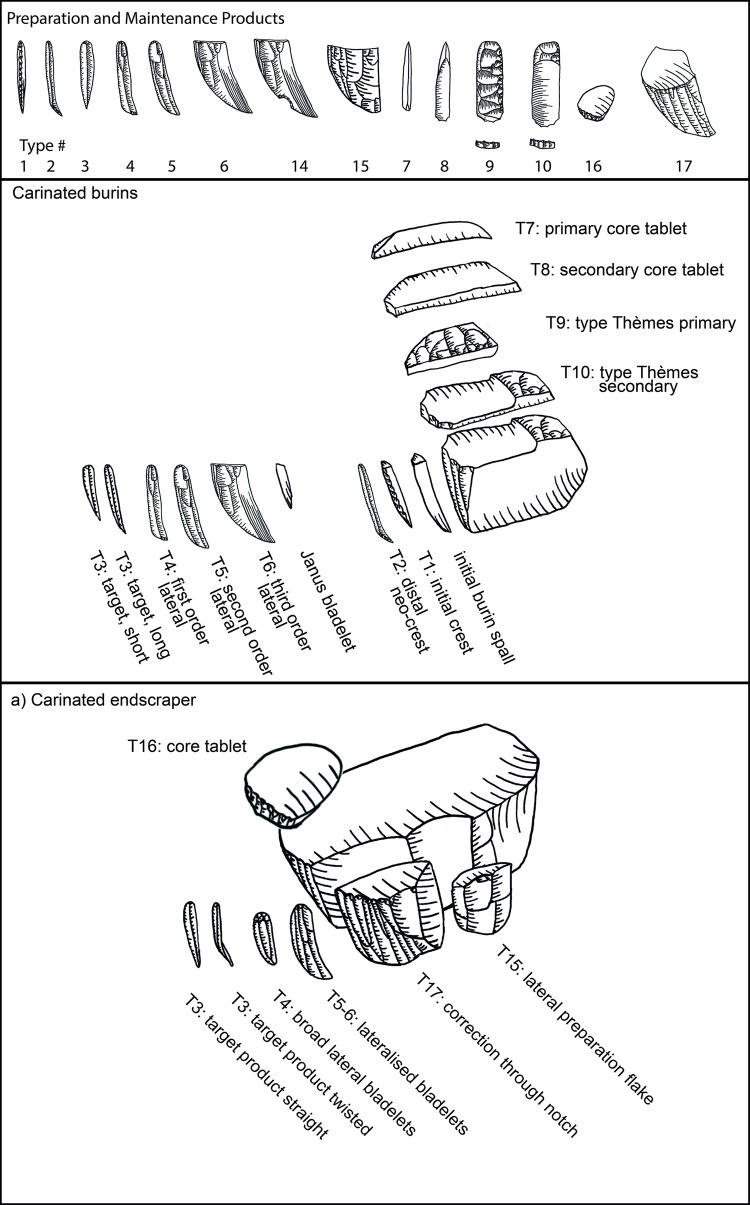
Overview of preparation and maintenance types and their place in the operational chain (after [51]).

Following is a detailed classification of the preparation and maintenance products discussed here (Fig 6, ‘T1’ and following reference to the types mentioned below). It is needed in order to facilitate their recognition in other assemblages and to clarify our own attribution and definitions. As a general note, it should be understood that steeply triangular products (i.e. thicker than wide) with a narrow ventral face and a protruding dorsal ridge are connected to the initial removal from the edge of a blank used as the core. Triangular products which are overall flatter (hence wider than thick) can originate from all later stages of the operational chain.

**Type 1 initial crested bladelets:** these items usually belong to the preparation stage and are characterised by a steeply triangular cross-section and mostly a continuous lateral cresting of the ridge. This crest can be uni- or bi-lateral and serves to form the front of the core-blank to ensure a smooth detachment of the first opening bladelet spall. Very often they are continuously or distally curved. On one side of the dorsal ridge the former ventral face of the blank is visible, and on the other, whichever surface the core-blank had on its own dorsal face, negatives, or cortex. These items most generally belong to carinated ‘burin’ operational chains.

**Type 2 distally crested bladelets:** distally crested bladelets stem from preparation or maintenance stages, more common in the latter. They are characterised by a triangular (preparation) or trapezoidal (maintenance) cross-section and a distal crest, which is more often unilateral (mostly during maintenance) but can be bidirectional. Their goal is the re-establishment of the distal curvature of the reduction surface, and they are generally comparable to regular crested blades in non-carinated blade/let core reductions. These items most generally belong to carinated ‘burin’ operational chains.

**Types 3–6:** These types include all bladelets originating from the reduction stage of both (‘burin’ and ‘endscraper’) carinated reduction sequences. They can be differentiated by their degree of lateralisation and the presence of core parts which do not have lamellar negatives from the previous reduction such as the core´s ventral face, cortex or dorsal (either previously present or core-preparation/maintenance related) negatives (Plate 3.1 illustrates remains of the previous ventral and dorsal faces, and Plate 3.2. the previous ventral face and a cortex covered dorsal face).

**Plate 3:**
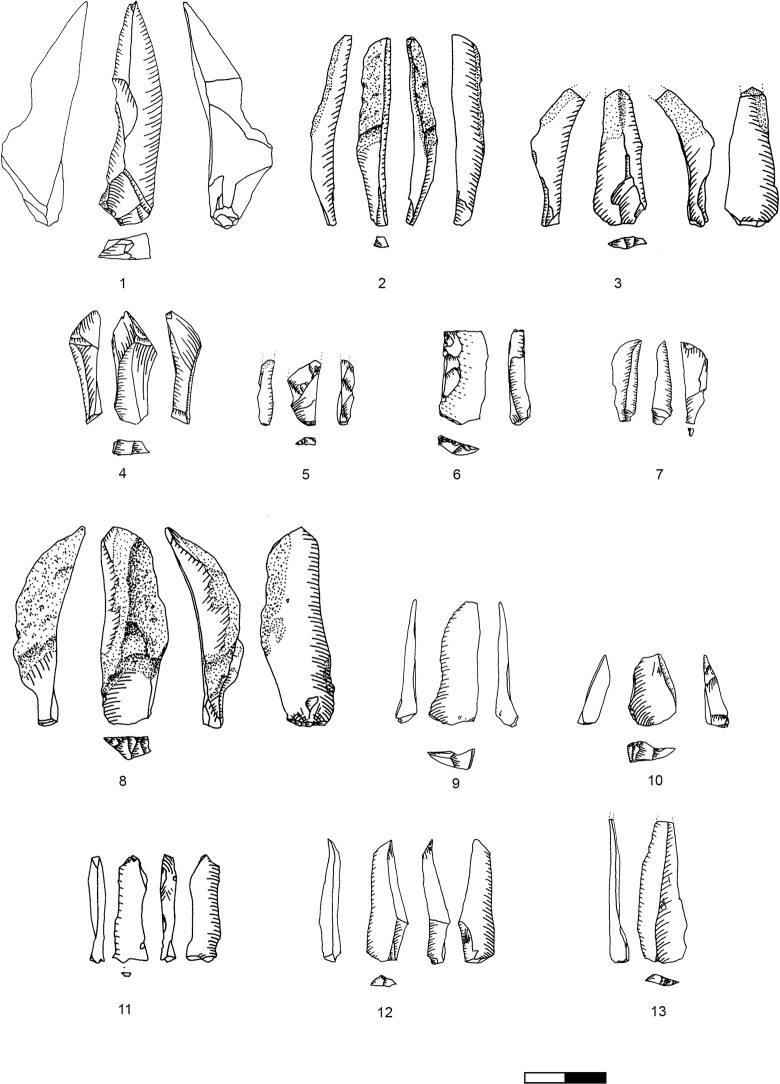
Core trimming elements. 1, 3–5, 8, 13 D3; 2, 7, 9, 11 D1-2; 6; 10, 12 D.

**Type 7 initial core tablets:** these items belong to carinated ‘burin’ reduction sequences and can be considered the counterpart to the initial burin spalls. The initial core tablets provide access to the platform, while the initial burin spalls open up the reduction surface. Both belong to the preparation stage. The initial core tablet does not have lateral faceting as preparation activity, which is its main distinction from the following Type 9. Its morphology is generally triangular and preserves large parts of the original blank edge chosen as the flaking platform of the carinated item. Differentiation between the initial core tablet and initial burin spall can be difficult and it is not always possible. Generally, for the initial core tablet the usual technological characteristic of previous reduction negatives on its platform does not apply, as their placement in the operational chain must predate any systematic reduction activity. Initial core tablets tend to be shorter, straighter, and broader than initial burin spalls.

**Type 8 secondary core tablet:** this item underlies the initial core tablet and can originate both from the preparation and from the maintenance stages of the reduction sequence. Items from the preparation stage usually do not have negatives of previous reduction on their platform and their removal might have been necessitated due to either a hinged removal of an initial core tablet or the wrong knapping angle, which requires correction before reduction starts. These items rarely belong to the preparation stage, and none could be found in Hayonim Cave. In the maintenance stage, signs of previous reduction activities are visible on the platform and a platform angle correction is necessary due to continued exploitation of the reduction surface, just like in any other blade core concept. These items are usually rectangular in cross section, or at least have a right angle between the ventral face of the core tablet and the ventral face of the core. Its other lateral morphology depends on the shape of the dorsal face of the core.

**Type 9 primary type Thèmes bladelet:** this bladelet type is one of the most characteristic products of carinated ‘burin’ reduction sequences. It is typified by the presence of lateral faceting negatives which blunt and preform the edge of the core before the first detachment. Often, but not always, these faceting negatives are struck from the former ventral part of the core as it often provides the better knapping angle. Depending on the flaking of these bladelets, they can be removed prior to actual reduction, but they do not have to. If the created surface is suitably flat for the platform of the core, these bladelets can be removed sequentially later in the reduction process. Often, they are removed prior to the onset of systematic bladelet production, just like the triangular initial core tablets. The fracture termination of these items is often hinged, due to the ~90° knapping angle. Accordingly, traces of this initial faceting stage of the core preparation are often preserved on either the cores or the following core tablet removals.

**Type 10 secondary type Thèmes bladelet:** the secondary type Thèmes bladelet underlies the primary one (T9) and approaches the morphology of a standard core tablet. Its origin from a core with lateral faceting is only visible at the distal part of the tablet, where the previously hinged primary Thèmes bladelet left traces of the initial core shaping. The platform of the bladelet should yield negatives of previous reduction activities as it is more frequently a result of maintenance activities. Yet, as with the non-Thèmes core tablets, the timing, and the need for them can be immediate, and one item can follow the other, or there can be a significant pause between the removal of one tablet and another. The general relevance of the Thèmes bladelets is their status as indicating initial core preparation activities, which is not a common occurrence among standard burins. Here two faces, the platform, resulting in Thèmes bladelets, and the reduction surface, resulting in initially crested bladelets, are shaped in an angle to each other before the traces of shaping are removed, and the main reduction stage begins. The imbalance in reduction frequency between the two faces, their differential treatment, and their orientation to each other give indication to the function of the item as a core.

**Types 11–14:** Type 11 specifies cores, Type 12 other items which do not fit in the previous categories and Type 13 indeterminable items. Types 12 and 13 are not further discussed herein. Type 14 is a lateral flake or bladelet with remains of a distal notch. This notch is indicative of beaked burins, which have a distal stopping notch to control the length of the target bladelets. This concept is not used in Hayonim Cave but was implemented into the database to record it should it appear.

**Type 15 lateral preparation flake:** this item belongs to carinated ‘endscraper’ reduction sequences and is one of the few clearly indicatory items. It is characterised by lateral preparation negatives, usually non-lamellar on its dorsal face, and–most crucially–by the presence of remains of the ventral surface of the core on its platform. All carinated ‘endscraper’ products with larger platform remains can be identified via this criterion. The dorsal scar pattern can be quite variable, and it can belong to both preparation and maintenance stages. A distinction might only be possible via the presence of larger cortex cover on the dorsal face. These items do not bear bidirectional negatives.

**Type 16 carinated ‘endscraper’ core tablet:** this is an unmistakable, yet very rare item, exclusive to and highly indicatory of carinated ‘endscraper’ reduction sequences. Proximally, it bears on its platform the edge of the scraper cap with the cut-off lamellar negatives from the reduction surface of the ‘endscraper’ (i.e. core). Dorsally it shows the ventral face of the ‘endscraper’s’ platform. It can essentially be understood as a Janus-core tablet with two visibly ventral faces.

**Type 17 correction by notch:** this is another very indicative element of carinated ‘endscraper’ reduction sequences. Here an entire lateral side of the scraper cap is struck off from the ventral face/the platform of the ‘endscraper’/core, creating a new, and deep notch, hence a ‘nosed endscraper’. The resulting item is often very thick and shows large parts of the former ‘endscraper’ cap preserved, to the point where these items can be mistakenly recorded as broken ‘endscrapers’. This item is the result of a maintenance activity.

The items presented here are certainly not the only ones that can appear in carinated operational chains, but they are the most frequently observed and the most indicative ones. Thus, they can reliably be addressed in cross-assemblage comparisons. There are indeed other items such as the initial (uncrested) burin spall that serves to open up the reduction surface, or the Janus bladelet with two ventral faces that are not formally recorded here. In the context of carinated operational sequences their presence has a clear and firm place, yet they can similarly occur through other actions and processes.

### Statistical analysis

Several statistical methods are used to examine different aspects evaluated in the study. Simple significance tests are conducted to investigate if different item groups are statistically identical. The statistical significance threshold used in this analysis is p = 0.05 for the statement of general statistical significance. If higher significance thresholds are reached (e.g. 0.01, or 0.001) the result is being discussed as highly significant. A t-test is used on a subset of the assemblage after the confirmation of a normal distribution via a Shapiro-Wilk test. The same dataset is investigated via a simple linear regression analysis (e.g. [66, 67]).

Multivariate statistical methods were applied to explore the observed variability of the datasets to test for patterns of internal and external influence(s) on the data. The primary dataset was transformed into a distance matrix using the Gower-distance [68–70]. External covariables, preformed groups gained from the archaeological interpretation, were then tested for their explanatory significance using the *adonis* algorithm [71, 72]. Both steps of the analysis were conducted in *R* [73] with the packages *vegan* [74] and *FD* [75, 76]. The Gower distance measure is very useful for an archaeological approach as it can handle different types of input data, including binary, nominal, and categorical variables. For the application of the Gower distance the option ‘*podani*’ was used [77] in order to treat ordinal data. A Lingoes transformation was applied in order to transform the matrix into Euclidian distance [78, 79]. The *adonis* algorithm is a permutational multivariate analysis of variance which partitions distance matrices, such as the Gower distance matrix, among sources of variation and fits linear models to the distance matrix. A permutation test with pseudo-F ratios (p =) is computed to test for significance. It can also be described as permutational MANOVA. It is a very robust method and does not put any pre-assumptions on the distribution of the variables tested [71, 72]. The statistic result R^2^ will be given in percent of explained inertia.

In the present study the results of the attribute analysis are generally used as the primary dataset, while interpretations of the material or typological designations are used as covariables.

## Results

### Spatial patterns

**Fig 7:**
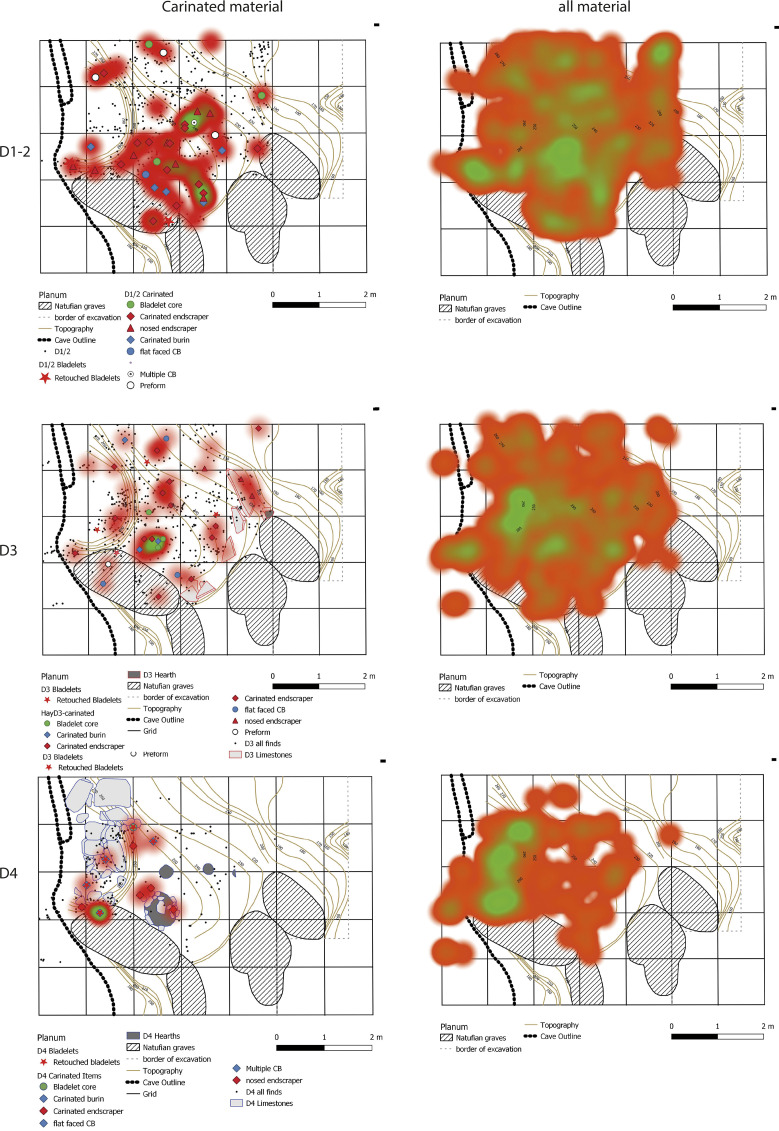
(a) Heatmaps of the total assemblage and only the carinated items for the sublayers of Hayonim Cave D. (b) Profile projections of all single plotted finds in three longitudinal sections through the Hayonim Cave D layer. Note: The profile projections include all finds in the two adjoining square metres, so the points follow the natural topography of the layer resulting in several of them being off the layer borders indicated in the profile.

The spatial pattern of all single plotted lithics and that of carinated material only (see below) can reveal potentially preserved activity zones of carinated core reduction. Two heatmap plots (Fig 7A) show that no clear activity zones are preserved in the different sublayers. The only clear concentrations present are found in sublayer D4 and are correlated to a sinkhole of the karstic system of the cave. In profile, clear layer separations are visible, as well as a slumping of the layers towards the sinkhole observed in all profiles (Fig 7B). Thus, the material possibly reflects either repeated occupation events intermingling or being compacted through trampling, or taphonomic processes acting on the material distribution, a ‘palimpsest’.

### Lithic assemblage

The analysis of the lithic material from Hayonim Cave D includes groups of items with different levels of information. All items recorded with three coordinates during the excavation were entered into the MS Access database and used for the heatmap plots (Fig 7A). This database comprises 4297 entries, which includes mostly cores, tools, bone tools, shells and other items already identified during the excavation.

A second, partially overlapping subset of items are those included in the present techno-typological analysis. The analysis comprises 1883 items in total (Table 1), of which 1644 belong to the carinated operational chains and 239 are the non-carinated cores. A sample of 757 items of the carinated assemblage, of which 122 are recorded in three coordinates, has been subjected to a detailed attribute analysis. 621 of these derive from the excavation in spits and thus can be attributed by quarter square meter, elevation, and level. The other 887 items have been sorted into the different products of the operational chain (first box in Fig 5) and counted. Items belonging to carinated operational chains comprise carinated ‘burins’ and ‘endscrapers’, bladelets, and the associated preparation and maintenance products.

**Table 1:** Composition of the analysed carinated assemblage.

	D1/2	D3	D4	D	Total
**Blade**	27	39	5	0	71
**Bladelet**	487	560	120	240	1407
**Flake**	14	23	10		47
**indet frgm.**	1	0	1		2
**Core**	100	96	33	10	239
**carinated item**	54	48	9		111
**carinated preform**	3	2	1		6
**Total**	686	768	179	250	1883
**Total recorded**					4297

The total assemblage recorded during the initial assessment of the Aurignacian assemblage [9] amounts to 17,479 items, but if chips and chunks are left out only 5575 items remain. Thus, the sample in this study comprises 10.8% of the total and 33.8% of the assemblage without chips and chunks. Since a study of a thematic subset as this one, usually does not encompass the total assemblage, the percentages given in the following discussion pertain only to the selected sample herein.

In the present sample, the carinated ‘endscrapers’ predominate, with 66 ‘endscrapers’ and 45 carinated ‘burins’ (59.5%). Conceptually, carinated ‘burins’ produce more blanks and more easily recognisable preparation and maintenance products as they generally include and allow for more invasive changes during core preparation and maintenance [51]. Carinated ‘endscrapers’ only produce very few typical preparation and maintenance products, while the majority are unrecognizable in the material record. In total, 207 preparation and maintenance products were analysed, of which a sample of 98 were analysed in detail during the attribute analysis (Table 2). Of the 520 bladelets in the attribute analysis, 111 were retouched (21.3%).

**Table 2:** Representation of operational chain phases of the entire carinated assemblage and bladelet assemblage composition as recorded in the attribute analysis.

	D1/2	D3	D4	D	Total
**I–Raw mat. acquisition**					0
**II–Initial Opening**	6	4	1	1	12
**III–Decortication**	7	9	1		17
**IV–Preparation**	8	48	3	8	67
**V–Reduction**	397	479	100	203	1179
**Target bladelets**	46	72	10		128
**By-product bladelets**	89	72	32		193
**Preparation bladelets**	18	22	8		48
**Maintenance bladelets**	19	15	6		40
**Retouched bladelets**	54	51	6		111
**Total bladelets**	226	232	62		520
**VI–Maintenance**	44	48	18	30	140
**VII–Discard**	54	48	9		111
**VIII–Use**	58	53	7	** **	118
**Total**	329	348	90		1644

**Fig 8:**
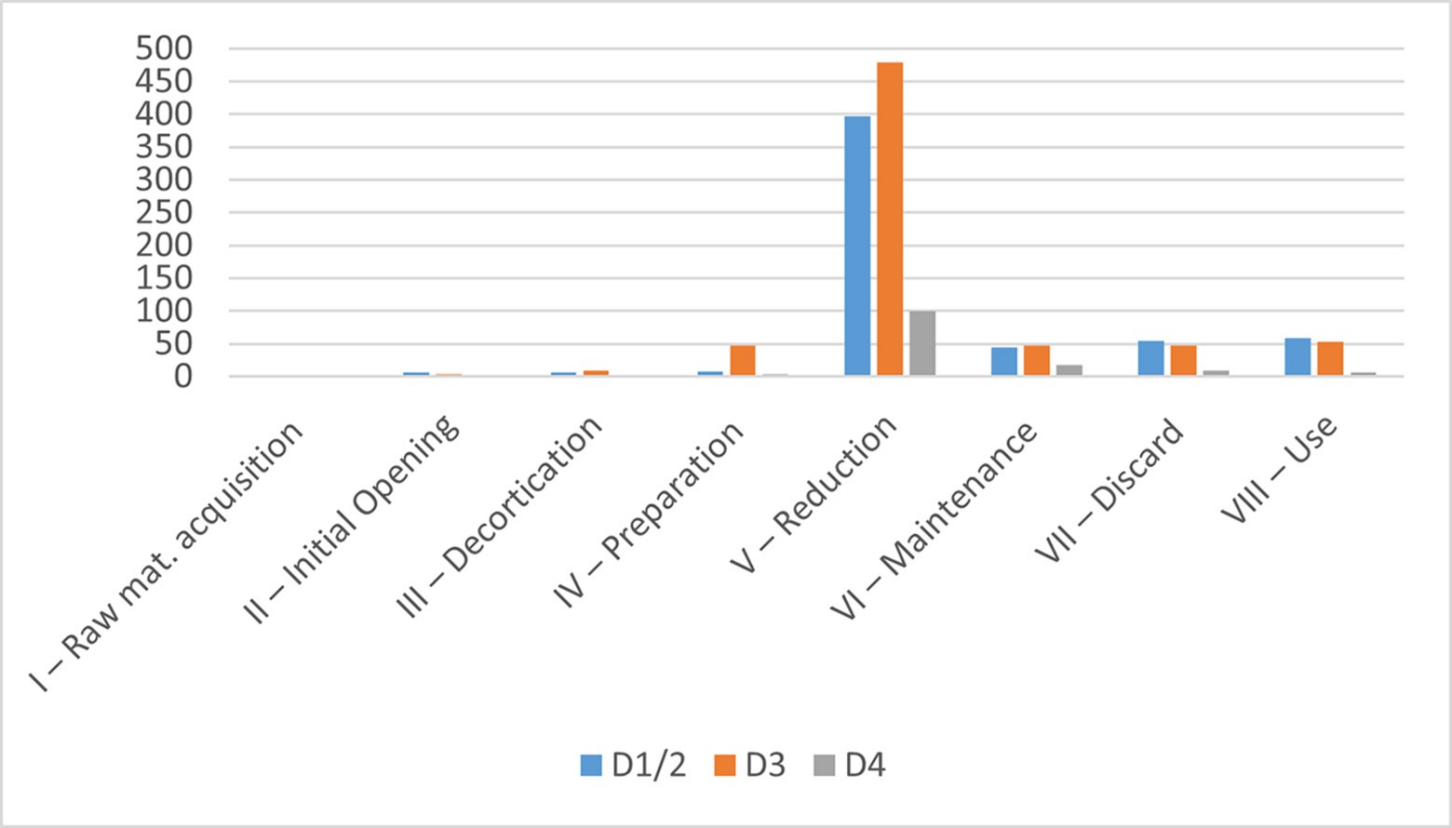
Operational chain stages.

All three sublayers show a predominance of the later stages of the reduction sequence (Fig 8 and Table 2), suggesting an import of prepared cores into the site. Sublayers D1–2 and D3 show very comparable percentages, while sublayer D4, with the smallest assemblage, shows markedly reduced reduction intensity and fewer preserved tools and cores. In sublayers D1–2 and D3 retouched bladelets are as frequent as carinated items. All three carinated assemblages show a complete representation of the reduction sequence, the similarity of the two younger assemblages suggests a comparable site function or functions of the carinated assemblages.

### Carinated ‘endscraper’ operational chains

Carinated ‘endscrapers’ are generally divided into two groups, carinated and nosed ‘endscrapers’ [41], the latter exhibiting one or two lateral notches. Both can be divided into items with a single carinated face and items with multiple carinations.

**Fig 9:**
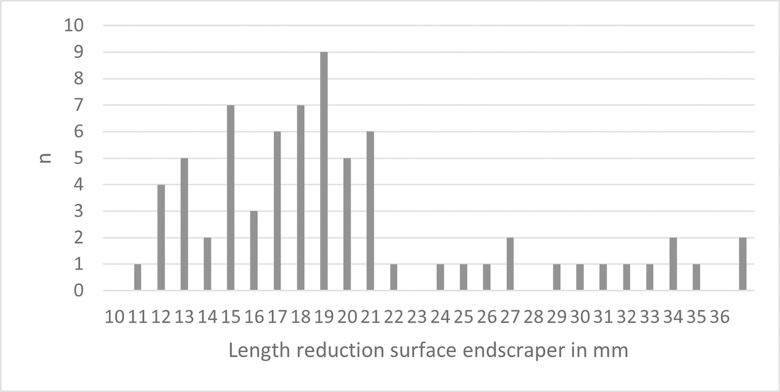
Length values of carinated ‘endscraper’ reduction surfaces.

The classical single carinated ‘endscraper’ is the most frequent type in all levels in Hayonim Cave D, both, nosed and multiple ‘endscrapers’ are quite scarce. Multiple ‘endscrapers’ only occur in the younger two levels, while nosed ‘endscrapers’ are more frequent in the lower level. The five multiple ‘endscrapers’ comprise two reduction surfaces (Table 3; e.g. Plates 4.7 and 5.8) accounting for a total of 71 reduction surfaces in the analysis (cf. Fig 9).

**Plate 4:**
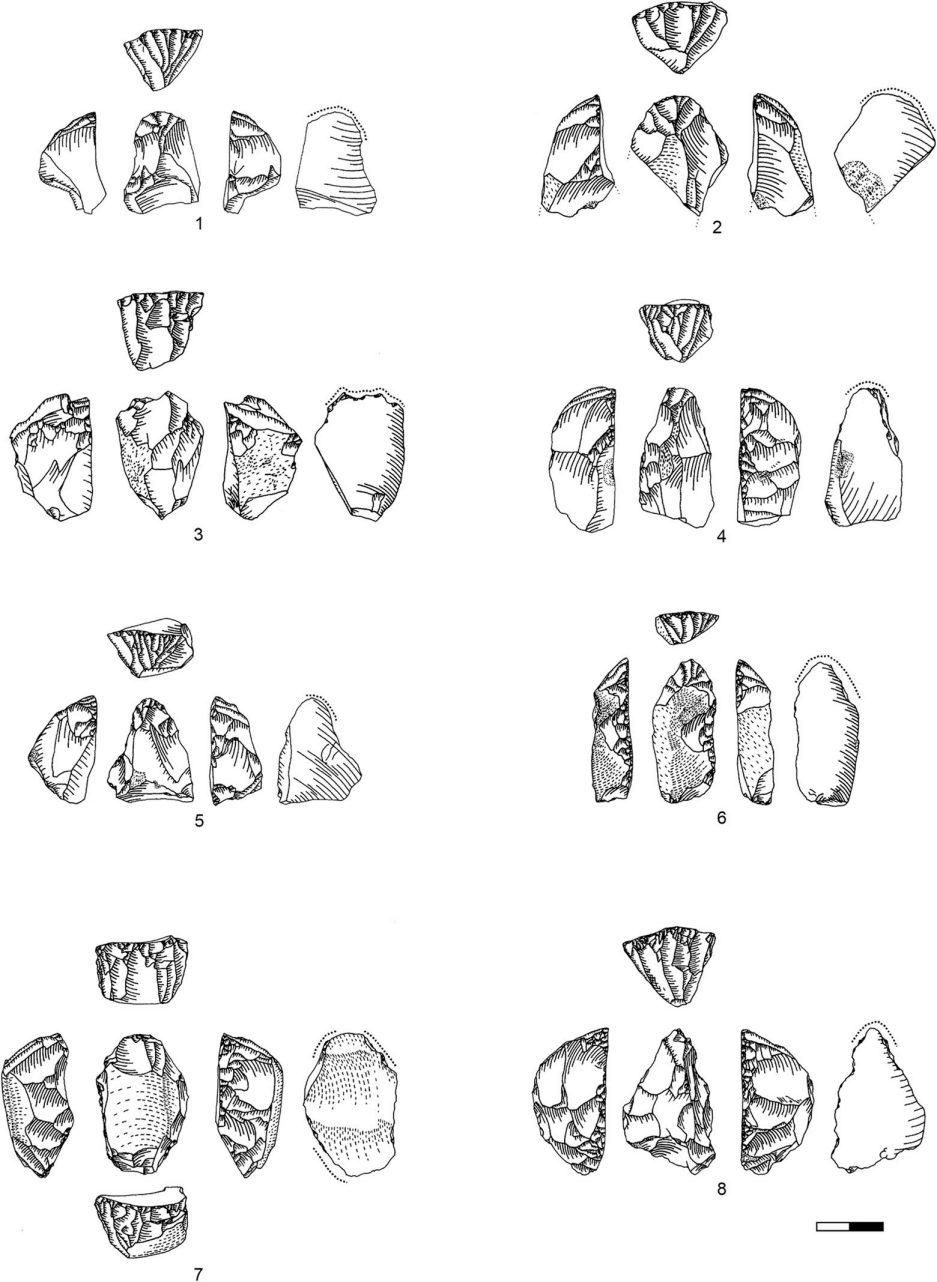
Carinated endscraper. 1–4, 7 D3; 5–6, 8 D1-2.

**Plate 5:**
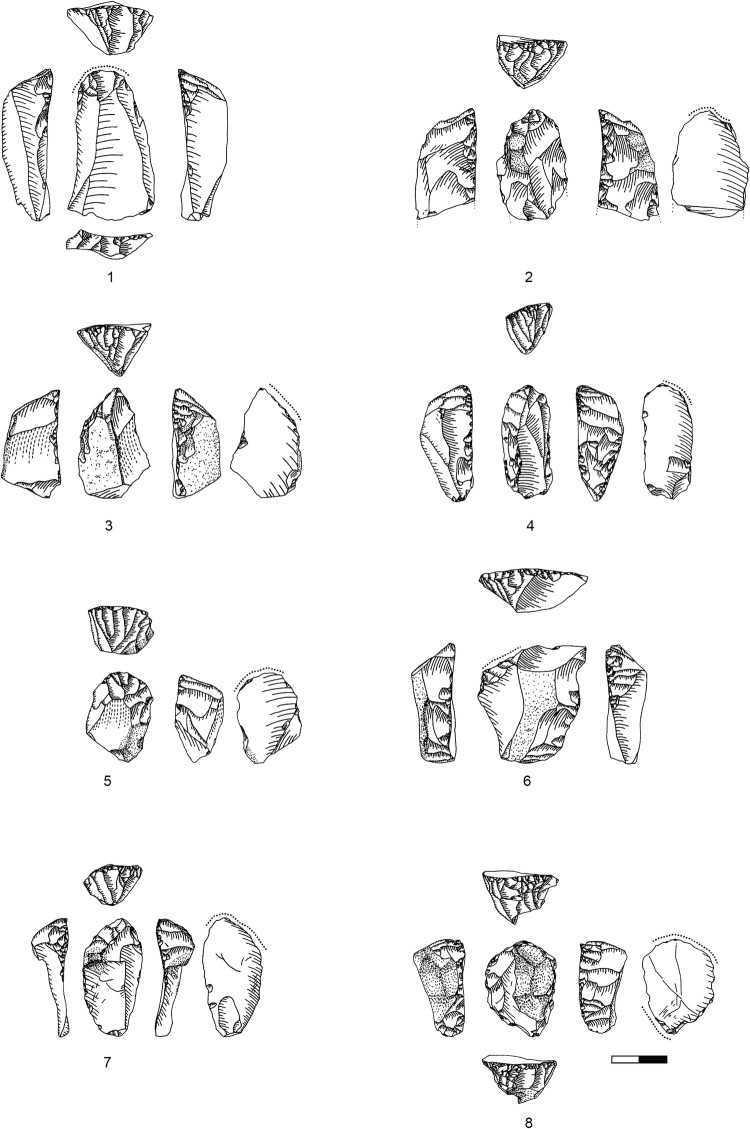
Carinated endscraper. 1–4, 6–8 D1-2; 5 D3.

**Table 3:** Hayonim Cave D—Carinated ‘endscrapers’.

		short		long		
		carinated	nosed	carinated	nosed	Total
**D1/2**	Single	21	6	2	2	31
	Multiple	3				3
**D3**	Single	17	3	3	2	25
	Multiple	1		1		2
**D4**	Single		2	2	1	5
	Multiple					0
**Total**	Single	38	11	7	5	61
	Multiple	4	0	1	0	5
	Total	42	11	8	5	66

Conceptually, there are only two different modes of ‘endscraper’ reduction. According to the length of their reduction surface, the ‘endscrapers’ can be sorted into two groups: the majority of items is between 10–20 mm long (Fig 9), while 15 ‘endscraper’ reduction surfaces are markedly larger, more than 23 mm in length.

**Plate 6:**
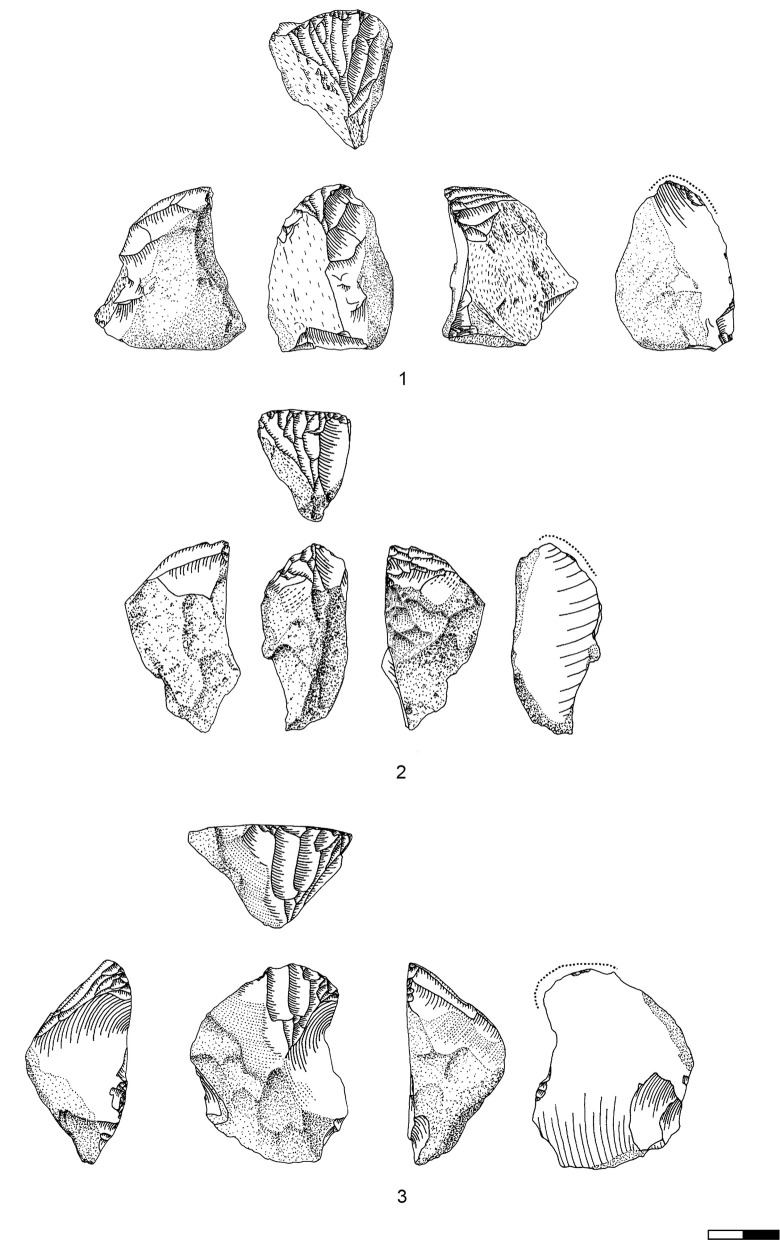
Carinated endscraper. 1 D; 2–3 D3.

**Plate 7:**
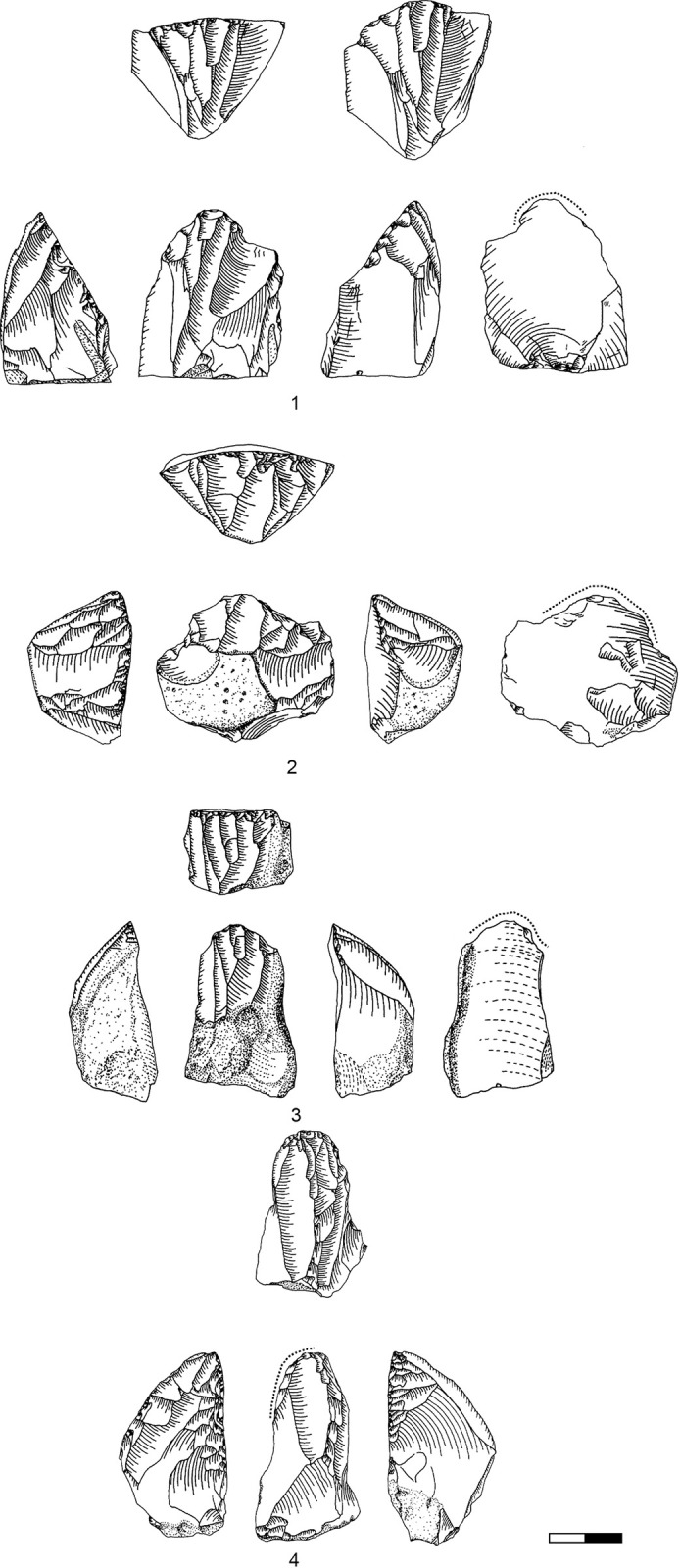
Carinated endscraper. 1–2 D4; 3 D1-2; 4 D3.

Consequently, relatively few ‘endscrapers’ are fashioned to produce large, straight bladelets from a long converging reduction surface (Fig 10.1 and Plates 6, 7). The majority shows a short reduction surface with a clear lateralised convergence, producing small, twisted, ‘comma-shaped’ bladelets (Fig 10.3 and Plates 4, 5). Both varieties occasionally portray lateral notches to address specific issues in lateral preparation/maintenance and thus fulfil the typological criterion for a nosed carinated ‘endscraper’.

**Fig 10:**
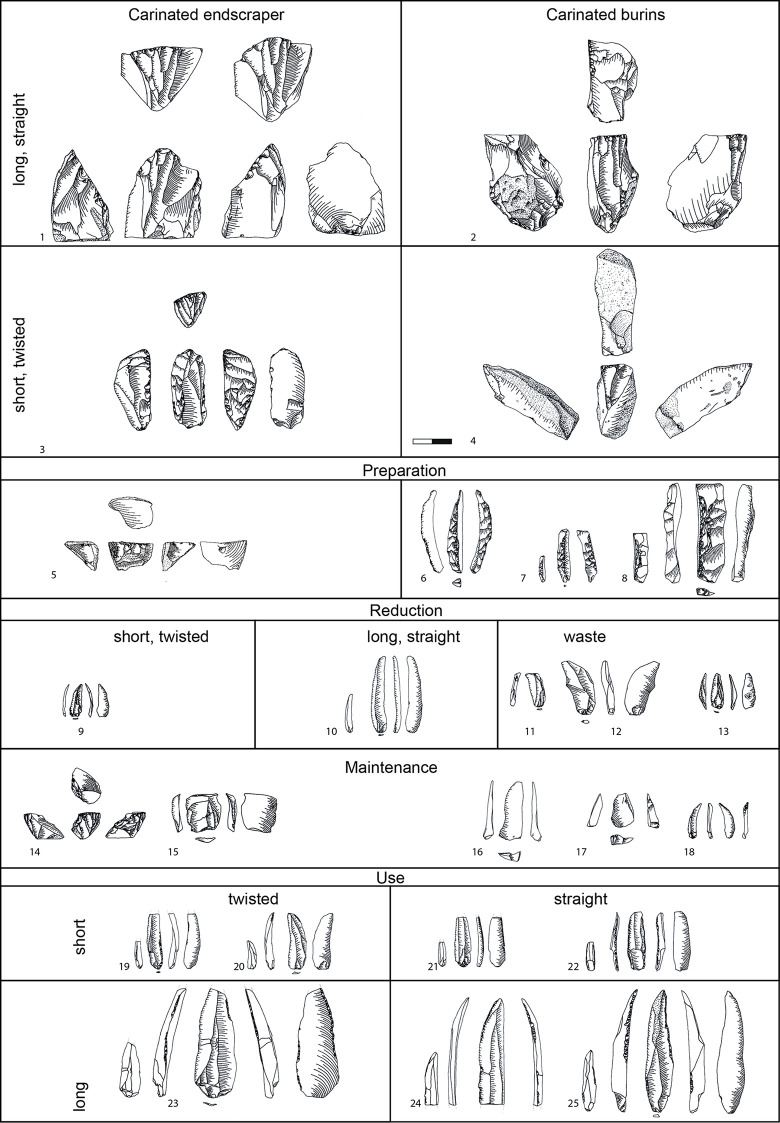
Overview of carinated products from Hayonim Cave D.

The short carinated versions are exclusively found in the younger levels (Table 5). Short nosed ‘endscrapers’ are found in all levels with decreasing frequency in the two lower levels. Long carinated and nosed ‘endscrapers’ are present throughout the sequence in low frequencies but comprise a greater share of the ‘endscraper’ assemblage in the two lower levels. A certain chronological correlation appears to be present in the data including a decrease in ‘endscraper’ size. Aside from the different conceptualisation of the reduction surface, carinated ‘endscrapers’ yielding straight and twisted products show a comparable preparation and maintenance process. The majority of carinated ‘endscrapers’ in Hayonim Cave are made on flakes or on blanks so heavily modified that their original morphology is indeterminable (Table 4).

**Table 4:** Carinated ‘endscraper’ blanks.

			blade	flake	indet. Fragment	Total
**short**	carinated	D1/2	5	12	7	24
		D3	4	5	9	18
		D4	0	0	0	0
		Total	9	17	16	42
	nosed	D1/2	1	2	3	6
		D3	0	3	0	3
		D4	0	2	0	2
		Total	1	7	3	11
**long**	carinated	D1/2	0	0	2	2
		D3	1	1	2	4
		D4	0	2	0	2
		Total	1	3	4	8
	nosed	D1/2	0	1	1	2
		D3	0	2	0	2
		D4	0	1	0	1
		Total	0	4	1	5
	Total	D1/2	6	15	13	34
		D3	5	11	11	27
		D4	0	5	0	5
		Total	11	31	24	66

**Fig 11:**
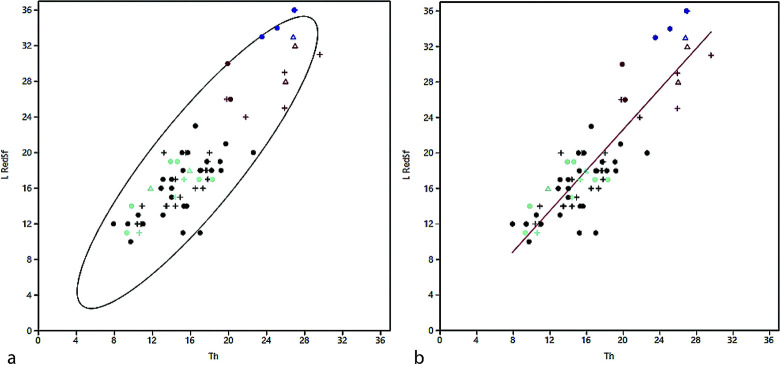
Length of the reduction surface (y) vs. endscraper thickness (x). a) scatter plot with 95% ellipse, b) linear regression, r = 0.88353, p = 2.02E-24 (black = short carinated, turquoise = short nosed, red = long carinated, dark blue = long nosed, dot = D1/2, plus = D3, triangle = D4).

‘Endscraper’ blank selection seems to have been focused mainly on the thickness of the blank, which is mostly used in its entire available length. A plot of the ‘endscraper’ thickness against the length of the reduction surfaces shows a clear correlation (Fig 11A) and a t-test confirms the statistical equality of the two samples (t = -2.2356; p = 0.026964) as does a simple linear regression (r = 0.88353, p = 2.02e-24; Fig 11B). Nosed ‘endscrapers’ are among the larger specimens in the sample, while on the whole, the ‘endscrapers’ of level D4 are among the largest carinated ‘endscrapers’ in the studied sample.

Core initiation requires a lateral and frontal preparation including the optional establishment of a lateral notch in blanks not sufficiently lateralised in their primary shape. Most ‘endscrapers’ are designed for a right-sided lateralisation (Table 1 in S1 File; most prominently: Plates 4.1,5,6 and 5.3–7). The short carinated and nosed ‘endscrapers’ especially show a predominance of a lateralised concept, while the large carinated items are predominantly straight. The short ‘endscrapers’ are predominantly right-sided, which might potentially relate to the handedness of the knapper, as a right-sided ‘endscraper’ allows reduction with a hammerstone in the right hand. The reduction surfaces are predominantly converging, making use of the dorsal ridge of the ‘endscraper’ (as a blank) (Table 2 in S1 File) and together with the lateralised configuration ensuring the twisting of the target products.

The reduction surface of most ‘endscrapers’ is located at the distal end and only rarely at the proximal part of the blank (Table 3 in S1 File). In all multiple ‘endscrapers’ there is an opposed configuration of one proximal and one distal carination (Plate 5.8).

**Fig 12:**
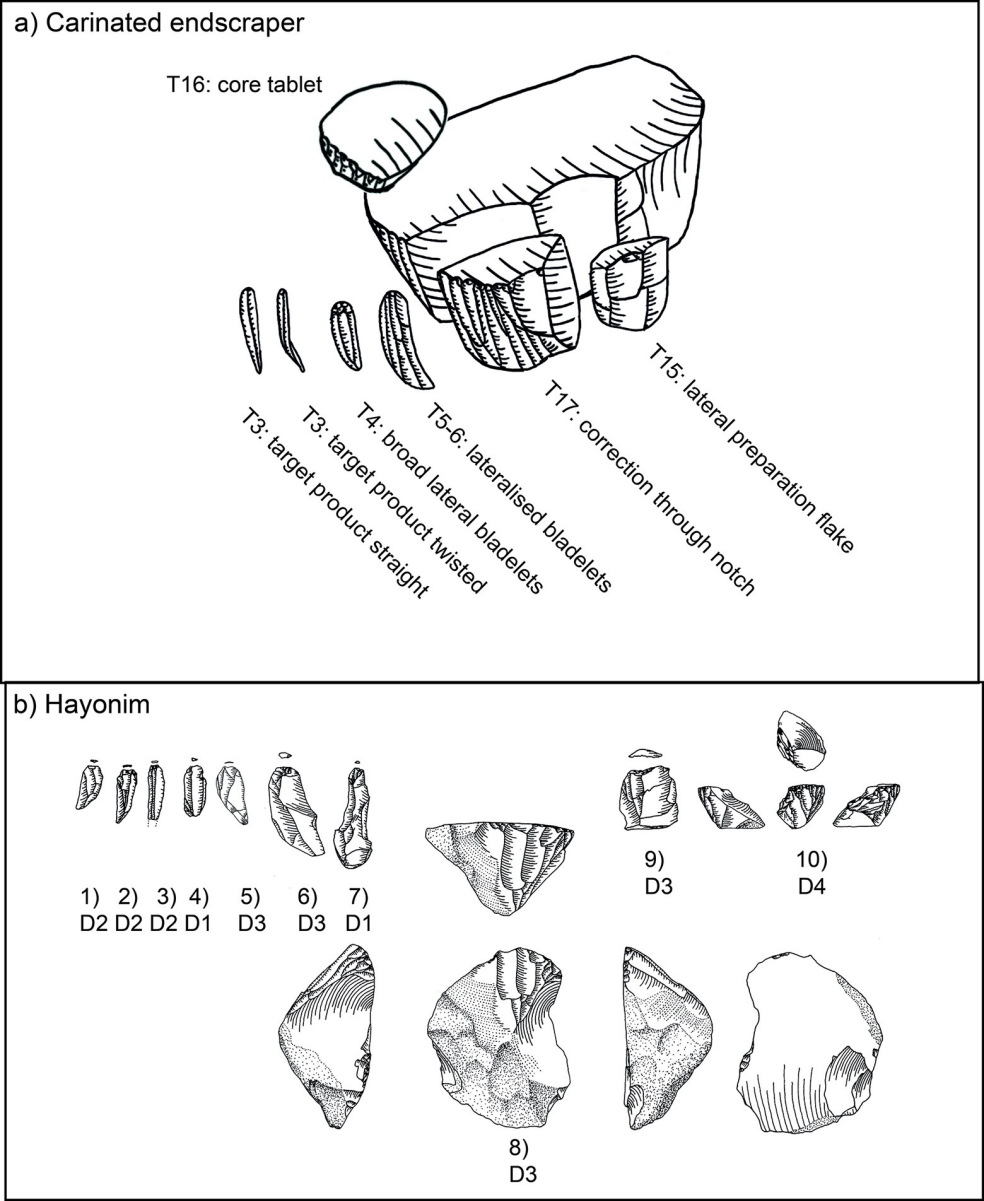
Operational chain of carinated ‘endscraper’ reduction. Schematic representation (top, after [51]) and evidence from Hayonim Cave (bottom).

**Plate 8:**
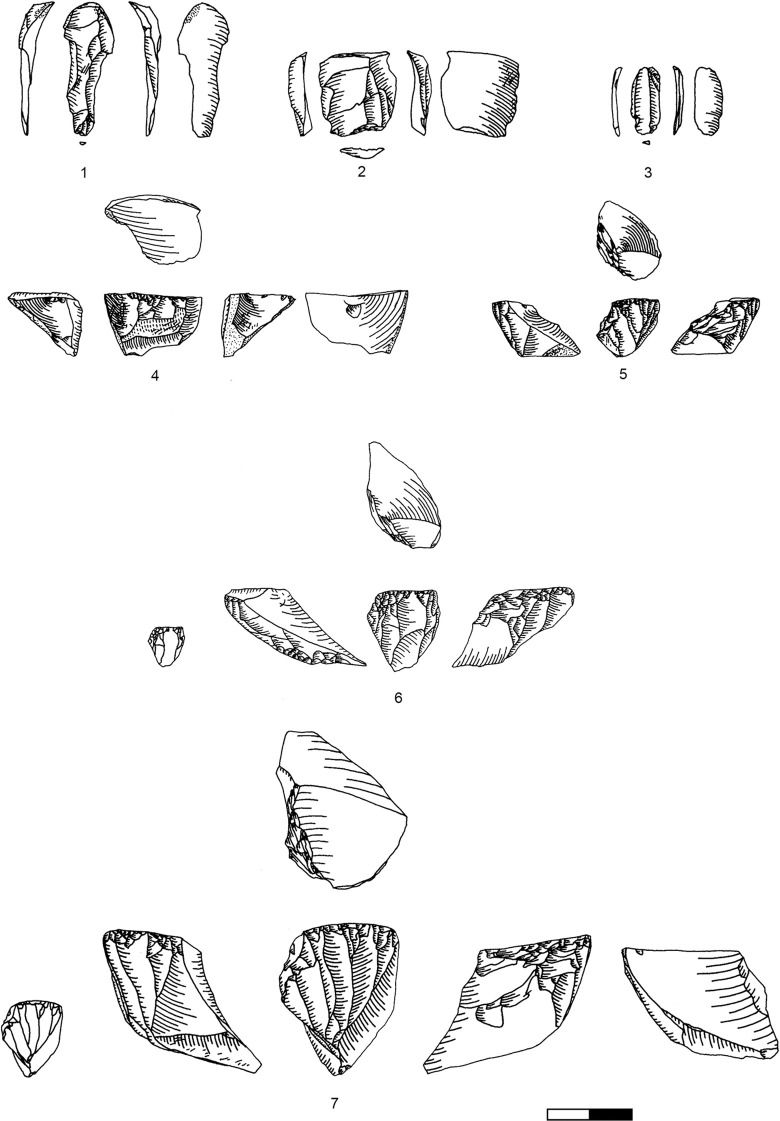
Core trimming elements. 1, 3 D1-2, 2, 7 D3, 4–6 D4.

Core preparation focuses on the lateral parts of the core blank, thus nearly all ‘endscrapers’ show some form of lateral investment (Table 4 in S1 File; present: e.g. Plates 6.3; 7.1–4; 4.1,4–8; 5.2,4–5, 8; absent: e.g. Plates 6.1–2; 4.2–3; 5.1,3,6, 7). Lateral preparation originates from the ventral surface of the blank, hence the platform of the core, and targets the establishment of lateral convexities through one or several removals (Fig 12: T7, b9) resulting in more (e.g. Plates 6.3; 7.1; 4.1) or less (e.g. Plates 7.3–4; 4.2, 4–5, 8) pronounced notches. Further preparation is rarely required, and only very few ‘endscrapers’ show an alteration of the platform (e.g. Plate 7.1, Fig 12A T16).

**Fig 13:**
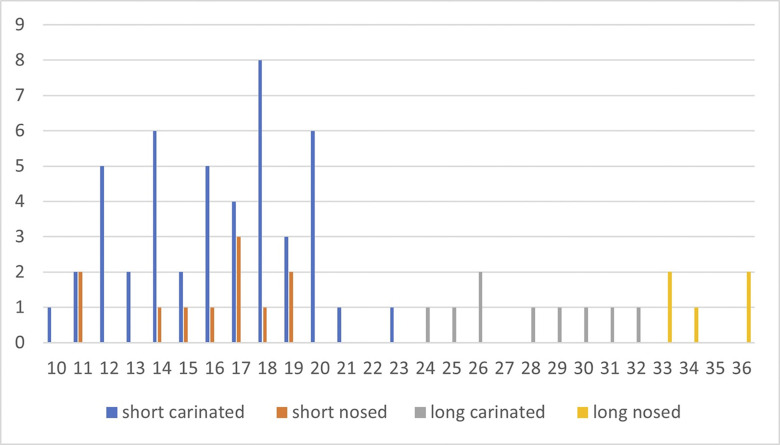
Length of the reduction surface.

After only little investment in preparation, reduction can start (Fig 12AT3; b1–3). The ‘endscrapers’ for the production of twisted products supposedly yield a longer *series* of bladelets [80], while the ones for straight products are assumed to yield an *assortment* of central target products and lateralised waste products. A *series* of blanks is understood as the result of a continuous reduction without the need of intermittent maintenance operations, while frequent maintenance operations result in an *assortment* of different blanks in addition to the intended target products. Neither carinated system in Hayonim Cave D results in a true series of target products without any necessity for intermittent maintenance operations. Reduction surface lengths differ between the four different types of carinated ‘endscrapers’, short and long ones and standard and nosed ones (Fig 13). Nosed ‘endscrapers’ are generally among those with a longer reduction surface. The creation of a lateral notch is an optional preparation or maintenance activity, potentially reserved for larger cores allowing for a lateral removal of substantial parts of the reduction surface without wasting the core.

Maintenance activities are mainly required to re-establish the twist of the reduction surface, re-establish lateral convexities and correct hinge fractures occurring during reduction. To these ends large parts of the reduction surface (cf. [51]) are struck off, creating a new notch at the lateral side of the reduction surface (Fig 12A T17, b10; Plate 8.4–7). These maintenance products preserve large series of hinges (Plate 8.5–7) clearly indicating the necessity of the upkeep operation. One item (Plate 8.4) shows remains of a patinated old surface on the reduction surface, indicating a removal in the earliest stages of core preparation. Few lateral corrective products are recognisable in the assemblage (Fig 12A T4–6; b 4–7; Plate 8.1–3), mainly consisting of overshot bladelets from the lateral portions of the ‘endscraper’. Only very rarely a correction of the platform was observed, resulting in a small flake with remains of the reduction surface on the platform and remains of the ‘endscraper’s’ ventral surface on its dorsal side (Fig 12A T16). Only one such flake could be identified in the assemblage of Hayonim Cave D.

Core abandonment is mainly related to the occurrence of hinged knapping accidents during reduction (e.g. Plates 4.3,7–8; 5.2,7–8) and more rarely, to the exhaustion of the blank. Nearly half of the ‘endscrapers’ show a hinge fracture of the last negative (Table 5 in S1 File).

One of the indications for a core function of carinated items is the abrasion at the last removal negative [51]. An integral part of reduction, abrasion of the platform edge has the function of removing little ridges left by the previous removal of bladelets. It is also one of the last steps of the preparation of a functional endscraper for hide-working, as these little ridges would cut into the hide and hinder an even removal of fat and other tissue from the inside of the hide (ethnographic information on hide-working practices see: [81, 82], for a view of the functional end [81], Fig 4.4). If this abrasion is thus missing at the last negatives, which is the case in more than half of the carinated ‘endscrapers’ in Hayonim Cave (Table 5), neither a resuming of the reduction nor use as a tool was apparently intended.

**Table 5:** Abrasion of the last negative.

			absent	present	Indet.	total
**short**	carinated	D1/2	14	9	4	27
		D3	6	13		19
		D4				0
		Total	20	22	4	46
	nosed	D1/2	3	3		6
		D3	1	2		3
		D4	1		1	2
		Total	5	5	1	11
**long**	carinated	D1/2	2			2
		D3	5			5
		D4	2			2
		Total	9	0	0	9
	nosed	D1/2	2			2
		D3		1	1	2
		D4	1			1
		Total	3	1	1	5
	Total	D1/2	21	12	4	37
		D3	12	16	1	29
		D4	4	0	1	5
		Total	37	28	6	71

A previous study of use wear on the Hayonim Cave tools [83] confirmed one large non-carinated endscraper (Plate 9.6) to have been used for the scraping of wet and dry hide. It clearly shows another indication usable in the distinction between tools and cores, which is the shape of the platform edge or the working edge of the tool. The endscraper for hide working shows a very smoothly rounded edge without any protruding ridges, spikes or other irregularities that could damage the hide to be worked. If this edge is compared to some of the carinated ‘endscrapers’ (e.g. Plate 4.3,8) the unsuitability of the latter for a hide-working task is easily apparent. Not all the carinated ‘endscrapers’ of Hayonim Cave would thus qualify for use as a tool in their final state. It is, however, possible that some of those artefacts were usable as tools earlier in their life history, a matter which cannot be excluded without a use-wear study on endscrapers and their reduction products.

**Plate 9:**
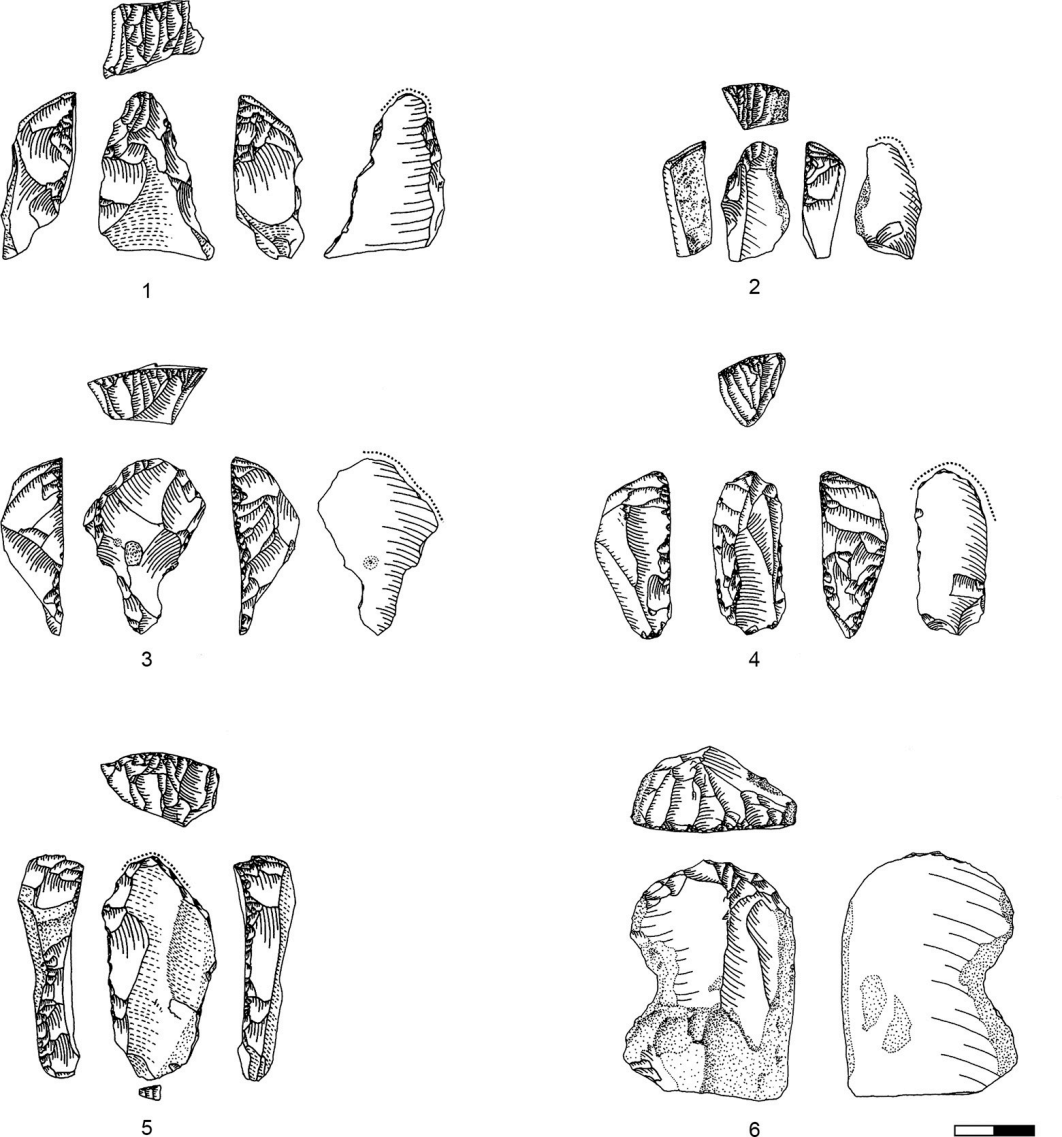
Carinated endscraper and regular endscraper. 1–5 Carinated endscraper; 6 regular endscraper used for working wet and dry hide. 1–2, 4–5 D3; 3, 6 D1-2.

### Carinated ‘burin’ operational chains

Carinated ‘burin’ production in Hayonim Cave D is overall less intensive with only 45 specimens analysed in the sample, 41 of which are single and four of which are multiple with up to three reduction surfaces, giving a total of 51 reduction surfaces. 36 items are standard carinated ‘burins’ [51], and five are of the flat faced variety, three occurring in sublayer D3 (Table 6). Two of the multiple specimens (Table 7) show opposing reduction surfaces, one proximal and distal and the other left and right laterally. The other two have three reduction surfaces, one with a proximal-distal-left lateral configuration and one with one distal and two off-angle/transverse located reduction surfaces.

**Table 6:** Carinated burin types.

	D1/2	D3	D4	Total
**Carinated burin**	17	17	2	36
**flat faced CB**	1	3	1	5
**Multiple CB**	2	1	1	4
**Total**	20	21	4	45

**Table 7:** Configuration of multiple reduction surfaces in carinated burins.

	D1/2	D3	D4	Total
**single**	18	20	3	41
**opposed**	1	1		2
**rotated**	1		1	2
**Total**	20	21	4	45

**Fig 14:**
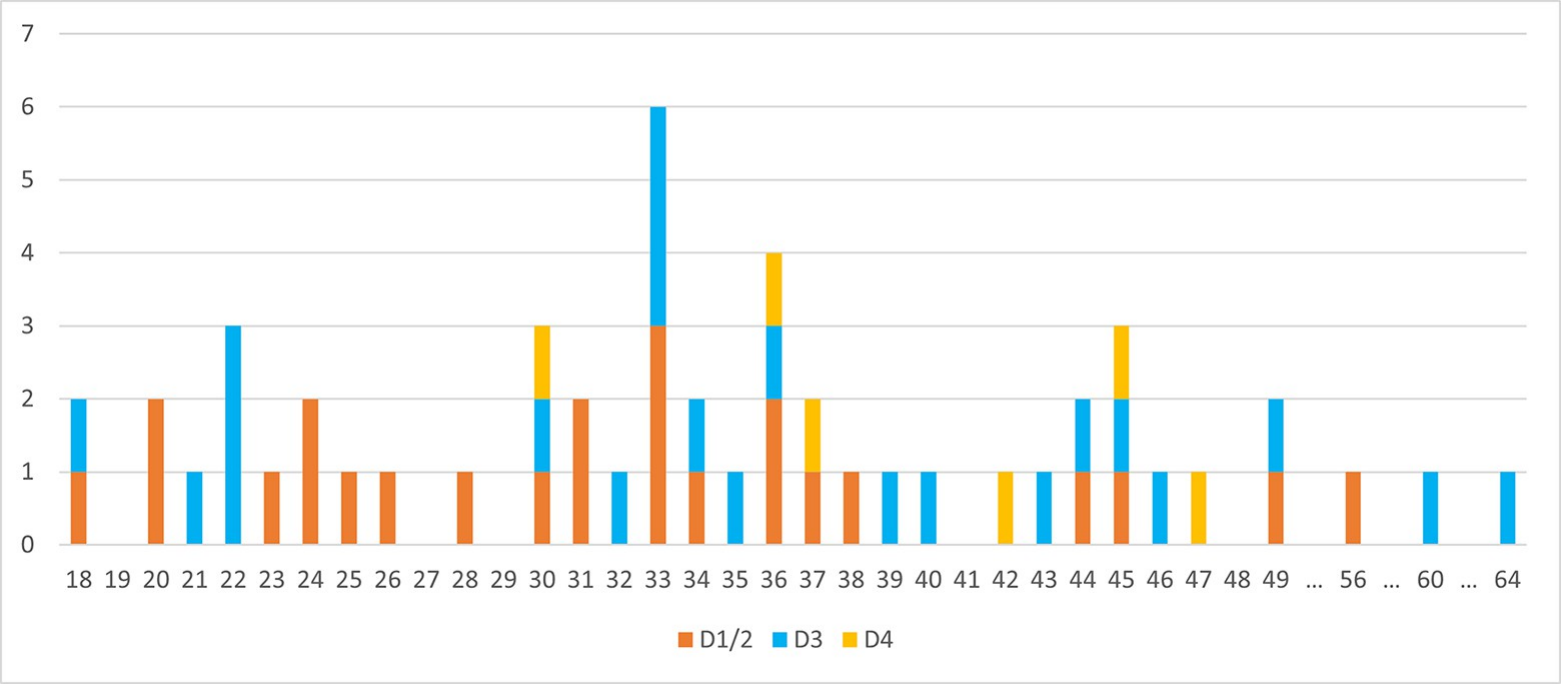
Carinated ‘burins’. Length of reduction surface.

The length of the reduction surface of carinated ‘burins’ suggests a division into three length groups, which are, however, less distinct than those of the carinated ‘endscrapers’ (Fig 14). Generally, reduction surfaces range between 18 mm and 64 mm in length with the majority being between 30 mm to 40 mm.

Flake blanks are preferentially selected for the shaping of carinated ‘burins’ (Table 6 in S1 File). The carination is applied mostly to the distal and, less often, to the proximal end of the blank; lateral or off angle carination is relatively rare (Table 7 in S1 File).

**Fig 15:**
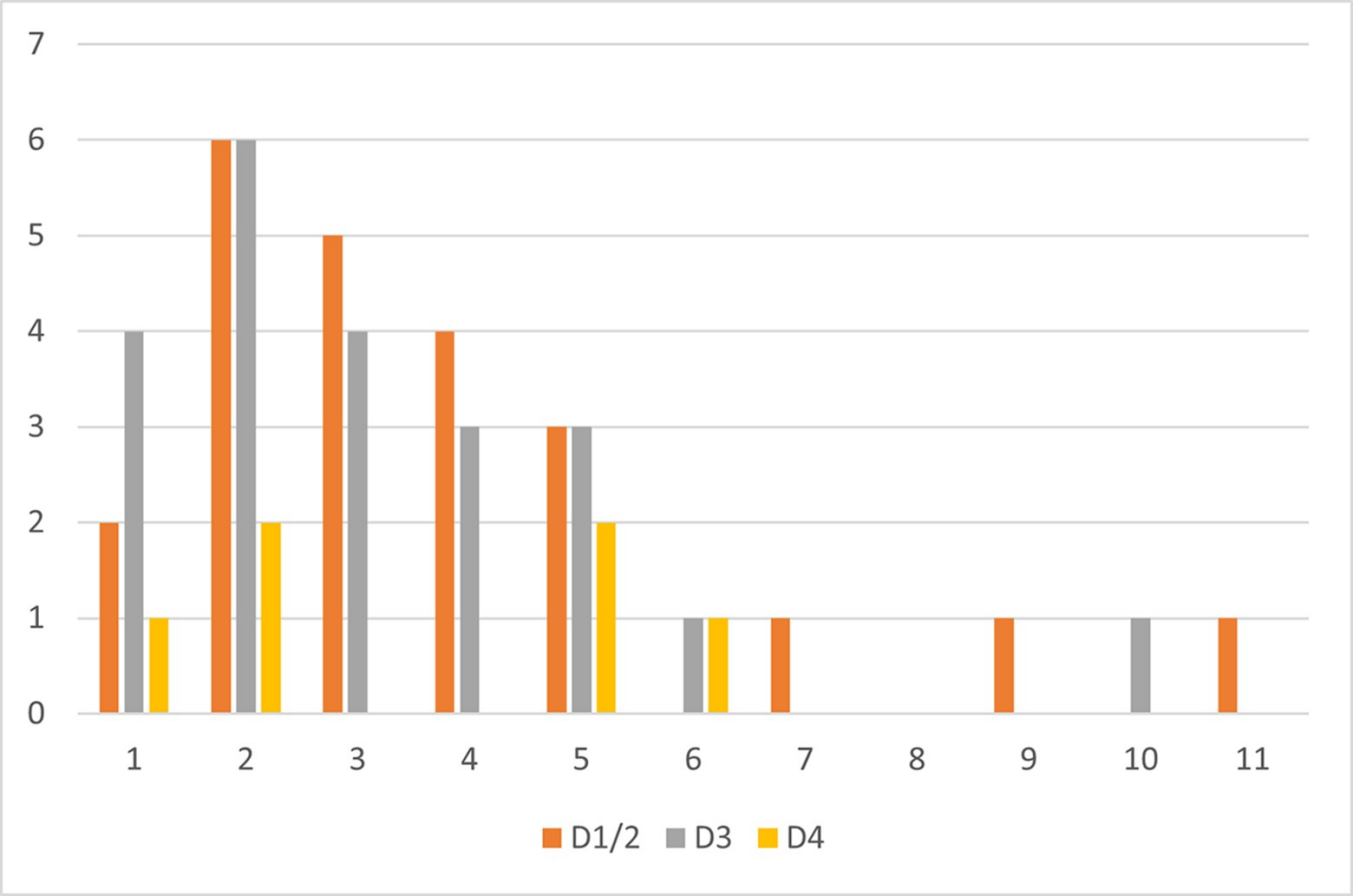
Number of reduction negatives on carinated ‘burins’.

**Fig 16:**
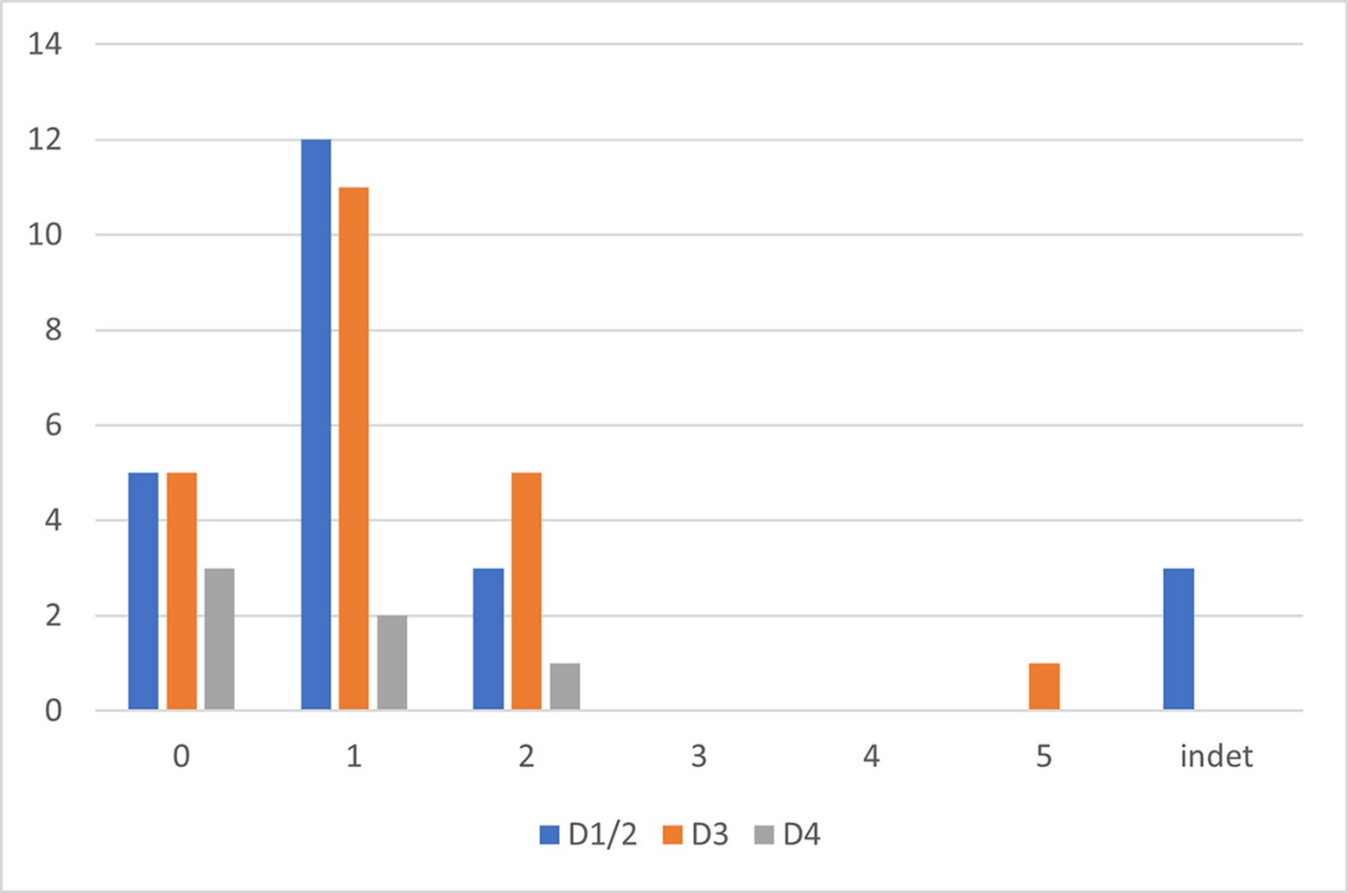
Number of core tablets on carinated ‘burins’.

**Fig 17:**
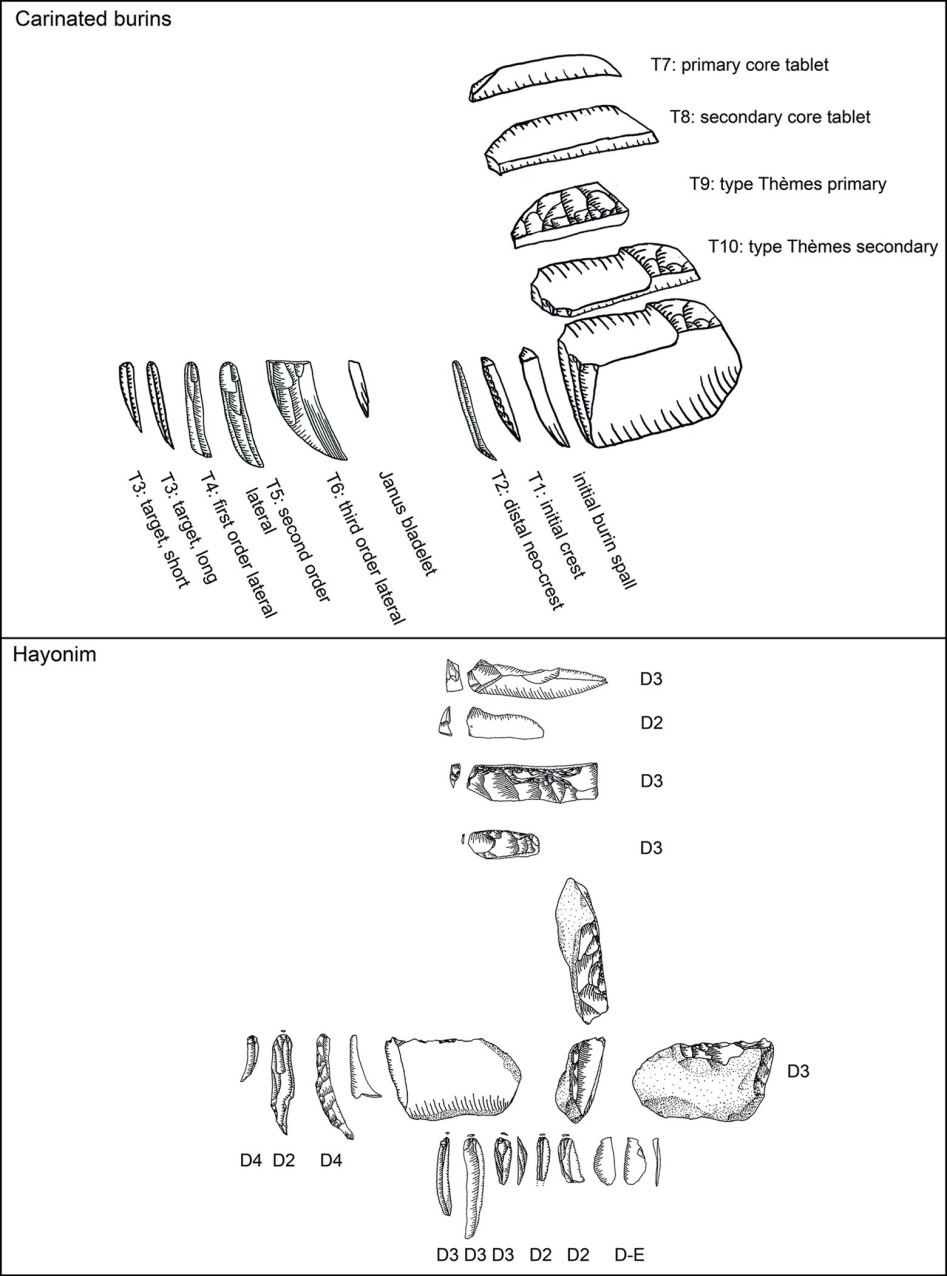
Operational chain of carinated ‘burin’ reduction. Schematic representation (top, after [51) and evidence from Hayonim Cave (bottom).

Carinated ‘burin’ preparation is more complex than that of carinated ‘endscrapers’ and involves multiple parts of the blank. Generally, a differentiation can be made between reduction surface and platform preparation, based both, on the general setup of the preparation and of the imbalance between reduction intensity of the two angled faces. Negatives on the reduction surface are more numerous (Fig 15) than the preparation removals on the designated platforms (Fig 16). Preparation is commonly observed on the platform and the distal portion of the reduction surface (Table 8 in S1 File and Fig 17). Rarely, the back of the core, opposing the reduction surface, bears traces of shaping. Initial preparation focuses on the establishment of the angle between both faces and the convexity architecture. Initial platform preparation is conducted by lateral faceting, mostly from the ventral side of the blank (Table 9 in S1 File). This faceted part is then removed by a burin blow from the designated reduction surface resulting in an initial Thèmes bladelet (Fig 17A T9; Plate 10.1–5). This can (Plate 10.1) but does not have to (Plate 10.2–4), portray the former platform edge including the proximal/bulbar parts of removal negatives from the reduction surface. The preservation of these depends on two factors: the size of the platform of the Thèmes bladelet and the timing of the striking off of this bladelet in relation to reduction activities on the reduction surface. Initial full faceting is rarely preserved on the artefacts (Plates 11.4; 2.2), though remains of it can be observed on several specimens (Plates 12.1–2; 11.1). Such initial platform faceting is, however, not always mandatory and both ridged core tablets as well as those with a natural crest, can replace it during preparation and maintenance of the item (Plate 3.1–8). That these core tablets came off carinated ‘burins’ is easily verifiable by the presence of a ventral face on one lateral side of the core tablet, indicating they derive from a flake.

**Plate 10:**
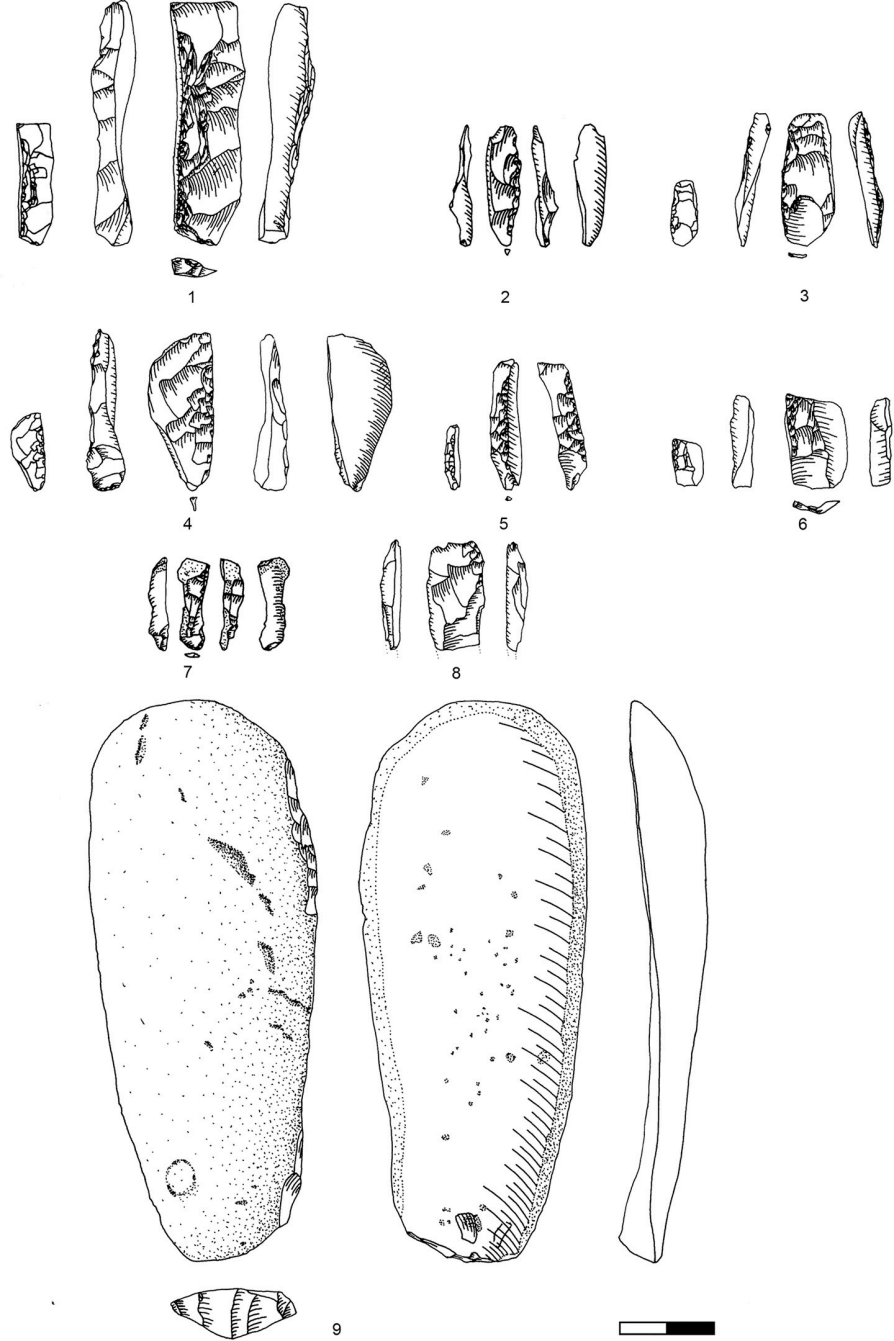
Core trimming elements. 1–3, 7–8 D3; 4, 9 D1-2, 5–6 D4.

**Plate 11:**
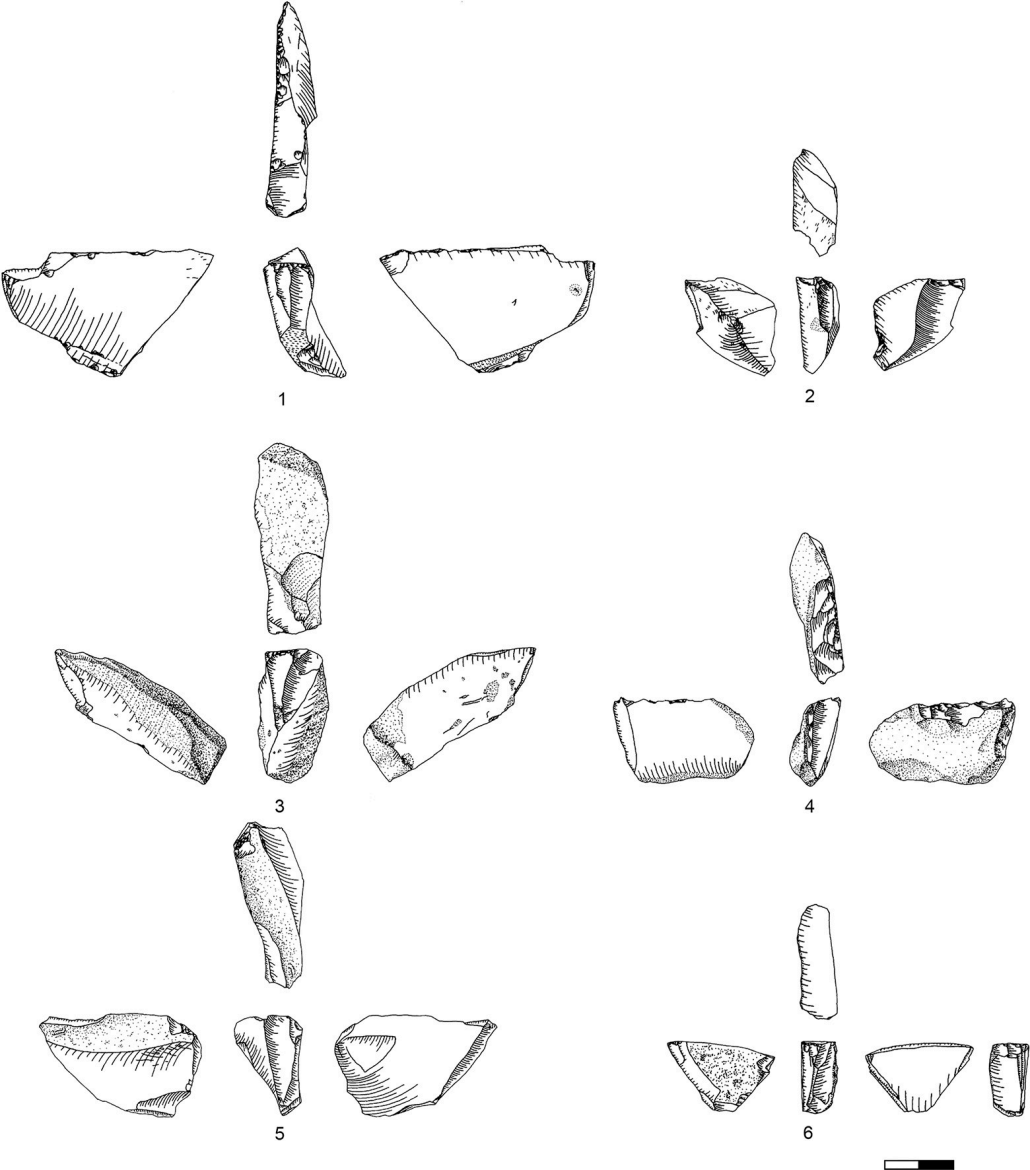
Carinated burins. 1–2, 4 D3; 3, 6 D1-2; 5 D4.

**Plate 12:**
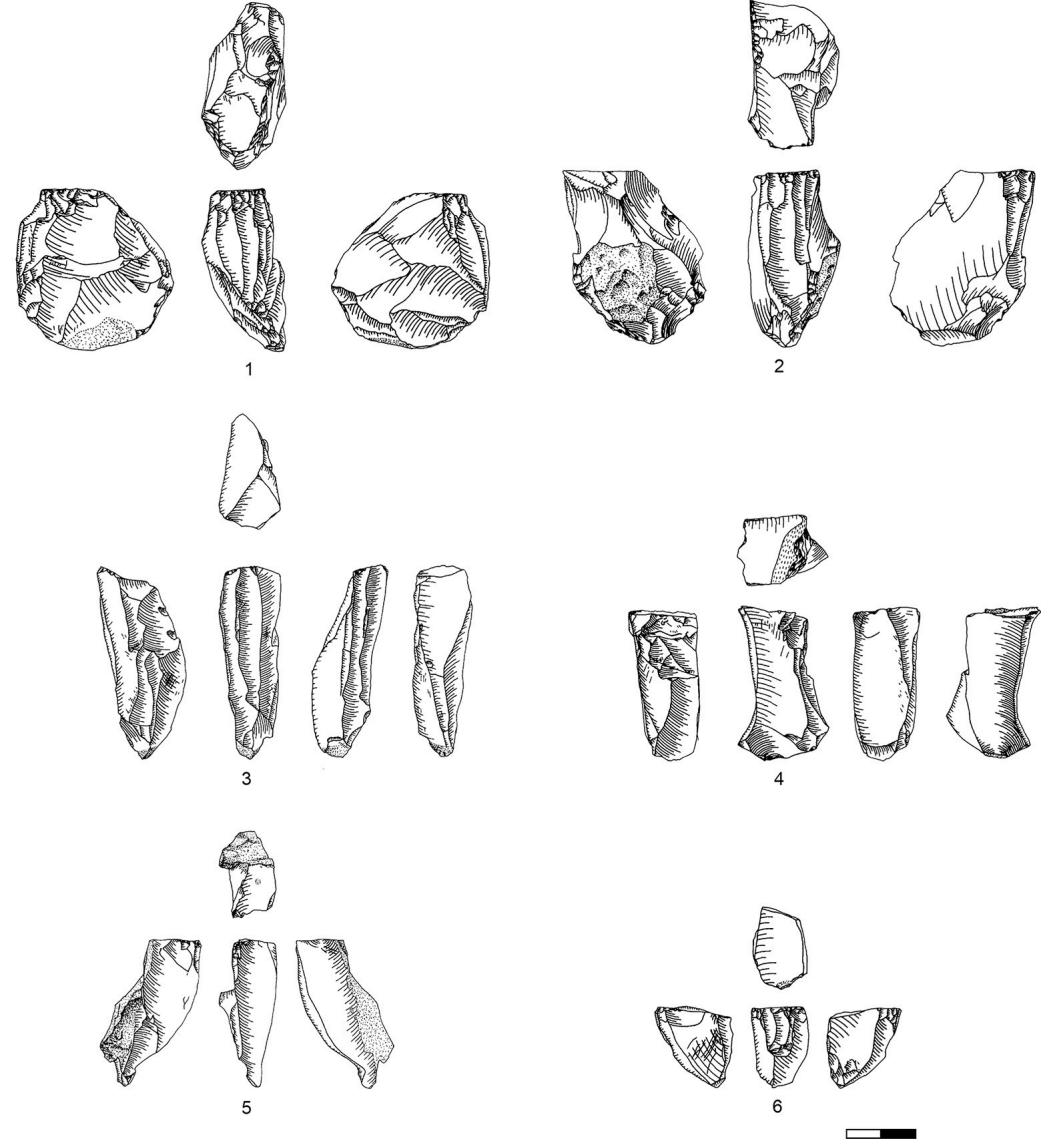
Carinated burins. 1–2, 6 D1-2; 3 D3, 4–5 D4.

Preparation of the reduction surface focuses on the establishment of lateral convergence, again through lateral faceting, mostly from the ventral face of the blank. Such retouch happens after the detachment of the original flake, and is clearly a part of the modifying actions most likely in the context of ‘burin’/core preparation. It is impossible to distinguish between preparation and maintenance actions on the discarded specimens, yet many of these bear traces of such lateral faceting (Plates 12.1–3; 11.1, 3–6; 2.2). Similar traces can be found with regard to back preparation (Plates 12.2; 2.1–3). Initial ‘opening’ of the reduction surface can result in two easily recognizable types of products: a fully crested bladelet (Fig 17AT1) which removes the remains of the initial preparation (Plate 13.4–5), and a so-called Janus-bladelet with two ventral faces: one of the bladelet itself, and the other part of the ventral face of the carinated ‘burin’ (Plate 13.1–2). Additionally, there occur initial ‘burin’ spalls recognized by their trapezoidal cross section (Plate 13.3).

**Plate 13:**
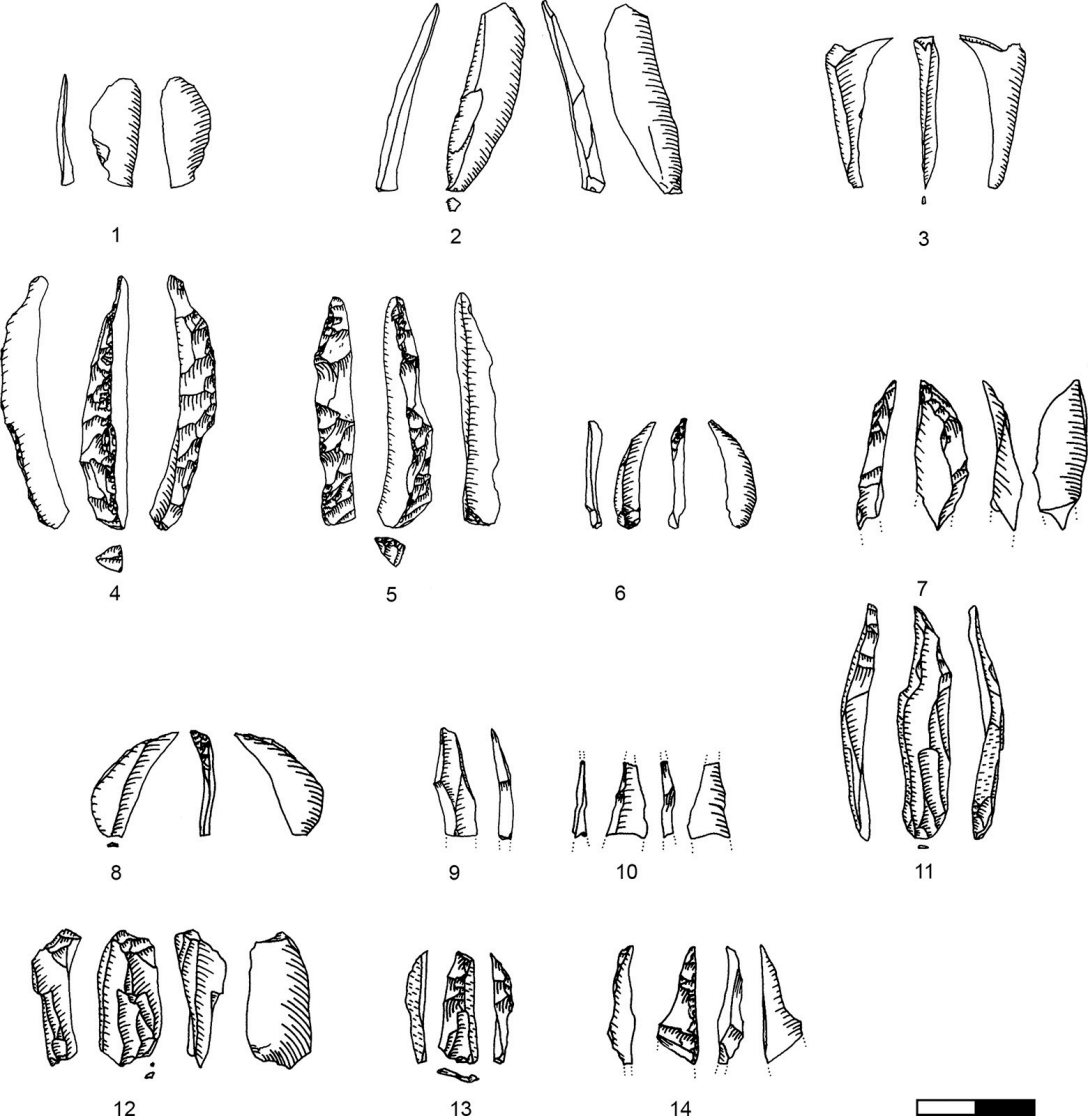
Core trimming elements. 1, 14 D; 2, 12–13 D3; 3, 7–11 D1-2; 4–6 D4.

Reduction of carinated ‘burins’ in Hayonim Cave focuses mainly on the acquisition of either straight or ventrally right-sided bladelets (Table 10 in S1 File) from converging or subparallel reduction surfaces (Table 11 in S1 File).

Maintenance actions duplicate the preparation ones to result in either partial Thèmes bladelets (Plate 10.3,6,8) or plain core tablets with the negative of a previous tablet removal (Fig 17A T8, Plate 3.9–13). Reestablishment of the distal convexity is done mainly through a distal neo-cresting, resulting in primary (Plate 13.6–8) and secondary (Plate 13.9–11) neo-cresting bladelets (Fig 17A T2).

**Fig 18:**
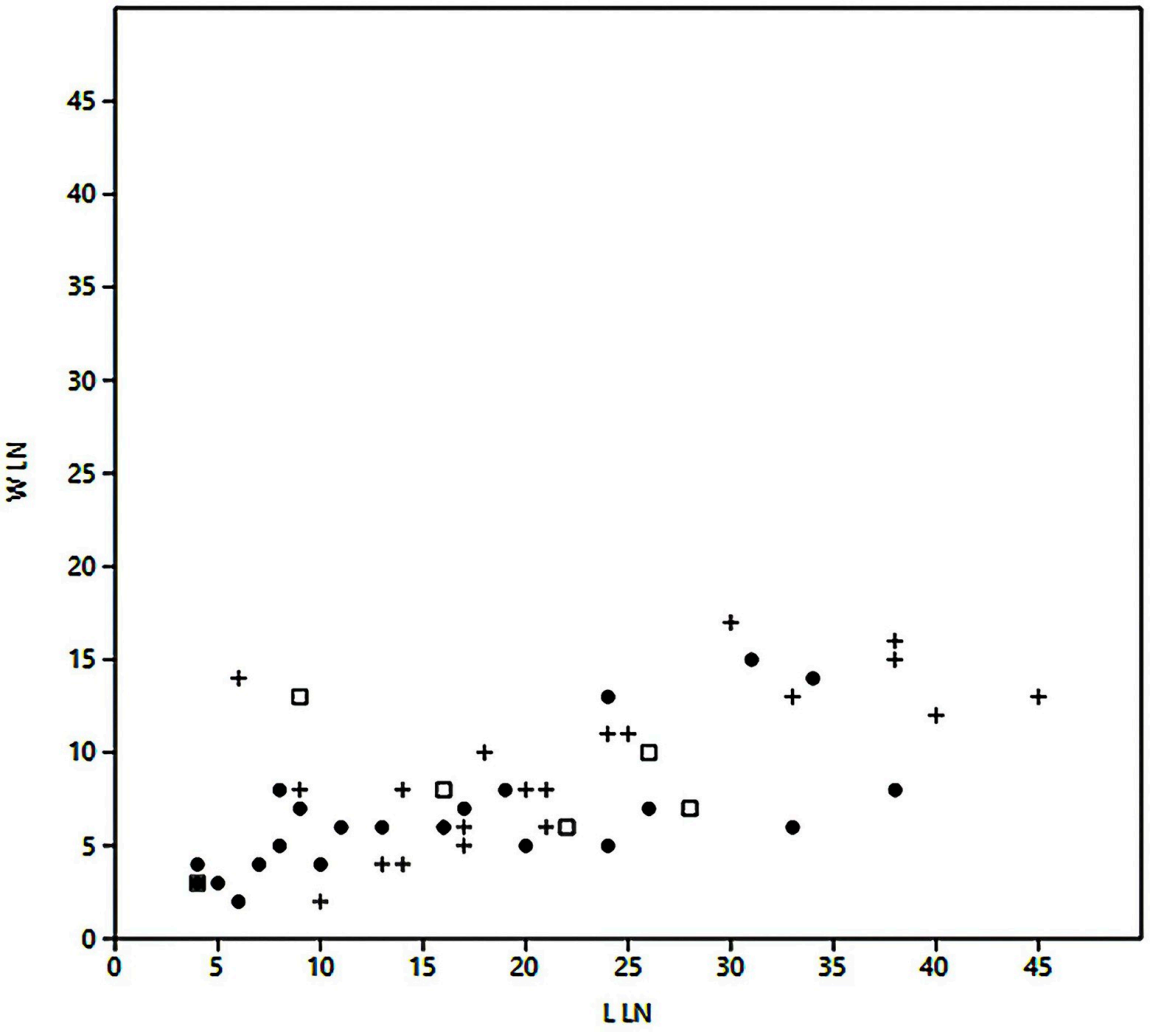
Length and width of the last negative on carinated ‘burins’. Dot = D1/2, + = D3, Square = D4.

**Fig 19:**
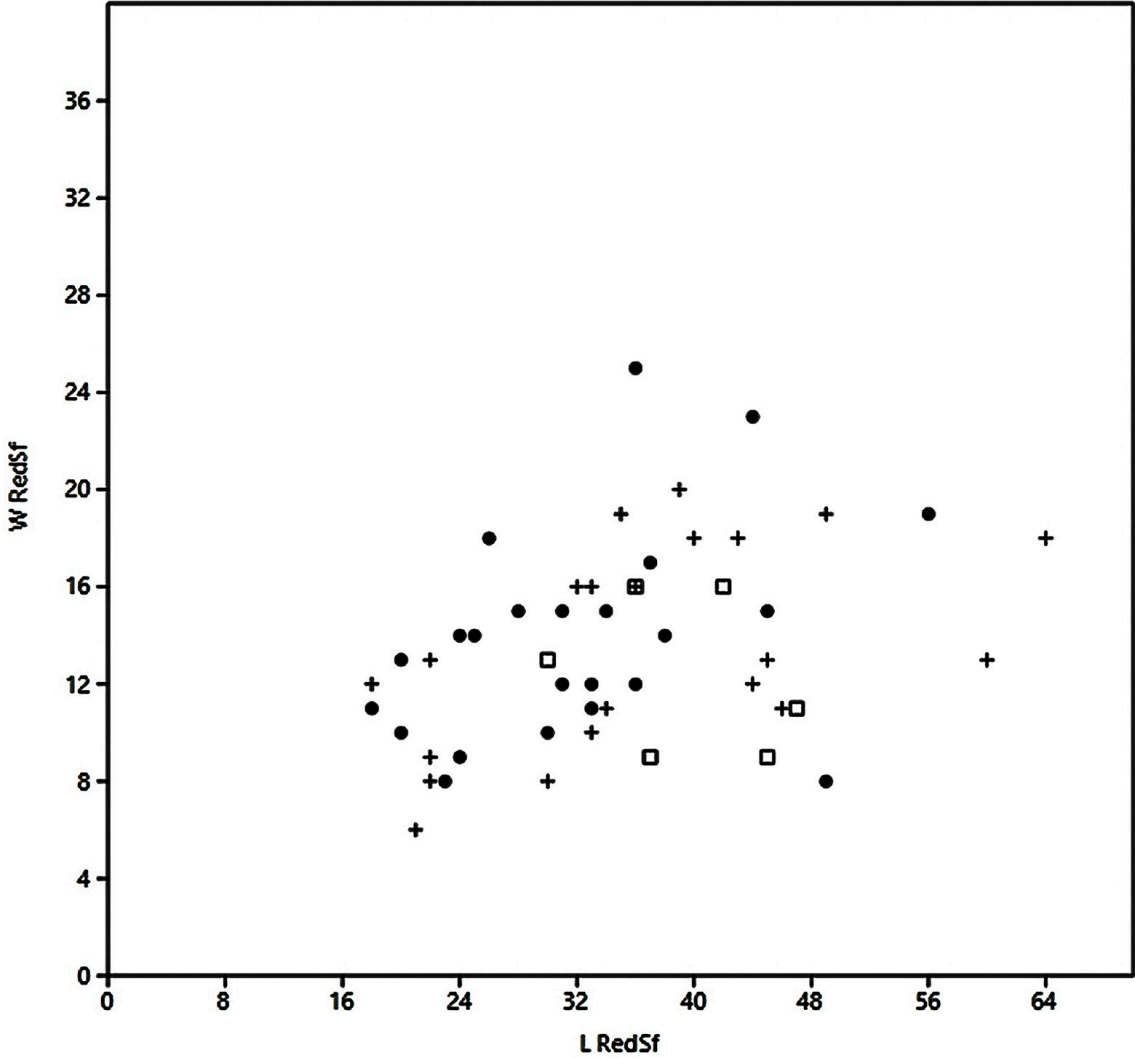
Length and width of reduction surfaces on carinated ‘burins’. Dot = D1/2, + = D3, Square = D4.

Half of the carinated ‘burins’ show hinged fractures at the last negative (Table 12 in S1 File), pointing towards likely reasons for abandonment. Last negatives reach a length of 5 to 42mm (Fig 18 and S4 File), thus being shorter than the available length of the reduction surfaces (Fig 19).

Abrasion of the last negative is mostly absent (Table 8), yet the indication of a potential tool use is less definite than for carinated ‘endscrapers’. While ‘endscrapers’ with an un-abraded edge are doubtful to be used as a tool, a burin function is unlikely to be diminished by the absence of edge abrasion.

**Table 8:** Carinated ‘burins’–abrasion of the last negative.

	D1/2	D3	D4	Total
**present**	8	5		13
**absent**	13	16	3	32
**Indet.**	2	1	3	6
**Total**	23	22	6	51

The carinated ‘burins’ from Hayonim Cave portray all the different preparation and maintenance products recognized in the European Aurignacian material (Fig 17, [51]).

### Statistical evaluation

The patterns and divisions of the operational chains from Hayonim Cave can be tested for their validity with multivariate statistical analysis. Towards this aim, the data gained from the attribute analysis of both carinated core types was used as the primary dataset, and the interpretations or observations made, as covariables. The target of this analysis was to evaluate the meaningfulness of the interpretations suggested above. Three assemblages were tested, all carinated elements, only the carinated ‘burins’ and only the carinated ‘endscrapers’. For each assemblage the (1) size groups, (2) the core types (carinated and nosed ‘endscrapers’; single carinated ‘burins’, multiple carinated ‘burins’ and flat faced carinated ‘burins’), and the (3) archaeological level attribution were tested for significance (Table 9). The basic question herein was: do the different (1) size classes (2) core types, or (3) levels significantly correlate with tendencies in the attribute analysis data? Does the attribute analysis record significant and recurrent differences which can be explained by the definitions (size class, core type) or observations (levels) we have at hand? Can we thus consider these definitions or stratigraphic data meaningful for an archaeological interpretation and can we consider potential causal relationships between the technological lithic data and our observation?

**Table 9:** Multivariate analysis of the attribute analysis of carinated elements.

	all carinated elements		carinated ‘endscrapers’		carinated ‘burins’	
	p =	%	p =	%	p =	%
(1) Size class	<1e-04	4.211	<1e-04	3.01	0.3433	
(2) Core Type	<1e-04	13.163	<7e-03	3.791	<1e-04	15.856
(3) Arch. Level	0.5934		0.163		0.028	5.396

F = statistical significance below 0.05, % = percent of explained inertia.

The size classes were found to be significant in the carinated ‘endscrapers’ (p = <1e-04), yet not in the ‘burins’ (p = 0.3433). The positive result of the ‘endscrapers’ and their numerical dominance in the assemblage is responsible for the significant result of the complete assemblage of carinated items (p = <1e-04), therefore the initial division of the carinated ‘endscrapers’ assemblages into large and small varieties was justified. The core type of carinated ‘endscrapers’ shows a significant difference between nosed and non-nosed endscrapers (p = <7e-03), yet the much-reduced explanatory value in comparison to the burins (3.8% in endscrapers vs. 15.9% in burins) confirms the creation of a notch to be an optional maintenance operation, which includes less pronounced differences. For the carinated ‘burins’ the three discussed size classes are insignificant (p = 0.3433) for the explanation of the patterns in the primary dataset. This means that the attributes analysed in the attribute analysis of the ‘burins’ do not change according to the size of the item. It appears that ‘burins’ of all sizes were knapped the same way and differences in production method are not correlated to the size of the ‘burins’. The different core types of the ‘burins’, however, do include a significantly different pattern in recorded attributes (p = <1e-04) and thus a different knapping method of the items.

The archaeological level is insignificant when referring to the carinated ‘endscrapers’ (p = 0.163), while the carinated ‘burins’ (p = 0.028) show a weakly significant result. When combined, the result remains insignificant (p = 0.5934). This indicates that the attributes recorded for the ‘endscrapers’, and thus their production method, do not change according to stratigraphic chronology. On the other hand, the production method of the ‘burins’ does show a chronological relation.

### Bladelets

**Plate 14:**
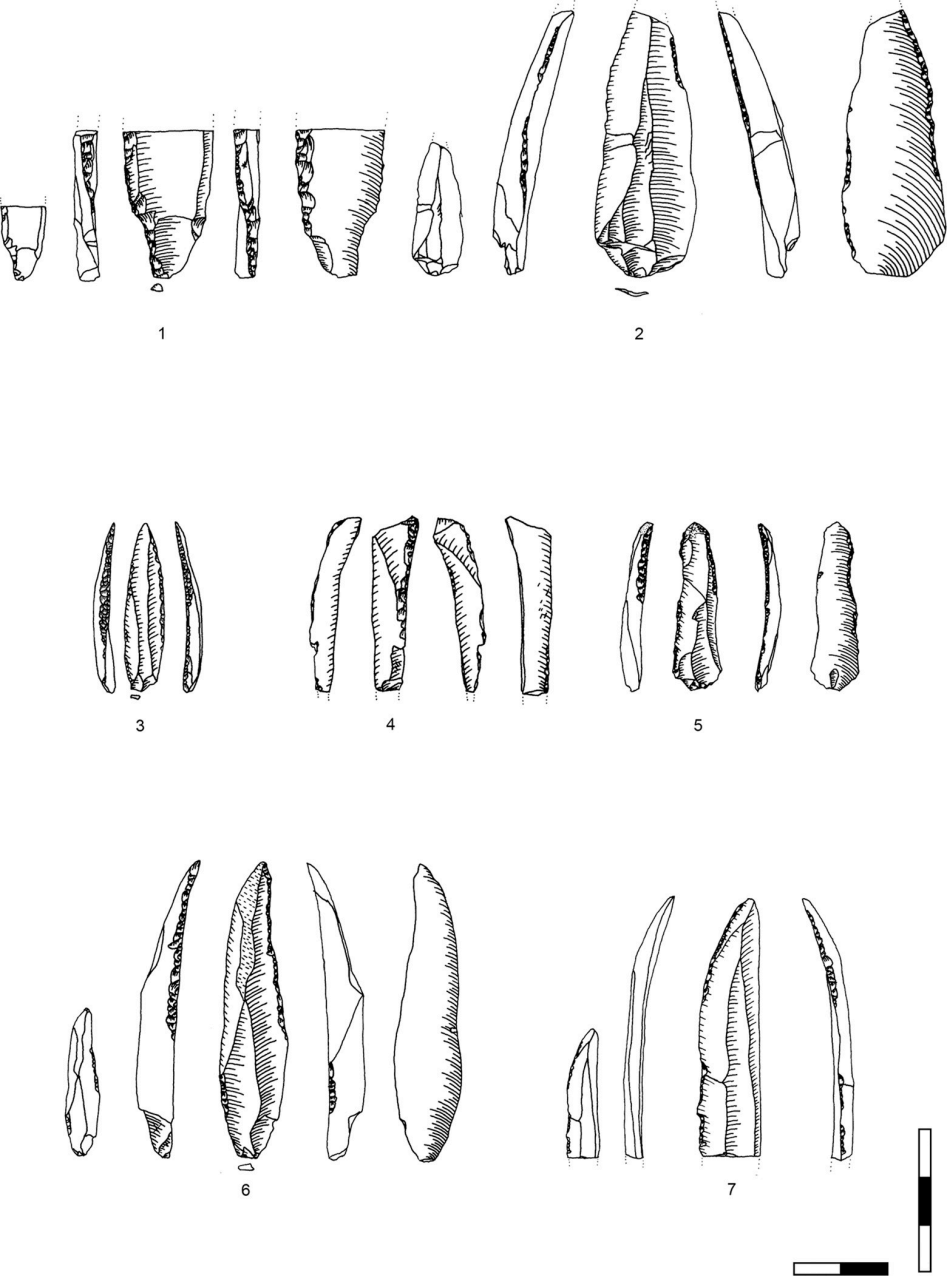
Retouched bladelets. 1–2, 6 D1-2; 3–5, 7 D3. 1–2, 6–7 enlarged, original size on the left.

**Plate 15:**
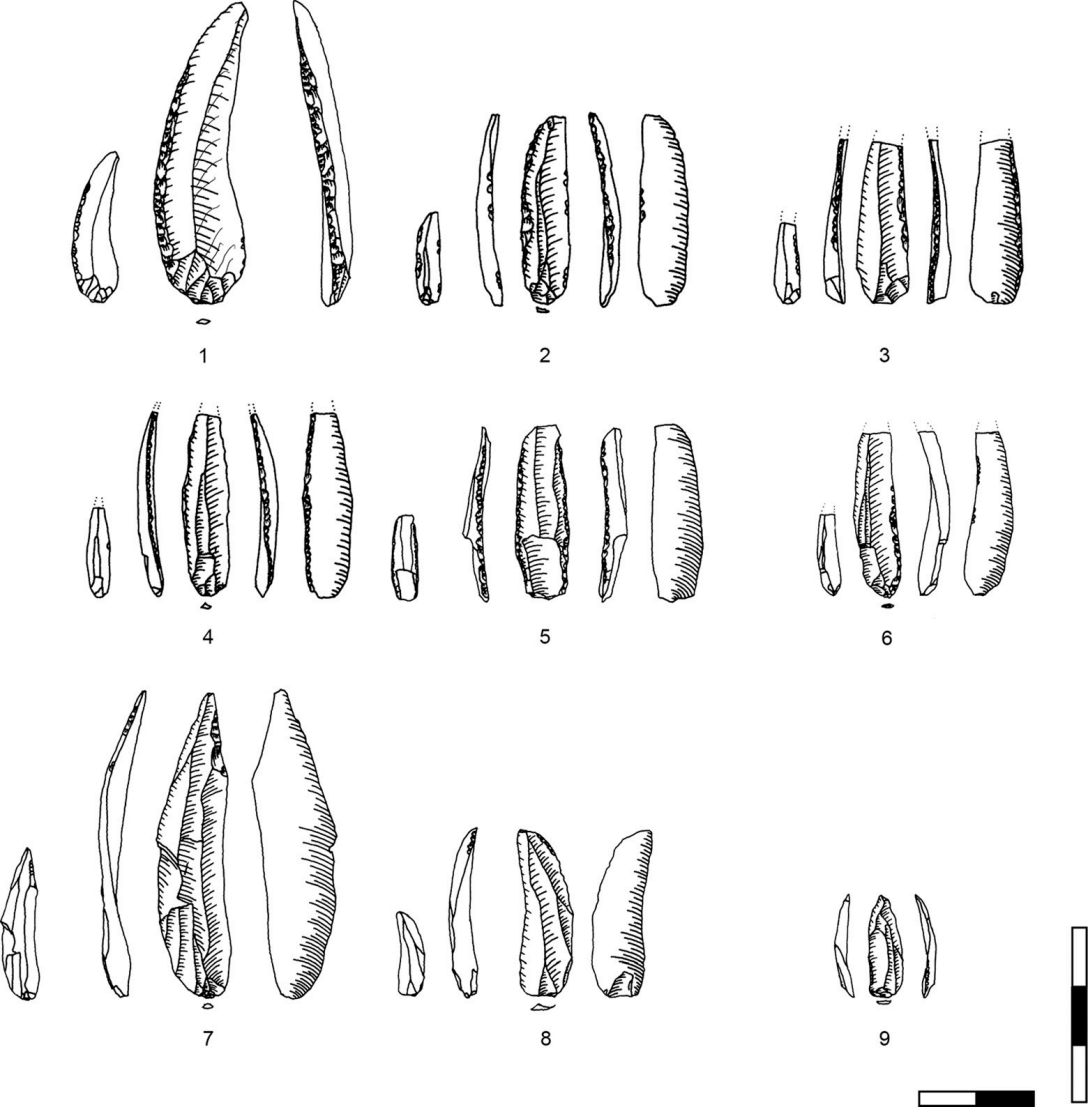
Retouched bladelets. 1, 3, 5 D1-2; 2, 4, 6, 9 D3; 7–8 D. 1–8 enlarged, original size on the left.

**Plate 16:**
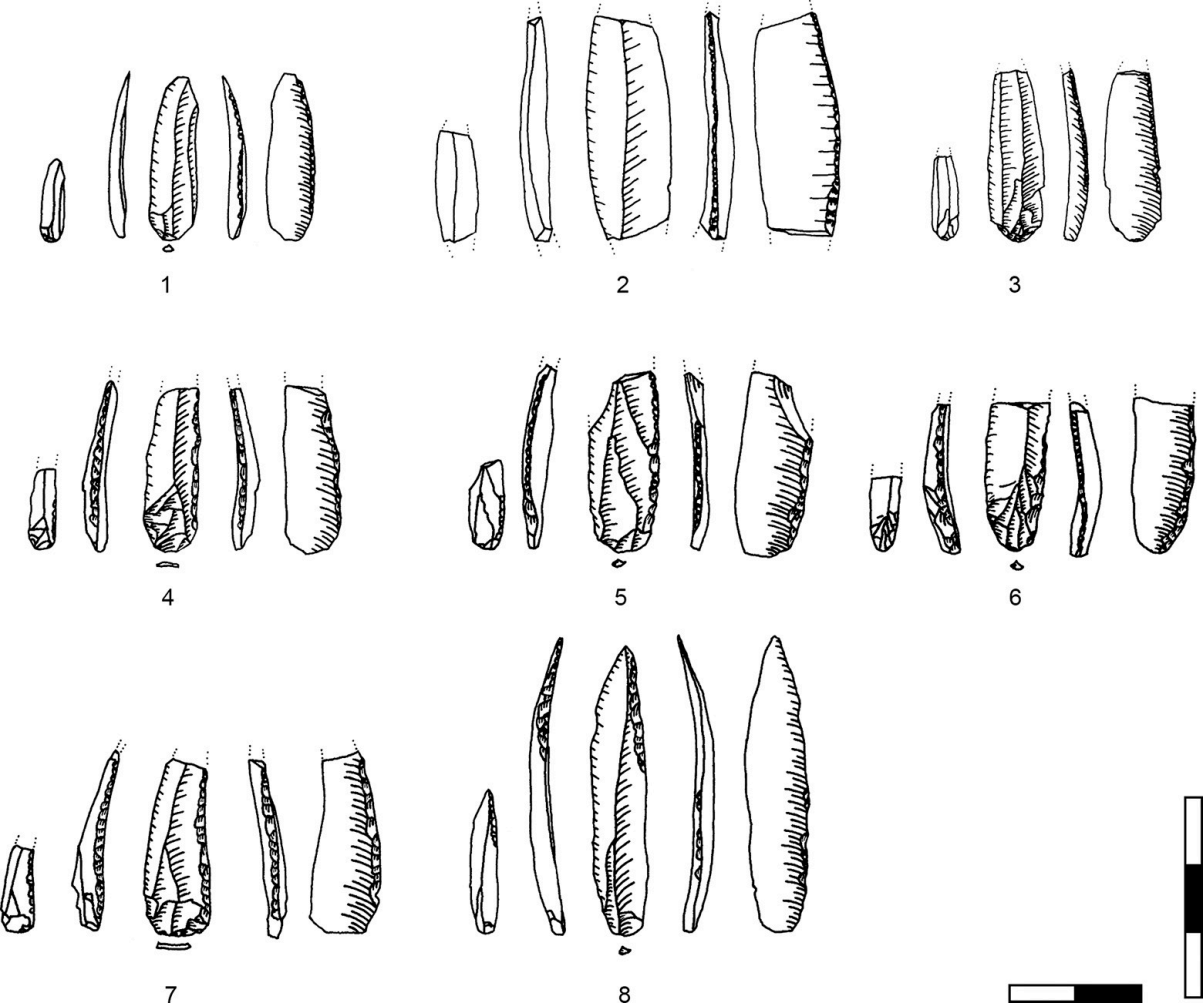
Retouched bladelets. 1, 4–8 D3; 2 D1-2, 3 D4. 1–8 enlarged, original size on the left.

The bladelet assemblage in Hayonim Cave D comprises 520 items analysed in detail through attribute analysis as well as 887 additional ones which were only counted, thus 1407 bladelets in total. These are distributed over all stages of the operational chain including target products (Plate 1.1–5), reduction by-products (Plate 1.6–10; Table 13 in S1 File), and preparation and maintenance products (Plates 3, 8, 10, 13) as well as tools (Plates 14–16).

**Fig 20:**
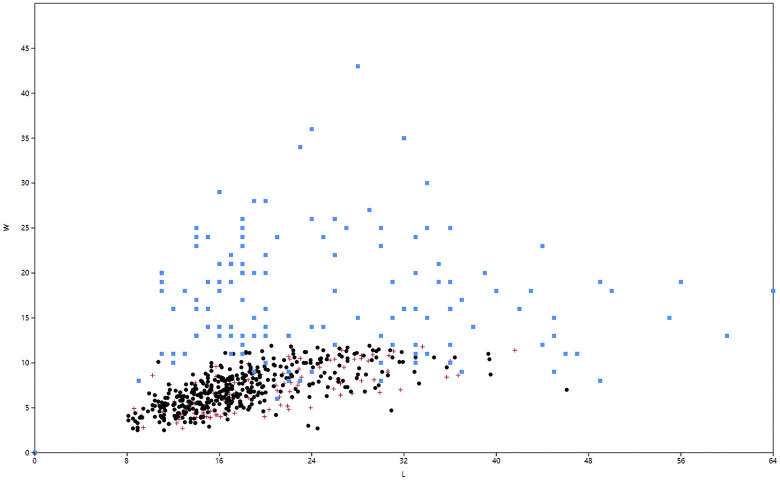
Hayonim Cave bladelets (black), retouched bladelets (red) and core reduction surface (blue). Length and width measures.

The retouched and unretouched target bladelets form a uniform assemblage by dimensions (Fig 20, red+black). Furthermore, the length of the reduction surfaces on the cores also matches the size distribution of the bladelets (Fig 20, blue).

The results of the attribute analysis on the Hayonim Cave bladelets are available in the supplementary material (Tables 14–28 in S1 File). Briefly summarized, the knapping characteristics point towards the use of a soft (stone or bone/antler) hammer in tangential use due to the predominance of ridge-shaped or punctiform platforms, diffuse bulbs, the rarity of flake scars and concordant presence of flake lips, acute knapping angles and flat fracture terminations. Reduction is predominantly unidirectional with only intermittent cross negatives, which are the remains of preparative actions. Unretouched and retouched target bladelets generally closely match in characteristics, while the by-product bladelets show a more diverse range of characteristics. Target bladelets are generally straight or twisted to the right, show a triangular or trapezoidal cross section and a regular, aligned or converging dorsal scar pattern (Plate 1.1–5). On the other hand, by-product bladelets (Plate 1.6–10) preserve a clear lateralisation with remains of a regular reduction on one side of the item and remains of ventral faces or preparative actions on the other lateral. Morphologically, the unretouched target bladelets and the bladelet tools (Plates 14–16) correlate, as only very few by-product bladelets were modified into tools (Table 29 in S1 File, e.g. Plates 14.4; 15.8).

With regard to the correspondence of the bladelets to the potential cores (hence the carinated ‘burins’ and ‘endscrapers’), some lines of evidence can be followed. Abrasion of the platform edge is commonly observed (Table 20 in S1 File) indicating a maintenance of the platform edge during reduction, as can also be found on some of the preserved cores. About 58% of the bladelets are twisted (Table 24 in S1 File), with a strong predominance of right-sided twisting over left-sided twisting, which correlates to the orientation of the cores (Table 1 in S1 File, 10). Both, straight bladelets and cores, indicate an occasional left-sided twist of the products, mostly observable in the by-product bladelets, the lateralised marginal products of the reduction.

In total, 35 bladelets preserve the remains of ventral surfaces (Table 25 in S1 File) thus securely establishing their origin from a core-on-flake. Ventral remains are more commonly found on the by-product bladelets, again due to their origin from the lateral portions of the reduction surface as well as from the earlier stages of reduction. These ventral remains are found both on the right and left lateral sides of the bladelets (Table 26 in S1 File). This does not clearly correlate with the predominantly right-sided orientation of the cores, which should result in a predominance of ventral remains on the right side of the bladelet. However, ventral remains on the small bladelets are hard to securely identify thus it is possible that not all have been recognized on the bladelets. The good correspondence between the twisting of the cores/carinated items and the twisting of the bladelets can thus imply both, a better sample size and a more valid sign for the origin of the bladelets. Yet the very presence of ventral remains on the bladelets is a proof of their origin from cores-on-flakes, most likely the carinated ‘burins’ as they constitute the majority of the cores-on-flake available in the assemblage.

The retouched bladelet assemblage (Table 10) primarily contains pointed bladelets with fine retouch (n = 21, 18.9%) or fragments thereof (n = 20, 18.0%). There are also bladelets with inverse retouch (n = 11, 9.9%), with only partial retouch (n = 13, 11.7%) or with macroscopically visible use-wear but no other formal retouch (n = 12, 10.8%). Alternate retouch (n = 9, 8.1%) is present but less frequent. A comparison of the different sub-levels does not lead to a fundamentally different picture, except for the fragmentation rate of finely retouched bladelets being much higher in D1–2 than in D3. Most retouch is marginal (n = 97, 87.4%) and instances of formal backing are rare (n = 12, 10.8%). Retouch (Table 30 in S1 File) is predominantly located on the left dorsal side, either continuous (n = 24, 21.6%) or intermittent (n = 18, 16.2%).

**Table 10:** Bladelet tools typology.

		D1/2		D3		D4		Total	
		n	%	n	%	n	%	n	%
**J01**	Pointed bladelet with fine retouch	6	11.1	13	25.5	2	33.3	21	18.9
**J02**	Blunt bladelet with fine retouch	5	9.3	3	5.9			8	7.2
**J04**	Fragment of bladelet with fine retouch	13	24.1	6	11.8	1	16.7	20	18
**J09**	Fragment of bladelet with abrupt retouch			1	2			1	0.9
**J10**	Bladelet with inverse retouch	5	9.3	5	9.8	1	16.7	11	9.9
**J11**	Bladelet with alternate retouch	2	3.7	7	13.7			9	8.1
**J15**	Truncated bladelet	4	7.4	3	5.9			7	6.3
**J16**	Backed and truncated bladelet			1	2			1	0.9
**J71**	Retouched/backed bladelet fragments					1	16.7	1	0.9
**J90**	Backed bladelet	1	1.9					1	0.9
**J91**	Partially retouched bladelet	7	13	6	11.8			13	11.7
**J93**	Bladelet with bilateral retouch	1	1.9	4	7.8			5	4.5
**J97**	Partially backed bladelet					1	16.7	1	0.9
**X1**	Macroscopical use-wear	10	18.5	2	3.9			12	10.8
	Total	54	100	51	100	6	100	111	100

### Preparation and maintenance typology

All three, carinated ‘endscrapers’, ‘burins’ and bladelet assemblages (Table 11) were, additionally to technological characteristics, subjected to a classification according to the ‘typology of technological products‘ (Fig 5, first box) intended to identify typical preparation and maintenance products from carinated reduction sequences similar to those observed in western European Aurignacian assemblages [37, 44–49, 51]. Most clearly identifiable and most indicatory are the remains of striking platform preparations in the form of ‘type Thèmes’ bladelet (No. 9–10 in Fig 5) reflecting initial preparation (9) and subsequent maintenance (10) of the striking platform. In total 18 such bladelets are preserved in layer D, seven of which preserve the first preparation of the platform (Plate 10.1–3) and eleven stem from the second removal (Plate 10.6–8). There are also ‘regular’ core tablets from the preparation and maintenance stages, which do not show remains of lateral platform faceting. Indication of initial preparation (n = 12; Plate 3.1–2, 5–8, 10–13) refers to the presence of bulbar negatives on the platform of the tablet hence the reduction surface of the core, while maintenance indication (n = 3; Plate 3.3–4, 9) refers to the removal of these bulbar negatives. All of these portray a presence of a ventral face on one of their laterals, again, indicating their origin from a core-on-flake. One specimen (Plate 3.11) even shows the remains of a thick bulb, struck with a hard hammer, on the distal-right-lateral face.

**Table 11:** Typology of technology Hayonim Cave D.

CO Stage	No	Type	D1/2		D3		D4		D	Total		Grand total
			AA	Count	AA	Count	AA	Count	Count	AA	Count	
**Preparation**	1	initial crested	5	2	5	1	1	0	8	11	11	22
**Maintenance**	2	distally crested	5	1	2	6	1	0	13	8	20	28
**Reduction**	3	plain	111	110	140	169	30	31	67	281	377	658
	4	first order lateral	44	94	36	90	11	18	85	91	287	378
	5	second order lateral	20	44	17	50	5	8	49	42	151	193
	6	third order lateral	3	5		7	6	1	17	9	30	39
**Preparation**	7	initial tablet	5	1	3	1	1	0	1	9	3	12
**Maintenance**	8	secondary tablet	1	0	2	0		0	0	3	0	3
**Preparation**	9	Thèmes initial	1	1	4	0	1	0	0	6	1	7
**Maintenance**	10	Thèmes secondary	4	0	4	0	3	0	0	11	0	11
**Discard**	11	Core	54	0	48	0	9	0	0	111	0	111
**All**	12	Other	64	0	77		18	0	0	159	0	159
	13	Indet.	6							6	0	6
**Maintenance**	15	Carinated ‘endscraper’ lateral correction		2	5	3		0	0	5	5	10
	17	Carinated “endscraper’ removal scraper cap	2	1	1	1	2	0	0	5	2	7
		Total	325	261	344	328	88	58	240	757	887	1644

AA = items from the attribute analysis, count = additionally counted bladelets.

Reduction surface preparation is indicated by a series of well-developed crested bladelets, either (1) initial ones (n = 22; Plate 13.4–5) from the preparation, or (2) distal crests (n = 28; Plate 13.6–11) from the maintenance stage, which can be again, primary (Plate 13.6–8) or secondary (Plate 13.9–11). All these specimens stem from carinated ‘burin’ reduction sequences, present in their entirety in Hayonim Cave. Judging from the ‘burins’ themselves as well as their products, distal stopping notches (Fig 5, box 1 no 6) were not present at all in the layer D assemblages.

Carinated ‘endscraper’ reduction sequences leave fewer characteristic preparation and maintenance products compared with carinated ‘burin’ reduction, yet the few present are unmistakable. Foremost among these are items resulting from maintenance actions of striking off parts of the ‘endscraper’ bit to re-establish the lateral notch required for the twist of the products (17). These are present in each D sublayer, seven specimens in total (Plate 8). Most of these show on their lateral side a series of pronounced hinges indicating the necessity of correction. There was just one item, a core tablet, struck off a carinated ‘endscraper’ with two ventral surfaces and bearing, proximally, the remains of the former reduction surface (16). By accident, it was not included in the attribute analysis.

### Statistical evaluation

Again, multivariate statistics can be used to explore the meaningfulness of the interpretations of the material. The primary dataset includes the complete attribute analysis incorporating the information on retouch type and locality as well as tool type (for results see supplementary material). Covariables comprise the archaeological level, the operational chain stage, the specific product, and the preparation-and-maintenance typology. The results are presented in Table 12, which specifies the significance level (p) and the percent of explained inertia of the covariable.

**Table 12:** Multivariate analysis of the attribute analysis.

Covariable(s)	p =	% of explained inertia
**Level**	0.0197	0.413
**CO-Stage**	<1e-04	2.033
**CO-Product**	<1e-04	3.814
**Prep/Mtn Typology**	<1e-04	2.911

The analysis shows a weak significance of the level attribution (p = 0.0197, % inertia = 0.413) indicating a minor chronological relevance for the result. All three other covariables are highly significant (p = <1e-04) and explain the variability to ~2–3.8%. The comparatively low values of the bladelet statistics in comparison to the cores can most likely be attributed to the generally larger dataset in terms of rows and columns of data, and the generally lower variability that an attribute analysis can catch in bladelets in comparison to cores.

The coarse division of the operational chain stage ‘CO-stage’ explains the lowest amount of inertia (2.033%), while the other two covariables, ‘CO-Product’ (3.814%) and ‘Preparation/Maintenance typology’ (2.911%), show higher amounts of explained inertia. This picture shows how the finer divisions of specific types increase the explanatory potential of the analysis and more accurately reflect the differences between the attributes recorded for the bladelets. The results of the multivariate statistics thus support the a-priori division of explanatory classes and the manually formed interpretations of the data from the attribute analysis.

### Non-carinated core reduction sequences

Lastly, we shall discuss the non-carinated cores in order to gain perspective on the carinated operational chains and to be able to preliminarily assess the rest of the reduction activity on site.

Towards that aim 239 cores were analysed, classified, and sorted into reduction strategies. Twenty-three of these cores were removed from the analysis as they originate from a mixed locality. They concur with the tiny Natufian bladelet cores and are thus unlikely to belong to the Aurignacian occupation. The remaining 216 items comprise 97 cores from sublayer D1–2, 77 from D3, 32 from D4 and a further 10 which can only generally be correlated to layer D.

The focus of the non-carinated reduction activity on site in all levels is bladelet and flake production with an emphasis on bladelet cores (Table 13). While in D1–2 and D4 bladelet and flake cores are nearly equal in representation, in D3 simple bladelet cores are the majority. All inventories yield cores that still preserve negatives of blade dimensions, yet in all sublayers they remain below 10%. A more detailed study of the non-carinated operational chains will be required to understand if the low numbers of blade cores are a product of advanced core exploitation or if blades were generally less sought after. From a very cursory glance into the non-carinated preparation and maintenance products, a presence of formal crested- and neo-crested blades of large dimensions (~10 cm in length) can be reported, which in turn would indeed point to the presence of a formal blade production of larger blanks. All inventories also contain a low share of cores that combine the production of either flakes and bladelets, or flakes and blades.

**Table 13:** Target products of non-carinated cores.

	D1/2		D3		D4		D		Total	
** **	n	%	n	%	n	%	n	%	n	%
**indeterminable**	7	7.2	6	7.8	4	12.5	1	10	18	8.3
**flakes**	37	38.1	23	29.9	13	40.6	4	40	77	35.6
**blades**	8	8.2	9	11.7	1	3.1	1	10	19	8.8
**bladelets**	40	41.2	36	46.8	13	40.6	3	30	92	42.6
**flakes and bladelets**	3	3.1	2	2.6	1	3.1			6	2.8
**blades and flakes**	2	2.1	1	1.3			1	10	4	1.9
**Total**	97	100	77	100	32	100	10	1000	216	100

Nodule selection for the non-carinated operational chains predominantly focusses on the acquisition of fresh, full, and roundish nodules from the outcrops surrounding the site (Table 31 in S1 File). The common raw material are local Eocene and Cenomanian flints, yet raw material types from as far away as the Carmel range could be identified. The use of nodular flint clearly outweighs the use of tabular flint, which might be a deliberate selection related to the wanted core shapes. Non-carinated cores-on-flakes are the second largest group in all D assemblages, thus the general idea of a bipartite operational chain including the production of large blanks that can serve as cores is not exclusively correlated to the production of carinated items. However, the non-carinated cores in Hayonim Cave in their present form do not immediately support the interpretation that they are the source of large blanks, especially for the larger non-carinated cores-on-flakes. Naturally, initial nodule preparation often and easily can yield blanks of sufficient size to serve for the smaller carinated items. An additional method of blank sourcing is preserved on one core-on-flake, as well as on a few carinated items (e.g. Plate 12.4), which is the acquisition of large blanks directly from the outcrop walls. These blanks are characterised by uncommonly large and pronounced bulbs with clear hard-hammer percussion marks and knapping characteristics. This type of blank acquisition has previously been observed in the Post-Aurignacian carinated burin production in southern Jordan [33], and attention should be paid to it in the analysis of future Aurignacian-related inventories in the Southern Levant as it appears to be a distinctive feature of carinated core blank acquisition.

An opportunistic nodule selection does play some part in the sourcing of flints in all D assemblages as does the reuse of older knapped flint items [84]. All in all, the nodule selection is targeted and planned, fresh, whole nodules comprise the largest part, fluvially transported items are virtually absent, while rolled pebbles and formerly knapped items are quite rare. This is in stark contrast to the previously mentioned Post-Aurignacian assemblage [33] with comparable large-flake sourcing, which is far more opportunistic in raw material acquisition and far more ‘tolerant’ of inferior quality nodules compared with the Hayonim Cave D assemblages.

Exactly the same picture is reflected in the cortex types preserved on the cores (Table 32 in S1 File). In all assemblages the share of cores with fresh cortex reaches or exceeds 60%, fluvial and strongly battered or abraded surfaces are absent; items with patinated old surfaces represent less than 15% in all D assemblages. Items with a combination of fresh and old surfaces are equally frequent, but such old surfaces can occur *in situ* on the outer faces of flint outcrops due to rockfall, previous human sourcing, thermal fracturing and weathering or other natural agents and are not indicative of a secondary use.

**Fig 21:**
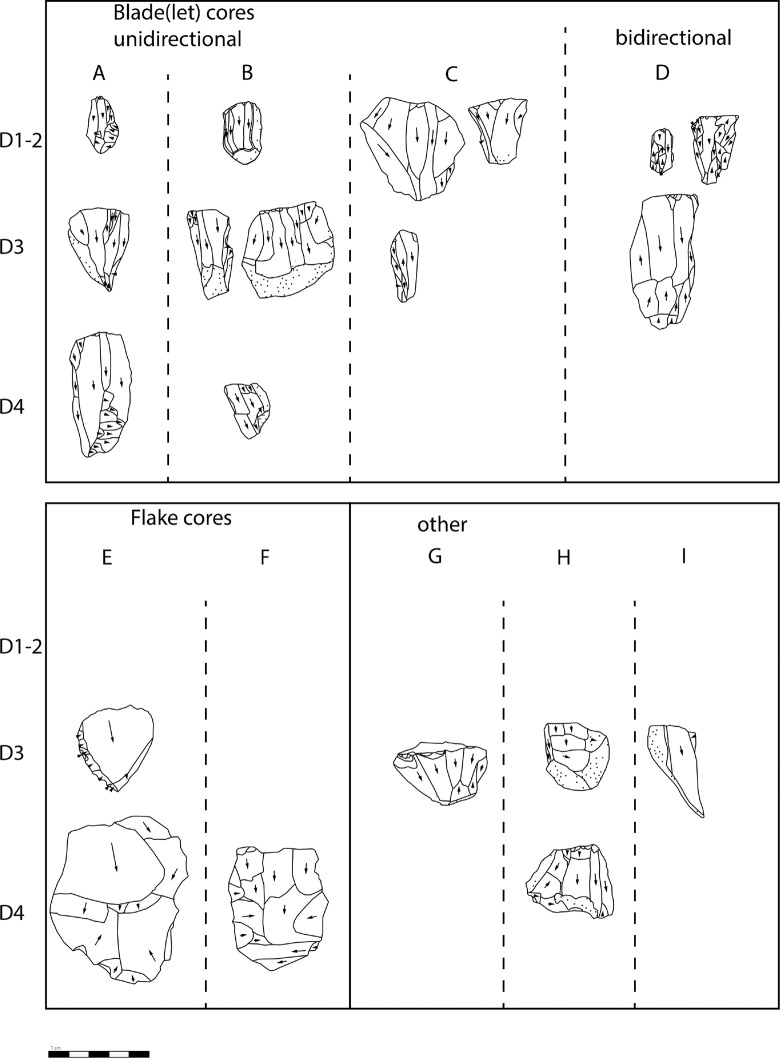
Major core types.

The non-carinated cores are characterised by a wide variability in type (Table 33 in S1 File) and shape (Table 34 in S1 File), yet with a dominance of unidirectional and parallel core setups. Interesting, however, is the general bandwidth of core setups, including bidirectional, 90°-turned cores, with reduction surfaces on opposite faces of the nodule, and with multiple and discoid flaking directions. Several cores do look like other known concepts, as Levallois, but only superficially. Other cores resemble the carinated ‘burins’ or ‘endscrapers’, but all of them lack distinct criteria that would allow a formal determination of a carinated element (e.g. the presence of only one reduction negative–Fig 21, I).

Blade and bladelet cores generally show comparable characteristics and appear as a flowing continuum from the one to the other. They can be broadly divided into uni- and bidirectional cores (Fig 21). The unidirectional cores were subdivided into three classes. Class A includes converging or sub-converging cores with distal-lateral maintenance actions to (re-)establish the distal convexity of the core which results in neo-crested blades as the maintenance product, several of which could be identified during the cursory assessment of the preparation products (see above). Some of these cores approach the narrow-fronted concept of the Ahmarian, yet overall, they lack the formality and regularity observed therein, as well as the intensive preparation sometimes encompassing also the back of the core. Class B comprises very opportunistic cores, which generally lack a designated core preparation and retain large cortex coverage, and their reduction negatives are generally parallel to each other. They can be narrow and roundish or flat and broad (see Fig 21 D3: B). Such a core type is very *ad hoc*, quickly fashioned from suitable roundish elongated nodules. Class C cores portray similarities with those of Class A, yet with large lateral shaping negatives that narrow the distal part of the core and establish the convexity for a converging approach. Bidirectional cores are quite rare, but the ones that are present are true bidirectional cores with a generally balanced or only slightly weighted reduction of both platforms. This means that the opposite platform is indeed meant for reduction purposes and not only for a facilitation of maintenance activities. It is distinctively different from the carinated operational chains, which conceptually cannot allow for a bidirectional reduction due to the pronounced (carinated) convergence of the reduction face.

Flake cores are generally opportunistic, (i.e. of multiple directions and platforms) representing most of the amorphous and discoid cores in the assemblages. A few of these cores resemble Levallois cores (Class E), but on closer inspection do not fit into the standard Levallois criteria. These cores might originate from the Middle Palaeolithic occupation of the cave, either intrusive or purposefully reused by the Aurignacian occupants, and are missing double patination due to chemical circumstances in the depositional environment. However, they also may just be regular flake cores of the Aurignacian reduction sequence. One clear double patinated Middle Palaeolithic Levallois core is present in Hayonim Cave D, which has been recycled into a carinated ‘endscraper’ by the Aurignacian occupants of the cave (Plate 4.7) The known and previously-observed reuse of Middle Palaeolithic Levallois cores by Aurignacian occupants [84] does ascertain that they are familiar with them, and as good flint-knappers are certainly also able to ‘read’ the Levallois reduction concept and understand the logic behind it, which in turn might have influenced their own approach to flake cores. A more detailed reconstruction of the Aurignacian flake core reduction is certainly warranted and, especially with the above interpretation in mind, would also be of significance in terms of learning strategies and technological traditions. Flake cores representing Class F, characterized by distal lateral corrective blade(let) negatives, appear only in sublayer D4. Their overall reduction concept places them among the flake cores and identifies those lateral negatives as correctional rather than reflecting actual reduction of target items.

Other core types do include all-around pyramidal varieties (Class G), 90°-changed orientation cores (Class H) and the already mentioned carinated-like cores (Class I). From a knapping conceptual view, those can most likely still be placed among the carinated items, and simply represent either the (typologically) ‘wrong’ blank (as e.g. a re-used Levallois core (Plate 4.7), a core on chunk [= rabot]) or the presence of only one reduction negative, which might have easily been a knapping error. Even if using a typological approach of the classification of carinated ‘burins’ and ‘endscrapers’ such items are excluded, from a conceptual and technological viewpoint a chunk might easily be a suitable blank and would result in a carinated core-on-chunk. This is indeed an archaeological terminological issue touching on the distinction between cores-on-chunks/nodules and cores-on-flakes, which is of course pivotal in the discussion of carinated items. Here we would like to argue, that the classification should indeed be made according to the conceptual setup of the carinated face producing bladelets and no distinction should be made whether it appears on a flake or a chunk; this blank difference can and should be discussed as regards core-blank selection. The equifinality of the resulting bladelets, the intentionality and choice of the Aurignacian flint-knappers, should override the arbitrary classification of cores or not cores.

The one platform-one reduction surface configuration is generally dominant (Table 35 in S1 File), but up to four of each are possible. Interestingly, multiple platforms and reduction surfaces appear to increase over time, as it seems general core complexity does. With regard to sublayer D4 though, this might possibly reflect no more than the much-reduced sample size.

Finally, a number of binary variables are evaluated: burning, possible heat-treatment, core breakage and twisting (Table 36 in S1 File). The distinction between ‘burning’ and possible ‘heat-treatment’ was made on account of morphological differences. This means an item classified as burned can be understood as ‘destroyed by fire’, i.e. the flint shows cracks, potlid fractures, colour changes and all other signs of heavy burning. Possible heat-treatment, on the other hand, means that the flint has clearly seen fire, due to colour and texture changes, but is not ‘destroyed’, there are no cracks, no fissures, no potlids. Essentially, the core might still be usable after the fire impact, which is what would be expected of heat-treated items. Yet obviously, this is not enough for an actual confirmation that heat-treatment was practiced in the Levantine Aurignacian in Hayonim Cave. Furthermore, the cores might have been heated due to Natufian hearth features overlying the Aurignacian layer. A detailed study of the matter would be warranted to investigate this further. It would be a rare event in any case, comprising only 30 cores in total, which is about 14% of the inventory, most of these originate from D1–2. Core breakage is rare, (5.6% of the core assemblage); similarly, the reduction surface of the non-carinated cores in Hayonim Cave is generally not twisted, as only 12.5% show a twisted reduction surface.

The overall impression of the non-carinated core assemblage of Hayonim Cave D is one of diversity. Many different concepts, knapping directions, and core shapes were employed to gain target products, i.e. flakes and bladelets. It is unclear if blade cores are absent due to a continued reduction unto bladelet cores, or if they were missing from the beginning. Secondary indication points towards a continued reduction. Raw material acquisition had a clear target nodule shape in mind, with but a few exceptions. The introduction of bidirectional blade(let) reduction concepts into the Levantine UP is notable, as is a curious persistence of ‘Levallois-like’ cores. The latter raise the question of whether they originate from the Middle Palaeolithic, as either intrusive or recycled items, or if they were produced *in situ* by the Aurignacian flint-knappers. The core-blanks for the carinated reduction sequences cannot directly be connected to the non-carinated cores present in the assemblage, a matter that might be a result of extensive exploitation or off-site acquisition and production of carinated core blanks. A more detailed assessment of the non-carinated operational chains, cores, and products, would be a highly desirable research question for the future.

## Summary and discussion

### Summary

The combination of operational chain reconstruction, product typology and statistical evaluation strongly supports the hypothesis that the carinated items (‘endscrapers’ and ‘burins’) in the Aurignacian assemblages from Hayonim Cave may have functioned as cores. An integral, entirely preserved, operational chain producing the same products identified in Aurignacian assemblages from Western Europe [37, 44–51] is a key argument for such a claim, short of refitting. Furthermore, the statistical evaluation can ascertain the classification of operational chain products via attribute analysis. The results of the present study, however, do not rule out a potential use of these items as tools, most likely after being discarded as failed/exhausted cores. This can be proven via a use-wear study which was beyond the scope of the present investigation. Such a flexible behaviour can be expected in Palaeolithic hunter-gatherer societies and has been suggested for other tool types. For example el-Wad points could be shown to be used also for drilling purposes [85]. Furthermore, it is not uncommon for ground adzes or axes to be reused, or exploited as cores in the German and Levantine Neolithic, after their primary function was terminated (due to breakage or exhaustion), or the bearer has acquired a replacement [86]. Ground-stone tools show a far higher investment in their manufacture than chipped stone tools in general [87], thus indicating that essentially no tool type can be excluded from repurposing flexibly according to needs or even just convenience. The reconstruction of the carinated ‘endscraper’ operational chains by and large confirms the results already gained by Michael Chazan [55] yet extends it to discuss the preparation and maintenance items in greater detail as well as presenting the operational chains and their by-products of carinated ‘burins’.

### Chronology

**Fig 22:**
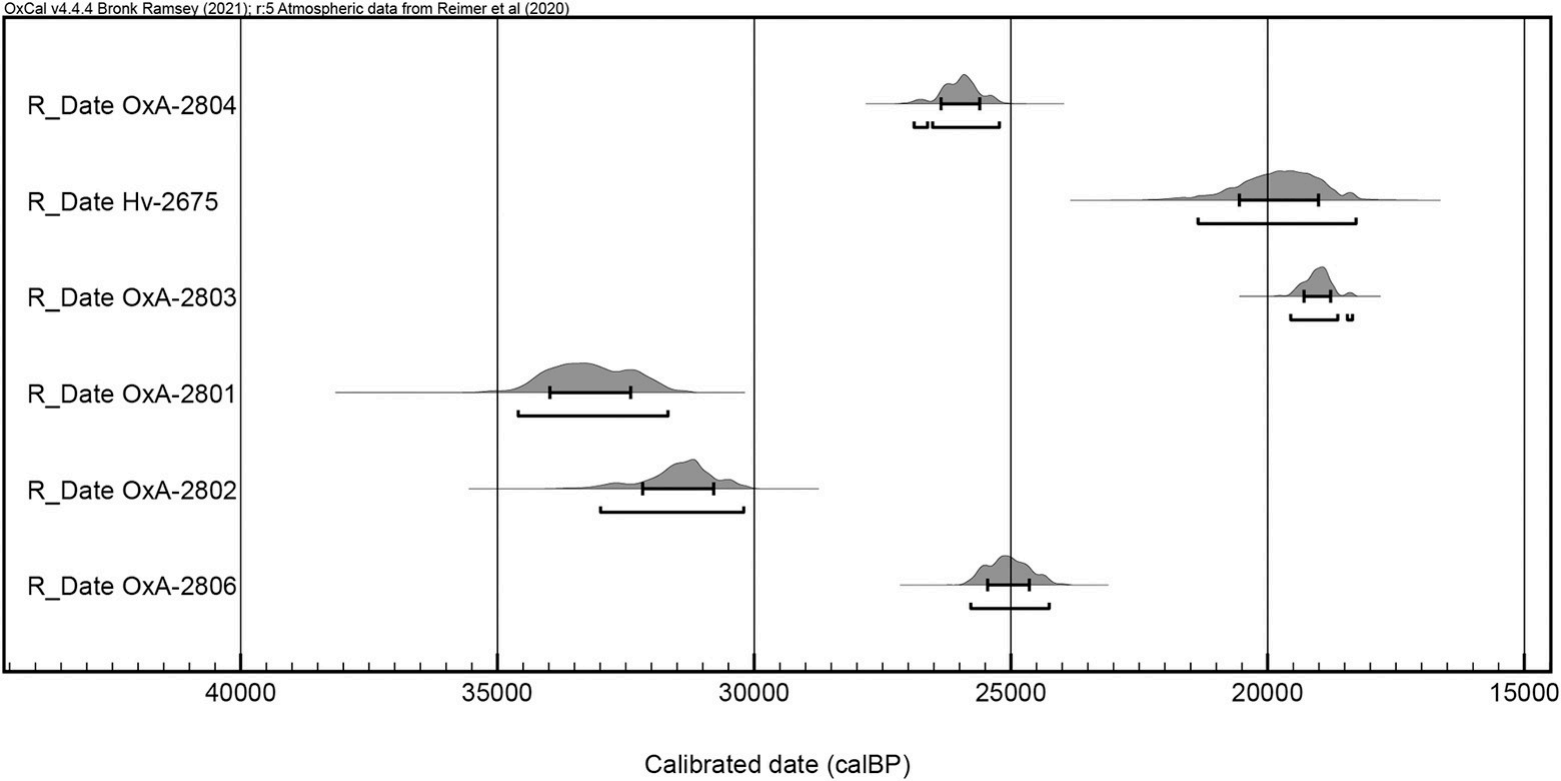
Hayonim Cave ^14^C Dates. Calibrated with Oxcal 4.4 [88, 89].

Six ^1^⁴C dates are known from Hayonim Cave (Table 14, Fig 22) all of which predate modern AMS dating methods. Even when calibrated with the newest calibration curve Intcal20, two dates fall below the 20ka threshold, two more lie in the mid 20ka´s and only two predate 30ka, although results have improved (read ‘gotten older’) compared to Intcal13 [33]. Dates from Manot Cave obtained by the most modern state of the art technology including Bayesian modelling [7] place the Levantine Aurignacian occupation there at 35–37calBP, therefore still substantially older than the oldest dates for Hayonim Cave. This divergence, and especially the four young dates for Hayonim Cave can most likely be explained with the advances in ^1^⁴C dating methods. The Hayonim Cave dates can be considered unreliable and should not be considered in a discussion of Levantine Aurignacian chronology.

**Table 14:** 14C Dates of Hayonim Cave D, recalibrated with Oxcal v4.4.4, Intcal20 curve [88, 89].

Level	Lab#	Date uncal BP	1Sigma	Date calBP (Intcal20)	1 Sigma	context	Date	Reference
**D1/2**	OxA-2804	21650	340	25,961	377	D2/H20b/225-230 bone	1994	[90]
**D1/2**	Hv-2675	16240	640	19,725	771	bone	1981	[9]
**D1/2**	OxA-2803	15700	230	19,019	258	D2/J19cd/190-199 bone	1994	[90]
**D3**	OxA-2801	28900	650	33,227	787	J21a/220-230 bone	1994	[90]
**D3**	OxA-2802	27200	600	31,390	692	G22a/220-240 bone	1994	[90]
**D3**	OxA-2806	20810	320	25,062	405	IJ20/200-205 bone	1994	[90]

While the early UP chronology in the Levant still stands on rather shaky feet, a tentative comparison correlates it to the middle-to-later part of the Aurignacian in western Europe [91]. It is currently inadvisable to reconstruct a ‘migration direction’ based on the ^1^⁴C chronology alone, if it is indeed true that these assemblages represent migration pulses from Europe into the Levant. Given the indication that they are roughly contemporaneous, the focus should lie on direct techno-typological comparisons until a more detailed chronology is available.

### Cost efficiency / usability

Chronology aside, one of the major questions related to the introduction of carinated operational chains in the (Levantine) Aurignacian is the potential benefit and the reasons behind the introduction. At frontal value, blanks from carinated cores are smaller and require composite hafting in comparison to an Ahmarian el-Wad point, which is larger, more easily managed, and can be hafted as one implement per projectile. Therefore, the question of ‘why the trouble’ requires actual consideration. Focusing on the Levant, the cost-efficiency of carinated technology vs. Ahmarian narrow-fronted technology should be investigated.

**Fig 23:**
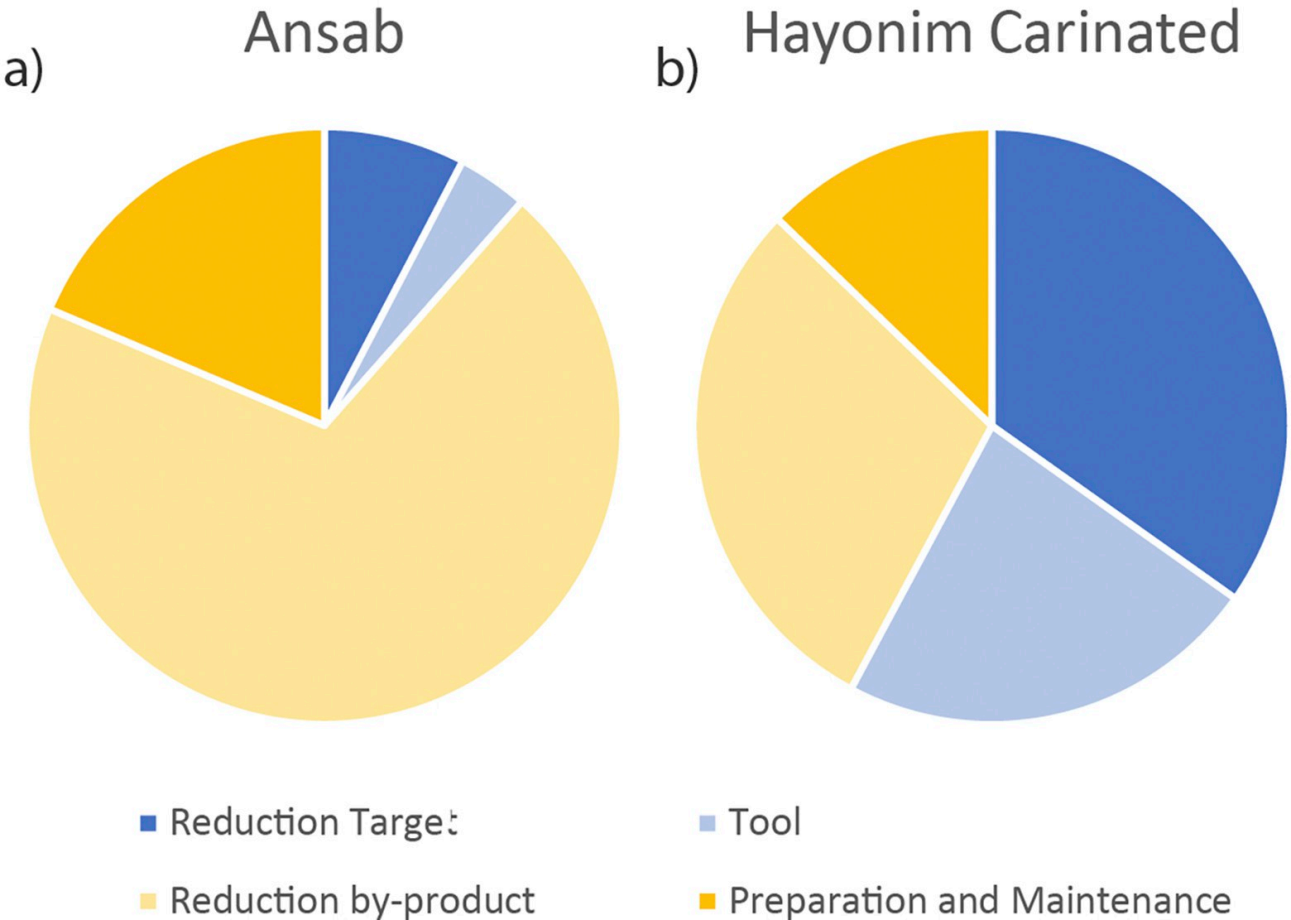
Comparison of the reduction efficiency of different operational chains. a) Ansab 1, Southern Jordan, Early Ahmarian, b) Hayonim Cave D (total), Lower Galilee, Levantine Aurignacian.

Carinated reduction sequences are clearly more cost-efficient than the narrow-fronted core technology of the Ahmarian. A comparison with the Ahmarian site of Ansab 1 [28, 29, 33, 92] shows a greatly increased yield of target products, including tools, in comparison to the Ahmarian reduction (Fig 23). In direct comparison, Ansab includes only 12% tools and target products, while the Aurignacian in Hayonim Cave preserves 58% tools and target bladelets. Import and export dynamics, however, must be taken into account, especially considering the interpretation of Ansab 1 as an acquisition site targeting the available raw material outcrop for the production of blades for export, so both sites are not directly or functionally comparable. But even so, the differences are still startling and a more detailed comparison including more sites would be a worthwhile endeavour. Ansab 1 and Hayonim Cave are selected here as both are analysed with exactly the same methodology by the same analyst and are thus directly comparable. Formalising this assessment into a widely applicable comparative protocol is a highly desirable task for the future.

An additional factor related to the cost-efficiency of carinated reduction is the portability and versatility of the core, which increases strongly with the introduction of carinated reduction [33]. In the Aurignacian, one observes the mobility of cores, evidenced in different discard patterns and greater raw material variability. The use of small, versatile cores for the production of projectile implements is additionally a method to reduce raw material acquisition costs, as carinated reduction has a higher yield in target products and offers a more efficient use of raw material volume through the exploitation of large flakes. A full analysis of the Aurignacian inventories is required to understand potential differences in the treatment and transport of carinated items *vs*. the other reduction sequences and remains a task for the future. If the carinated cores should prove to show a higher transport frequency than the non-carinated cores, the question of portability would be solved.

### Use

To date no comprehensive study has been done on the use and hafting mechanics of small, retouched Aurignacian bladelets (of Dufour *et alii* types), therefore all discussion of possible uses and benefits remain in the realm of speculation or suggestions (read ‘working hypothesis’). The same goes for the interpretation of the preceding Ahmarian el-Wad points in addition to the question of their sporadic appearance in the Levantine Aurignacian (six are present in Hayonim Cave D; [⁠9]). The only use-wear study ever conducted that included el-Wad points from the Southern Ahmarian resulted in assigning them as drilling items [85], yet it is hard to believe this is the only or even the major use for these items. A study on the Ksar Akil points from the Northern Ahmarian concludes on a projectile use [93], while another suggests a function as spear thrower tips [94].

Aurignacian bladelets and el-Wad points are the major focus of the carinated and narrow-fronted reduction concepts. With general common sense and in allegory to preceding and following timeframes, the central products of the main reduction sequence of a Palaeolithic culture (until the Neolithic and the dawn of sickle blades) are usually connected to their use in hunting weapons. This would make sense under the notion that securing the next meal could justly be considered the most relevant task for hunter-gatherer groups and thus the focus of their tool making activity. We therefore interpret both tool types also as most probably projectile armament.

**Fig 24:**
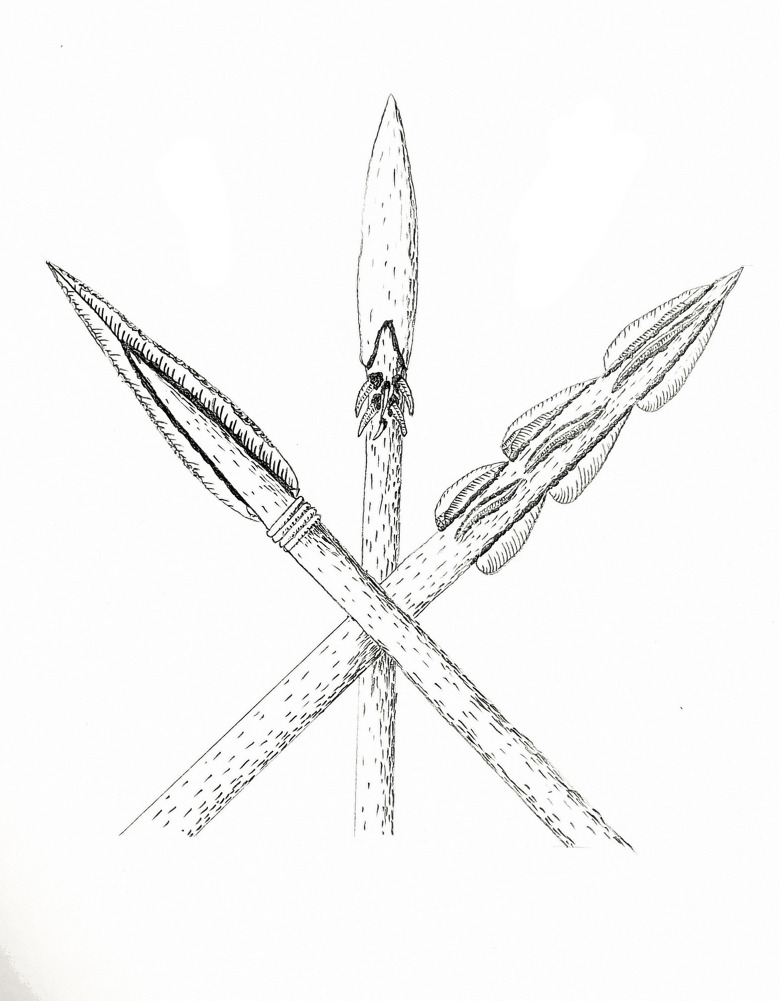
Illustrative comparison of different projectile hafting options. El Wad point (left), split based bone point (centre) and twisted bladelets (right) and their possible ways of hafting.

If the straight and pointed el-Wad point and the small twisted bladelets are compared to each other, it makes sense to assume that the el-Wad points were hafted centrally along the main axis of the blade(let) and the twisted bladelets were hafted laterally, most likely in a composite manner (Fig 24). If both are indeed projectile armaments, it is easily conceivable that they impact the balance of the resulting projectile differently given their different weight. As the balance is one of the most crucial aspects of a flying projectile, it can be assumed to be of importance to the makers. The twisted bladelets are much smaller, so their impact on the balance distribution of the projectile will be much reduced, which might give them a distinct advantage if the projectile is meant to fly farther afield, and balance becomes proportionally more important as range increases.

Even with the highly sophisticated blade core concept of the Southern Ahmarian, variations in the shape and weight of the blade(let) can occur, each of which would alter the balance of the projectile. The use of small bladelets in composite hafting allows for far greater control of the projectile balance, both in terms of the lighter weight of the bladelets as well as the variability allowed for how many and where to place them on the projectile.

It might thus be suggested that the introduction of carinated items is related to substantial changes in projectile design and projectile requirements. The new projectiles might have a better balance control and thus can reach far greater ranges than before. Obviously, it is very difficult to prove the use of a specific weapon technology, but it can be suggested that this change might be rooted in the development of a totally new lever technology–the spear thrower or atlatl. The earliest known examples of the atlatl are from late UP in Europe [95–97], yet there these items already show great sophistication and artistic decorations suggesting both, long familiarity with the technology and a certain value of the item. The earliest spear throwers might easily have been wooden sticks with a cut-off forked branch or an artificially created hook, neither of which would be preserved in archaeological context.

As a side note, naturally the role and place of bone points needs to be explained in this suggested hypothesis. While it is an entirely different object type, it might be proposed that these have an additional, parallel, or complementary function, maybe connected to different prey types, or different types of impact/penetration needed.

While the above is but a working hypothesis and only one possible explanation for the success of carinated items and composite technology as a whole, we hope that there will be future studies that will be able to provide evidence, for or against our hypothesis.

### Social learning and innovation transmission

If carinated items are indeed an innovation made in the context of the emergence of the Aurignacian and transferred into or onto the Levant, understanding processes of learning and adaptation of new ideas is vital in researching such an innovation transfer. The basic notion here would be the invention of carinated technology alongside the development of the Aurignacian techno-complex. It appears around 42.5ka in Europe [21] as a fully formed techno-complex which spreads throughout mainland Europe. This spread is hypothesised to reach the Levant around 37ka, where the Levantine Aurignacian inventories are interpreted as intrusive to the local groups and comparable to the European ones. Carinated items occur also in assemblages postdating the Aurignacian, suggesting the potential of an innovation transfer from the intrusive Aurignacians onto the local Levantine population [33].

Human Behavioural Ecology has made significant progress in recent years explaining dynamics of innovation transmission and acceptance [98]. An innovation is generally regarded as the implementation of an invention, i.e. the process of spreading and utilizing a novel idea or technology [99–101]. Inventions and innovations have to be studied separately [102], albeit the moment of invention often stays hidden in the archaeological record [102, 103]. The major focus for archaeology thus lies on innovation and its spread, which depends on two main preconditions: the information flow itself and its acceptance and implementation [104]. In general terms, this divides the population into innovators and imitators [105]. Within a society, innovations are usually spread through diffusion following an s-shaped adoption curve in stages of social acceptance dependent on the group individuals as 1) innovators, 2) early adopters, 3) early majority, 4) late majority, 5) laggards [106].

Evolutionary fitness in human social systems has been argued to be mainly defined by information transmission [56]. A number of different processes, preconditions and biases have been identified in the behavioural ecological literature to identify from whom to learn and what to learn [100, 107, 108]. Two main factors are relevant for the discussion of the adaptation of carinated technology by Levantine foragers:

Need has often been called the ‘mother of invention’ [109], implicitly or explicitly stating that a novel trait or technology would have to be *necessary* successful. However, this notion has been rejected repeatedly [99, 100, 110, 111] on the understanding that groups or societies under pressure rarely find the capacities to indulge in costly endeavours of developing or testing new inventions [100, 111]. Actually, lack of existential pressure encourages the spread of inventions as it permits ‘trial and error’ and costly mistakes [100]. This notion is connected to the discussion on the adoption of carinated technology as the rich but mosaic Late Pleistocene Levantine environment [33] reduces subsistence stress on the local population, and, together with a forager mode of resource exploitation, increases adaptive behaviour. The discussion about innovation transmission includes the notion of possible ‘biases’ acting on the process of transmission. One such bias is the ‘indirect bias’ which requires differences between the innovators and imitators and will act most strongly when opposing social models are encountered on the boundaries between groups practicing contrasting adaptive strategies [112, 113]. The hypothesised cultural contact between northern European Aurignacians, organised in logistical settlement systems [114] and local Levantine residential foragers [33, 40, 58], would fit the above and thus lead to increased variation and transmission of behavioural traits.

The second factor is connected to the perceived novelty of the innovation–carinated technology–in contrast to the locally practiced behaviour–the narrow-fronted blade technology of the Ahmarian. Often inventions are not fundamentally new but rather re-combinations or extensions of existing ideas or technologies [100]. The rate of adoption depends on the relative advantage of the new idea, on its compatibility with social values and established ideas as well as with current needs. It also depends on the complexity of the new idea or practice, on the potential for personally experimenting with the novelty, and on the extent to which the results of an innovative behaviour can be observed [103]. Development of technology is a cumulative process using existing building blocks to support new ones [115]. The Levantine Ahmarian practices a very straightforward, sophisticated but limited operational chain for blade/bladelet production from narrow-fronted cores [26, 28, 29, 33, 116] targeting the exploitation of the central face of the core with lateral, often twisted corrective blades/bladelets used to preserve the features of the reduction surface. A carinated core-on-blank, especially a carinated burin, follows the same conceptual logic, albeit on a different core support–a thick blade or flake instead of a nodule–of smaller size aimed to produce twisted lateralized items [33]. Only a small conceptual alteration is thus necessary for the understanding and adoption of the carinated reduction concept in the light of the narrow-fronted reduction, facilitating the quick spread and adoption of a carinated technology.

Identifying the technological characteristics, the conceptual knowledge, and the behavioural and technological repertoire of the European and the Levantine Aurignacian has the potential of tracing the transfer of a definable and specific technological design–carinated technology–and identifying processes of learning a new technology.

## Conclusion and outlook

In conclusion it can be said that there is no reason to doubt that carinated items in the Levant had at the very least a partial core-function. This challenges future endeavours to define carinated items in the Levant and their technological description. If it is accepted that they are bladelet cores, they can be considered as yet another core type, where the technological setup and aim should be considered the defining factors. It might thus be useful to drop the notion of ‘carinated burins and endscrapers’ and instead use the term ‘carinated cores’ differentiated into ‘burin-like’, ‘endscraper-like’ or any other setup.

Only future projects will enable a full understanding of the connection between the introduction of carinated items and changes in projectile type and mechanics, the specific transmission processes of the carinated invention as well as the associations between Levantine and European assemblages. One last interesting notion is the idea of Palaeolithic people actively gaining information from abandoned sites and being able to ‘read’ older flints, just like archaeologists do today. This method of information transmission can indeed be instrumental in the retention of a conceptual repertoire and might easily facilitate a flexible use, re-use, or adaptation of different technologies in time and space.

## Supporting information

S1 File Additional data tables. (DOCX)

S2 File Type list. (DOCX)

S3 File Attributes recorded in the analysis. (DOCX)

S4 File Core and Blank sizes. (XLSX)

S5 File Dataset used in the analysis. (XLSX)
