# Supplementary material for: The use of carinated items in the Levantine Aurignacian—Insights from layer D, Hayonim Cave, W. Galilee, Israel
Source: PLoS One. 2024 Jul 24;19(7):e0301102. doi: 10.1371/journal.pone.0301102 (PMC11268594; doi:10.1371/journal.pone.0301102)
Supplement: S1 File — (DOCX) [file pone.0301102.s001.docx]

**S1) Additional data tables.**

**S1 Table 1. Reduction surface lateralisation in carinated ‘endscrapers’.**

|  |  |  | **right** | **left** | **straight** | **indet.** | **total** |
| --- | --- | --- | --- | --- | --- | --- | --- |
| **short** | carinated | D1–2 | 14 | 3 | 9 | 1 | 27 |
|  |  | D3 | 10 | 3 | 6 |  | 19 |
|  |  | D4 |  |  |  |  | 0 |
|  |  | Total | 24 | 6 | 15 | 1 | 46 |
|  | nosed | D1/2 | 3 |  | 3 |  | 6 |
|  |  | D3 | 3 |  |  |  | 3 |
|  |  | D4 |  | 1 | 1 |  | 2 |
|  |  | Total | 6 | 1 | 4 | 0 | 11 |
| **long** | carinated | D1/2 |  |  | 2 |  | 2 |
|  |  | D3 | 1 |  | 4 |  | 5 |
|  |  | D4 |  |  | 2 |  | 2 |
|  |  | Total | 1 | 0 | 8 | 0 | 9 |
|  | nosed | D1/2 |  | 2 |  |  | 2 |
|  |  | D3 |  | 1 | 1 |  | 2 |
|  |  | D4 |  |  | 1 |  | 1 |
|  |  | Total | 0 | 3 | 2 | 0 | 5 |
|  | Total | D1/2 | 17 | 5 | 14 | 1 | 37 |
|  |  | D3 | 14 | 4 | 11 | 0 | 29 |
|  |  | D4 | 0 | 1 | 4 | 0 | 5 |
|  |  | Total | 31 | 10 | 29 | 1 | 71 |

**S1 Table 2. Shape reduction surface of carinated ‘endscrapers’.**

|  |  |  | **converging** | **subparallel** | **twisted** | **other** | **total** |
| --- | --- | --- | --- | --- | --- | --- | --- |
| **short** | carinated | D1/2 | 21 | 1 | 3 | 2 | 27 |
|  |  | D3 | 15 | 3 | 1 |  | 19 |
|  |  | D4 |  |  |  |  | 0 |
|  |  | Total | 36 | 4 | 4 | 2 | 46 |
|  | nosed | D1/2 | 5 |  | 1 |  | 6 |
|  |  | D3 | 3 |  |  |  | 3 |
|  |  | D4 | 1 |  |  | 1 | 2 |
|  |  | Total | 9 | 0 | 1 | 1 | 11 |
| **long** | carinated | D1/2 | 1 | 1 |  |  | 2 |
|  |  | D3 | 1 | 2 | 1 | 1 | 5 |
|  |  | D4 |  | 2 |  |  | 2 |
|  |  | Total | 2 | 5 | 1 | 1 | 9 |
|  | nosed | D1/2 | 2 |  |  |  | 2 |
|  |  | D3 | 2 |  |  |  | 2 |
|  |  | D4 | 1 |  |  |  | 1 |
|  |  | Total | 5 | 0 | 0 | 0 | 5 |
|  | Total | D1/2 | 29 | 2 | 4 | 2 | 37 |
|  |  | D3 | 21 | 5 | 2 | 1 | 29 |
|  |  | D4 | 2 | 2 | 0 | 1 | 5 |
|  |  | Total | 52 | 9 | 6 | 4 | 71 |

**S1 Table 3. Location of the carination in carinated ‘endscraper’s.**

|  |  |  | **distal** | **proximal** | **left lateral** | **right lateral** | **off angle/** | **indet.** | **total** |
| --- | --- | --- | --- | --- | --- | --- | --- | --- | --- |
|  |  |  |  |  |  |  | **transverse** |  |  |
| **short** | carinated | D1/2 | 22 | 4 | 1 |  |  |  | 27 |
|  |  | D3 | 10 | 5 | 1 | 1 |  | 2 | 19 |
|  |  | D4 |  |  |  |  |  |  | 0 |
|  |  | Total | 32 | 9 | 2 | 1 | 0 | 2 | 46 |
|  | nosed | D1/2 | 3 | 3 |  |  |  |  | 6 |
|  |  | D3 | 3 |  |  |  |  |  | 3 |
|  |  | D4 | 2 |  |  |  |  |  | 2 |
|  |  | Total | 8 | 3 | 0 | 0 | 0 | 0 | 11 |
| **long** | carinated | D1/2 | 1 |  |  |  | 1 |  | 2 |
|  |  | D3 | 2 | 2 |  |  | 1 |  | 5 |
|  |  | D4 | 2 |  |  |  |  |  | 2 |
|  |  | Total | 5 | 2 | 0 | 0 | 2 | 0 | 9 |
|  | nosed | D1/2 | 1 |  | 1 |  |  |  | 2 |
|  |  | D3 | 1 | 1 |  |  |  |  | 2 |
|  |  | D4 | 1 |  |  |  |  |  | 1 |
|  |  | Total | 3 | 1 | 1 | 0 | 0 | 0 | 5 |
|  | Total | D1/2 | 27 | 7 | 2 | 0 | 1 | 0 | 37 |
|  |  | D3 | 16 | 8 | 1 | 1 | 1 | 2 | 29 |
|  |  | D4 | 5 | 0 | 0 | 0 | 0 | 0 | 5 |
|  |  | Total | 48 | 15 | 3 | 1 | 2 | 2 | 71 |

**S1 Table 4. Preparation of carinated ‘endscrapers’.**

|  |  |  | **lateral** | **platform** | **distal preparation** | **not present** | **total** |
| --- | --- | --- | --- | --- | --- | --- | --- |
| **short** | carinated | D1/2 | 22 | 1 | 0 | 4 | 27 |
|  |  | D3 | 18 | 1 |  |  | 19 |
|  |  | D4 | 0 |  |  |  | 0 |
|  |  | Total | 40 | 2 | 0 | 4 | 46 |
|  | nosed | D1/2 | 6 |  |  |  | 6 |
|  |  | D3 | 3 |  |  |  | 3 |
|  |  | D4 | 2 |  |  |  | 2 |
|  |  | Total | 11 | 0 | 0 | 0 | 11 |
| **long** | carinated | D1/2 | 2 |  |  |  | 2 |
|  |  | D3 | 3 |  |  | 2 | 5 |
|  |  | D4 | 1 |  |  | 1 | 2 |
|  |  | Total | 6 | 0 | 0 | 3 | 9 |
|  | nosed | D1/2 | 2 |  |  |  | 2 |
|  |  | D3 | 1 |  | 1 |  | 2 |
|  |  | D4 | 1 |  |  |  | 1 |
|  |  | Total | 4 | 0 | 1 | 0 | 5 |
|  | Total | D1/2 | 32 | 1 | 0 | 4 | 37 |
|  |  | D3 | 25 | 1 | 1 | 2 | 29 |
|  |  | D4 | 4 | 0 | 0 | 1 | 5 |
|  |  | Total | 61 | 2 | 1 | 7 | 71 |

**S1 Table 5. Fracture termination of the last negative.**

|  |  |  | **flat** | **hinge** | **Indet.** | **total** |
| --- | --- | --- | --- | --- | --- | --- |
| **short** | carinated | D1/2 | 13 | 11 | 3 | 27 |
|  |  | D3 | 9 | 9 | 1 | 19 |
|  |  | D4 |  |  |  | 0 |
|  |  | Total | 22 | 20 | 4 | 46 |
|  | nosed | D1/2 | 3 | 2 | 1 | 6 |
|  |  | D3 | 1 | 2 |  | 3 |
|  |  | D4 |  | 1 | 1 | 2 |
|  |  | Total | 4 | 5 | 2 | 11 |
| **long** | carinated | D1/2 | 1 | 1 |  | 2 |
|  |  | D3 | 4 | 1 |  | 5 |
|  |  | D4 |  | 2 |  | 2 |
|  |  | Total | 5 | 4 | 0 | 9 |
|  | nosed | D1/2 |  | 2 |  | 2 |
|  |  | D3 | 2 |  |  | 2 |
|  |  | D4 |  | 1 |  | 1 |
|  |  | Total | 2 | 3 | 0 | 5 |
|  | Total | D1/2 | 17 | 16 | 4 | 37 |
|  |  | D3 | 16 | 12 | 1 | 29 |
|  |  | D4 | 0 | 4 | 1 | 5 |
|  |  | Total | 33 | 32 | 6 | 71 |

**S1 Table 6. Blank types of Carinated burins.**

|  | **D1/2** | **D3** | **D4** | **Total** |
| --- | --- | --- | --- | --- |
| **blade** | 3 | 1 | 0 | 4 |
| **flake** | 11 | 16 | 2 | 29 |
| **Indet. frgm.** | 6 | 4 | 2 | 12 |
| **Total** | 20 | 21 | 4 | 45 |

**S1 Table 7. Location of the carination in relation to knapping direction of the blank.**

|  | **D1/2** | **D3** | **D4** | **Total** |
| --- | --- | --- | --- | --- |
| **distal** | 6 | 11 | 2 | 19 |
| **right lateral** | 2 | 4 |  | 6 |
| **left lateral** | 3 | 3 | 3 | 9 |
| **off angle / transverse** | 4 |  |  | 4 |
| **proximal** | 8 | 4 | 1 | 13 |
| **Total** | 23 | 22 | 6 | 51 |

**S1 Table 8. Location of preparation evidence on carinated ‘burins’.**

|  | **D1/2** | **D3** | **D4** | **Total** |
| --- | --- | --- | --- | --- |
| **distal** | 2 |  | 2 | 4 |
| **distal + back** | 1 |  |  | 1 |
| **platform** | 9 | 6 | 2 | 17 |
| **platform + distal** | 5 | 9 | 1 | 15 |
| **platform + distal + back** | 2 | 2 |  | 4 |
| **not present** | 4 | 5 | 1 | 10 |
| **Total** | 23 | 22 | 6 | 51 |

**S1 Table 9. Striking platform preparation of carinated ‘burins’.**

|  | **D1/2** | **D3** | **D4** | **Total** |
| --- | --- | --- | --- | --- |
| **retouch** | 2 | 4 | 1 | 7 |
| **tablet** | 15 | 8 | 3 | 26 |
| **tablet + retouch** | 3 | 2 |  | 5 |
| **multiple tablets** |  | 2 |  | 2 |
| **multiple tablets + retouch** |  | 2 |  | 2 |
| **natural/cortical** | 1 | 1 |  | 2 |
| **none** | 2 | 3 | 2 | 7 |
| **Total** | 23 | 22 | 6 | 51 |

**S1 Table 10. Reduction surface lateralisation of carinated ‘burins’.**

|  | **D1/2** | **D3** | **D4** | **Total** |
| --- | --- | --- | --- | --- |
| **dorsal right-sided** | 1 | 2 |  | 3 |
| **straight** | 12 | 9 | 1 | 22 |
| **ventral right-sided** | 8 | 8 | 2 | 18 |
| **ventral left-sided** | 1 | 3 | 1 | 5 |
| **Indet.** | 1 |  | 2 | 3 |
| **Total** | 23 | 22 | 6 | 51 |

**S1 Table 11. Shape of the reduction surface.**

| **Shape RS** | **D1/2** | **D3** | **D4** | **Total** |
| --- | --- | --- | --- | --- |
| **subparallel** | 16 | 10 | 3 | 29 |
| **converging** | 4 | 9 | 2 | 15 |
| **twisted** | 2 | 2 | 1 | 5 |
| **other** | 1 | 1 |  | 2 |
| **Total** | 23 | 22 | 6 | 51 |

**S1 Table 12. Fracture termination of the last negative.**

|  | **D1/2** | **D3** | **D4** | **Total** |
| --- | --- | --- | --- | --- |
| **flat** | 10 | 12 | 2 | 24 |
| **hinge** | 12 | 9 | 4 | 25 |
| **Indet.** | 1 | 1 |  | 2 |
| **Total** | 23 | 22 | 6 | 51 |

**S1 Table 13. Hayonim D Bladelets analysed in the attribute analysis.**

|  | **D1/2** | **D3** | **D4** | **Total** |
| --- | --- | --- | --- | --- |
| **Initial Opening and decortication** | 16 | 20 | 6 | 42 |
| **Preparation and Maintenance** | 21 | 17 | 8 | 46 |
| **Reduction target** | 46 | 72 | 10 | 128 |
| **Reduction by-product** | 89 | 72 | 32 | 193 |
| **Reduction total** | 135 | 144 | 42 | 321 |
| **Tool** | 54 | 51 | 6 | 111 |
| **Total** | 226 | 232 | 62 | 520 |

**S1 Table 14. Cortex preservation.**

|  | D1-2 |  |  | D3 |  |  | D4 |  |  | Total |  |  | Total |
| --- | --- | --- | --- | --- | --- | --- | --- | --- | --- | --- | --- | --- | --- |
| Cortex | tool | target | by-product | tool | target | by-product | tool | target | by-product | tool | target | by-product |  |
| none | 47 | 44 | 57 | 46 | 68 | 51 | 6 | 9 | 23 | 99 | 121 | 131 | 351 |
| lateral | 1 | 1 | 16 | 1 |  | 8 |  |  | 3 | 2 | 1 | 27 | 30 |
| distal | 3 | 1 | 9 | 3 | 2 | 7 |  | 1 | 5 | 6 | 4 | 21 | 31 |
| proximal |  |  | 3 |  | 2 | 4 |  |  |  | 0 | 2 | 7 | 9 |
| lateral+ distal | 3 |  | 2 | 1 |  | 2 |  |  |  | 4 | 0 | 4 | 8 |
| medial |  |  |  |  |  |  |  |  | 1 | 0 | 0 | 1 | 1 |
| distal+ proximal |  |  | 1 |  |  |  |  |  |  | 0 | 0 | 1 | 1 |
| lateral+ distal+ proximal |  |  |  |  |  |  |  |  |  | 0 | 0 | 0 | 0 |
| lateral+ medial |  |  |  |  |  |  |  |  |  | 0 | 0 | 0 | 0 |
| bilateral | 0 |  | 1 |  |  |  |  |  |  | 0 | 0 | 1 | 1 |
| Total | 54 | 46 | 89 | 51 | 72 | 72 | 6 | 10 | 32 | 111 | 128 | 193 | 432 |

**S1 Table 15. Blank preservation.**

|  | D1-2 |  |  | D3 |  |  | D4 |  |  | Total |  |  | Total |
| --- | --- | --- | --- | --- | --- | --- | --- | --- | --- | --- | --- | --- | --- |
| blank preservation | tool | target | by-product | tool | target | by-product | tool | target | by-product | tool | target | by-product |  |
| complete | 28 | 27 | 57 | 23 | 44 | 46 | 1 | 8 | 21 | 52 | 79 | 124 | 255 |
| distal | 5 | 7 | 4 |  | 5 | 5 |  | 1 |  | 5 | 13 | 9 | 27 |
| proximal | 4 |  | 2 | 2 | 2 | 3 |  |  | 3 | 6 | 2 | 8 | 16 |
| medial | 5 | 3 | 6 | 8 | 2 | 4 | 1 |  | 2 | 14 | 5 | 12 | 31 |
| prox+ med | 8 | 4 | 10 | 13 | 13 | 8 | 3 |  | 2 | 24 | 17 | 20 | 61 |
| dist+ med | 4 | 5 | 10 | 5 | 6 | 6 | 1 | 1 | 4 | 10 | 12 | 20 | 42 |
| Total | 54 | 46 | 89 | 51 | 72 | 72 | 6 | 10 | 32 | 111 | 128 | 193 | 432 |

**S1 Table 16. Platform shape.**

|  | D1-2 |  |  | D3 |  |  | D4 |  |  | Total |  |  | Total |
| --- | --- | --- | --- | --- | --- | --- | --- | --- | --- | --- | --- | --- | --- |
| PF shape | tool | target | by-product | tool | target | by-product | tool | target | by-product | tool | target | by-product |  |
| plain | 7 | 6 | 16 | 19 | 9 | 17 | 1 | 1 | 9 | 27 | 16 | 42 | 85 |
| primary faceted |  |  |  |  | 1 |  |  |  |  | 0 | 1 | 0 | 1 |
| linear/ridge | 12 | 12 | 20 | 6 | 21 | 16 |  | 2 | 3 | 18 | 35 | 39 | 92 |
| punctiform | 20 | 13 | 26 | 13 | 27 | 18 | 2 | 5 | 13 | 35 | 45 | 57 | 137 |
| splintered | 1 |  | 7 |  | 1 | 6 | 1 |  | 1 | 2 | 1 | 14 | 17 |
| not present | 14 | 15 | 20 | 13 | 13 | 15 | 2 | 2 | 6 | 29 | 30 | 41 | 100 |
| Total | 54 | 46 | 89 | 51 | 72 | 72 | 6 | 10 | 32 | 111 | 128 | 193 | 432 |

**S1 Table 17. Bulb.**

|  | D1-2 |  |  | D3 |  |  | D4 |  |  | Total |  |  | Total |
| --- | --- | --- | --- | --- | --- | --- | --- | --- | --- | --- | --- | --- | --- |
| Bulb | tool | target | by-product | tool | target | by-product | tool | target | by-product | tool | target | by-product |  |
| developed | 6 | 2 | 9 | 1 |  | 5 |  |  | 3 | 7 | 2 | 17 | 26 |
| diffuse | 32 | 29 | 49 | 37 | 58 | 50 | 4 | 8 | 22 | 73 | 95 | 121 | 289 |
| Hertzian cone |  |  | 1 |  |  | 1 |  |  |  | 0 | 0 | 2 | 2 |
| ridge | 2 |  |  |  |  | 1 |  |  |  | 2 | 0 | 1 | 3 |
| not present |  |  | 10 |  | 1 |  |  |  | 1 | 0 | 1 | 11 | 12 |
| Prox. end not pres. | 14 | 15 | 20 | 13 | 13 | 15 | 2 | 2 | 6 | 29 | 30 | 41 | 100 |
| Total | 54 | 46 | 89 | 51 | 72 | 72 | 6 | 10 | 32 | 111 | 128 | 193 | 432 |

**S1 Table 18. Scar.**

|  | D1-2 |  |  | D3 |  |  | D4 |  |  | Total |  |  | Total |
| --- | --- | --- | --- | --- | --- | --- | --- | --- | --- | --- | --- | --- | --- |
| Scar | tool | target | by-product | tool | target | by-product | tool | target | by-product | tool | target | by-product |  |
| Prox. end n. pres. | 14 | 15 | 20 | 13 | 13 | 15 | 2 | 2 | 6 | 29 | 30 | 41 | 100 |
| transverse |  |  | 1 |  |  |  |  |  |  | 0 | 0 | 1 | 1 |
| aligned | 11 | 9 | 17 | 5 | 11 | 14 | 1 | 1 | 8 | 17 | 21 | 39 | 77 |
| not present | 29 | 22 | 51 | 33 | 48 | 43 | 3 | 7 | 18 | 65 | 77 | 112 | 254 |
| Total | 54 | 46 | 89 | 51 | 72 | 72 | 6 | 10 | 32 | 111 | 128 | 193 | 432 |

**S1 Table 19. Flake Lip.**

|  | D1-2 | |  | D3 |  |  | D4 |  |  | Total |  |  | Total |
| --- | --- | --- | --- | --- | --- | --- | --- | --- | --- | --- | --- | --- | --- |
| Lip | tool | target | by-product | tool | target | by-product | tool | target | by-product | tool | target | by-product |  |
| Prox. end n. pres. | 14 | 15 | 20 | 13 | 13 | 15 | 2 | 2 | 6 | 29 | 30 | 41 | 100 |
| preserved | 32 | 28 | 53 | 37 | 56 | 45 | 3 | 8 | 24 | 72 | 92 | 122 | 286 |
| not preserved | 8 | 3 | 16 | 1 | 3 | 12 | 1 |  | 2 | 10 | 6 | 30 | 46 |
| Total | 54 | 46 | 89 | 51 | 72 | 72 | 6 | 10 | 32 | 111 | 128 | 193 | 432 |

**S1 Table 20. Abrasion.**

|  | D1-2 |  |  | D3 |  |  | D4 |  |  | Total |  |  | Total |
| --- | --- | --- | --- | --- | --- | --- | --- | --- | --- | --- | --- | --- | --- |
| Abrasion | tool | target | by-product | tool | target | by-product | tool | target | by-product | tool | target | by-product |  |
| yes | 34 | 31 | 47 | 36 | 51 | 45 | 4 | 7 | 21 | 74 | 89 | 113 | 276 |
| no | 20 | 15 | 42 | 15 | 21 | 27 | 2 | 3 | 11 | 37 | 39 | 80 | 156 |
| Total | 54 | 46 | 89 | 51 | 72 | 72 | 6 | 10 | 32 | 111 | 128 | 193 | 432 |

**S1 Table 21. Knapping angle.**

|  | D1-2 |  |  | D3 |  |  | D4 |  |  | Total |  |  | Total |
| --- | --- | --- | --- | --- | --- | --- | --- | --- | --- | --- | --- | --- | --- |
| Knapp Angle | tool | target | by-product | tool | target | by-product | tool | target | by-product | tool | target | by-product |  |
| acute | 38 | 29 | 61 | 38 | 57 | 50 | 4 | 8 | 24 | 80 | 94 | 135 | 309 |
| 90° |  | 2 | 7 |  | 2 | 5 |  |  | 2 | 0 | 4 | 14 | 18 |
| indet | 2 |  | 1 |  |  | 2 |  |  |  | 2 | 0 | 3 | 5 |
| Prox. end n. pres. | 14 | 15 | 20 | 13 | 13 | 15 | 2 | 2 | 6 | 29 | 30 | 41 | 100 |
| Total | 54 | 46 | 89 | 51 | 72 | 72 | 6 | 10 | 32 | 111 | 128 | 193 | 432 |

**S1 Table 22. Longitudinal section.**

|  | D1-2 |  |  | D3 |  |  | D4 |  |  | Total |  |  | Total |
| --- | --- | --- | --- | --- | --- | --- | --- | --- | --- | --- | --- | --- | --- |
| Long Sec | tool | target | by-product | tool | target | by-product | tool | target | by-product | tool | target | by-product |  |
| straight | 13 | 11 | 35 | 9 | 23 | 36 | 1 | 3 | 14 | 23 | 37 | 85 | 145 |
| curved smooth | 16 | 19 | 26 | 30 | 34 | 14 | 2 | 7 | 7 | 48 | 60 | 47 | 155 |
| curved prox. | 5 | 3 | 2 | 1 | 1 | 1 |  |  | 1 | 6 | 4 | 4 | 14 |
| curved med | 2 | 1 | 2 | 2 | 3 | 3 | 1 |  |  | 5 | 4 | 5 | 14 |
| curved dist. | 8 | 3 | 12 | 3 | 2 | 11 | 1 |  | 6 | 12 | 5 | 29 | 46 |
| indet | 10 | 9 | 12 | 6 | 9 | 7 | 1 |  | 4 | 17 | 18 | 23 | 58 |
| Total | 54 | 46 | 89 | 51 | 72 | 72 | 6 | 10 | 32 | 111 | 128 | 193 | 432 |

**S1 Table 23. Cross section.**

|  | D1-2 |  |  | D3 |  |  | D4 |  |  | Total |  |  | Total |
| --- | --- | --- | --- | --- | --- | --- | --- | --- | --- | --- | --- | --- | --- |
| cross-sec | tool | target | by-product | tool | target | by-product | tool | target | by-product | tool | target | by-product |  |
| equilateral triangular | 1 |  | 2 | 1 |  | 2 | 1 |  |  | 3 | 0 | 4 | 7 |
| flat triangular lateralised | 4 |  | 10 | 8 | 8 | 21 |  | 1 | 6 | 12 | 9 | 37 | 58 |
| flat triangular central | 26 | 24 | 41 | 26 | 36 | 24 | 3 | 7 | 15 | 55 | 67 | 80 | 202 |
| trapezoidal central | 21 | 21 | 30 | 16 | 27 | 19 | 2 | 2 | 8 | 39 | 50 | 57 | 146 |
| trapezoidal lateralised | 2 | 1 | 6 |  | 1 | 4 |  |  | 3 | 2 | 2 | 13 | 17 |
| rectangular |  |  |  |  |  | 2 |  |  |  | 0 | 0 | 2 | 2 |
| Total | 54 | 46 | 89 | 51 | 72 | 72 | 6 | 10 | 32 | 111 | 128 | 193 | 432 |

**S1 Table 24. Twisting.**

|  | D1-2 |  |  | D3 |  |  | D4 |  |  | Total |  |  | Total |
| --- | --- | --- | --- | --- | --- | --- | --- | --- | --- | --- | --- | --- | --- |
| twist | tool | target | by-product | tool | target | by-product | tool | target | by-product | tool | target | by-product |  |
| right | 28 | 11 | 28 | 29 | 32 | 31 | 3 | 3 | 17 | 60 | 46 | 76 | 182 |
| left | 8 | 8 | 16 | 4 | 7 | 13 |  | 6 | 7 | 12 | 21 | 36 | 69 |
| straight | 13 | 25 | 40 | 16 | 31 | 26 | 2 | 1 | 7 | 31 | 57 | 73 | 161 |
| indet | 5 | 2 | 5 | 2 | 2 | 2 | 1 |  | 1 | 8 | 4 | 8 | 20 |
| Total | 54 | 46 | 89 | 51 | 72 | 72 | 6 | 10 | 32 | 111 | 128 | 193 | 432 |

**S1 Table 25. Presence of ventral remains.**

|  | D1-2 |  |  | D3 |  |  | D4 |  |  | Total |  |  | Total |
| --- | --- | --- | --- | --- | --- | --- | --- | --- | --- | --- | --- | --- | --- |
| ventral remains | tool | target | by-product | tool | target | by-product | tool | target | by-product | tool | target | by-product |  |
| yes | 5 | 0 | 8 | 2 | 2 | 12 | 1 | 1 | 4 | 8 | 3 | 24 | 35 |
| no | 49 | 46 | 81 | 49 | 70 | 60 | 5 | 9 | 28 | 103 | 125 | 169 | 397 |
| Total | 54 | 46 | 89 | 51 | 72 | 72 | 6 | 10 | 32 | 111 | 128 | 193 | 432 |

**S1 Table 26. Location of ventral remains.**

|  | D1-2 |  |  | D3 |  |  | D4 |  |  | Total |  |  | Total |
| --- | --- | --- | --- | --- | --- | --- | --- | --- | --- | --- | --- | --- | --- |
| Location ventral remains | tool | target | by-product | tool | target | by-product | tool | target | by-product | tool | target | by-product |  |
| right | 4 |  | 3 | 2 | 1 | 4 | 1 |  | 3 | 7 | 1 | 10 | 18 |
| left | 1 |  | 5 |  | 1 | 8 |  | 1 | 1 | 1 | 2 | 14 | 17 |
| not pres. | 49 | 46 | 81 | 49 | 70 | 60 | 5 | 9 | 28 | 103 | 125 | 169 | 397 |
| Total | 54 | 46 | 89 | 51 | 72 | 72 | 6 | 10 | 32 | 111 | 128 | 193 | 432 |

**S1 Table 27. Direction dorsal negatives.**

|  | D1-2 | |  | D3 |  |  | D4 |  |  | Total |  |  | Total |
| --- | --- | --- | --- | --- | --- | --- | --- | --- | --- | --- | --- | --- | --- |
| Dir. Dorsal negatives | tool | target | by-product | tool | target | by-product | tool | target | by-product | tool | target | by-product |  |
| aligned | 40 | 30 | 60 | 35 | 44 | 44 | 5 | 5 | 17 | 80 | 79 | 121 | 280 |
| mixed | 1 |  | 4 | 3 |  | 1 |  |  |  | 4 | 0 | 5 | 9 |
| aligned + cross | 4 | 1 | 8 | 1 | 2 | 6 |  | 1 | 7 | 5 | 4 | 21 | 30 |
| perpend + cross |  |  |  | 1 |  |  |  |  |  | 1 | 0 | 0 | 1 |
| mixed + cross |  |  |  |  |  | 2 |  |  |  | 0 | 0 | 2 | 2 |
| converging | 8 | 15 | 9 | 7 | 22 | 13 | 1 | 3 | 7 | 16 | 40 | 29 | 85 |
| oblique |  |  | 1 |  |  |  |  |  |  | 0 | 0 | 1 | 1 |
| indet | 1 |  | 2 |  | 1 | 1 |  |  |  | 1 | 1 | 3 | 5 |
| no dorsal negatives |  |  |  |  |  | 1 |  |  |  | 0 | 0 | 1 | 1 |
| converging+  aligned |  |  | 5 | 4 | 3 | 4 |  | 1 | 1 | 4 | 4 | 10 | 18 |
| Total | 54 | 46 | 89 | 51 | 72 | 72 | 6 | 10 | 32 | 111 | 128 | 193 | 432 |

**S1 Table 28. Fracture termination.**

|  | D1-2 | |  | D3 |  |  | D4 |  |  | Total |  |  | Total |
| --- | --- | --- | --- | --- | --- | --- | --- | --- | --- | --- | --- | --- | --- |
| fracture termination | tool | target | by-product | tool | target | by-product | tool | target | by-product | tool | target | by-product |  |
| not present | 17 | 5 | 16 | 20 | 16 | 13 | 4 |  | 7 | 41 | 21 | 36 | 98 |
| flat | 33 | 37 | 52 | 26 | 53 | 46 |  | 9 | 19 | 59 | 99 | 117 | 275 |
| hinge |  | 1 | 6 |  | 2 | 4 |  | 1 |  | 0 | 4 | 10 | 14 |
| overshot | 3 |  | 7 | 1 |  | 7 | 1 |  | 3 | 5 | 0 | 17 | 22 |
| thick | 1 | 3 | 8 | 4 | 1 | 2 |  |  | 3 | 5 | 4 | 13 | 22 |
| indet |  |  |  |  |  |  | 1 |  |  | 1 | 0 | 0 | 1 |
| Total | 54 | 46 | 89 | 51 | 72 | 72 | 6 | 10 | 32 | 111 | 128 | 193 | 432 |

**S1 Table 29. Preparation and maintenance product type in retouched bladelets.**

|  | **Type Number (see. Fig .5)** | **Retouched bladelets** |
| --- | --- | --- |
| **initially crested** | 1 | 1 |
| **plain** | 3 | 74 |
| **first order lateral** | 4 | 12 |
| **second order lateral** | 5 | 2 |
| **core** | 11 |  |
| **other** | 12 | 10 |
| **indet** | 13 | 5 |

**S1 Table 30. Location of retouch on Hayonim Cave D bladelets.**

|  | **D1/2** | | **D3** | | **D4** | | **Total** | |
| --- | --- | --- | --- | --- | --- | --- | --- | --- |
|  | n | % | n | % | n | % | n | % |
| **proximal** | 1 | 1.9 | 1 | 2 |  |  | 2 | 1.8 |
| **distal** | 4 | 7.4 | 7 | 13.7 | 1 | 16.7 | 12 | 10.8 |
| **continuous left dorsal** | 14 | 25.9 | 9 | 17.6 | 1 | 16.7 | 24 | 21.6 |
| **continuous right dorsal** | 4 | 7.4 | 1 | 2 | 2 | 33.3 | 7 | 6.3 |
| **discontinuous alternating** | 2 | 3.7 | 6 | 11.8 |  |  | 8 | 7.2 |
| **continuous left ventral** | 2 | 3.7 | 3 | 5.9 | 1 | 16.7 | 6 | 5.4 |
| **continuous right ventral** | 1 | 1.9 |  |  |  |  | 1 | 0.9 |
| **discontinuous left dorsal** | 10 | 18.5 | 8 | 15.7 |  |  | 18 | 16.2 |
| **discontinuous right dorsal** | 7 | 13 | 5 | 9.8 | 1 | 16.7 | 13 | 11.7 |
| **discontinuous alternating** |  |  | 2 | 3.9 |  |  | 2 | 1.8 |
| **discontinuous left ventral** | 3 | 5.6 | 1 | 2 |  |  | 4 | 3.6 |
| **continuous bilateral dorsal** | 1 | 1.9 | 6 | 11.8 |  |  | 7 | 6.3 |
| **discontinuous bilateral dorsal** | 3 | 5.6 | 2 | 3.9 |  |  | 5 | 4.5 |
| **left dorsal + ventral** | 1 | 1.9 |  |  |  |  | 1 | 0.9 |
| **right dorsal + ventral** | 1 | 1.9 |  |  |  |  | 1 | 0.9 |
| **Total** | 54 | 100 | 51 | 100 | 6 | 100 | 111 | 100 |

**S1 Table 31. Nodule selection of non-carinated cores.**

|  | **D1/2** | | **D3** | | **D4** | | **D** | | | **Total** | | |
| --- | --- | --- | --- | --- | --- | --- | --- | --- | --- | --- | --- | --- |
|  | n | % | n | % | n | % | n | % | n | | % |  |
| **indeterminable** | 25 | 25.8 | 27 | 35.1 | 4 | 12.5 | 3 | 30 | 59 | | 27.3 |  |
| **whole nodule** | 43 | 44.3 | 40 | 51.9 | 19 | 59.4 | 5 | 50 | 107 | | 49.5 |  |
| **tabular section** | 1 | 1 | 2 | 2.6 |  |  |  |  | 3 | | 1.4 |  |
| **irregular chunk** | 10 | 10.3 | 1 | 1.3 | 3 | 9.4 | 1 | 10 | 15 | | 6.9 |  |
| **flake** | 14 | 14.4 | 4 | 5.2 | 3 | 9.4 | 1 | 10 | 22 | | 10.2 |  |
| **intrusion** | 4 | 4.1 | 3 | 3.9 | 3 | 9.4 |  |  | 10 | | 4.6 |  |
| **Total** | 97 | 100 | 77 | 100 | 32 | 100 | 10 | 100 | 216 | | 100 |  |

**S1 Table 32. Preservation of natural surfaces on non-carinated cores.**

|  | **D1/2** | | **D3** | | **D4** | | **D** | | **Total** | |
| --- | --- | --- | --- | --- | --- | --- | --- | --- | --- | --- |
|  | n | % | n | % | n | % | n | % | n | % |
| **no cortex** | 18 | 18.6 | 8 | 10.4 | 1 | 3.1 |  |  | 27 | 12.5 |
| **fresh** | 58 | 59.8 | 48 | 62.3 | 23 | 71.9 | 7 | 70 | 136 | 63 |
| **Fluvial** | 1 | 1 |  |  |  |  |  |  | 1 | 0.5 |
| **battered** |  |  |  |  |  |  |  |  |  |  |
| **fresh and old surface** | 7 | 7.2 | 8 | 10.4 | 5 | 15.6 | 3 | 30 | 23 | 10.6 |
| **old surface** | 13 | 13.4 | 9 | 11.7 | 2 | 6.3 |  |  | 24 | 11.1 |
| **indeterminable** |  |  | 4 | 5.2 | 1 | 3.1 |  |  | 5 | 2.3 |
| **Total** | 97 | 100 | 77 | 100 | 32 | 100 | 10 | 100 | 216 | 100 |

**S1 Table. 33. Non-carinated core types.**

|  | **D1/2** | | **D3** | | **D4** | | **D** | | **Total** | |
| --- | --- | --- | --- | --- | --- | --- | --- | --- | --- | --- |
|  | n | % | n | % | n | % | n | % | n | % |
| **indeterminable** | 3 | 3.1 | 2 | 2.6 |  |  |  |  | 5 | 2.3 |
| **discoid** | 3 | 3.1 | 3 | 3.9 |  |  |  |  | 6 | 2.8 |
| **multiple** | 9 | 9.3 | 5 | 6.5 | 4 | 12.5 | 1 | 10 | 19 | 8.8 |
| **unidirectional** | 46 | 47.4 | 48 | 62.3 | 18 | 56.3 | 6 | 60 | 118 | 54.6 |
| **bidirectional** | 11 | 11.3 | 5 | 6.5 | 1 | 3.1 | 1 | 10 | 18 | 8.3 |
| **90° turned** | 2 | 2.1 | 2 | 2.6 | 1 | 3.1 | 1 | 10 | 6 | 2.8 |
| **opposite** | 11 | 11.3 | 3 | 3.9 |  |  |  |  | 14 | 6.5 |
| **tested** | 9 | 9.3 | 5 | 6.5 | 7 | 21.9 | 1 | 10 | 22 | 10.2 |
| **Levallois-like** | 3 | 3.1 | 4 | 5.2 | 1 | 3.1 |  |  | 8 | 3.7 |
| **Total** | 97 | 100 | 77 | 100 | 32 | 100 | 10 | 100 | 216 | 100 |

**S1 Table 34. Non-carinated core shapes.**

|  | **D1/2** | | **D3** | | **D4** | | **D** | | **Total** | |
| --- | --- | --- | --- | --- | --- | --- | --- | --- | --- | --- |
|  | n | % | n | % | n | % | n | % | n | % |
| **indeterminable** | 1 | 1 | 3 | 3.9 |  |  |  |  | 4 | 1.9 |
| **converging** | 17 | 17.5 | 16 | 20.8 | 2 | 6.3 | 4 | 40 | 39 | 18.1 |
| **parallel** | 43 | 44.3 | 35 | 45.5 | 14 | 43.8 | 3 | 30 | 95 | 44 |
| **amorphous** | 12 | 12.4 | 6 | 7.8 | 4 | 12.5 | 1 | 10 | 23 | 10.6 |
| **core-on-flake** | 8 | 8.2 | 2 | 2.6 |  |  | 1 | 10 | 11 | 5.1 |
| **centripetal** | 4 | 4.1 | 1 | 1.3 | 1 | 3.1 |  |  | 6 | 2.8 |
| **tested** | 6 | 6.2 | 5 | 6.5 | 7 | 21.9 | 1 | 10 | 19 | 8.8 |
| **endscraper-like** | 4 | 4.1 | 2 | 2.6 |  |  |  |  | 6 | 2.8 |
| **burin-like** | 2 | 2.1 | 3 | 3.9 | 3 | 9.4 |  |  | 8 | 3.7 |
| **narrow-fronted** |  |  | 4 | 5.2 | 1 | 3.1 |  |  | 5 | 2.3 |
| **Total** | 97 | 100 | 77 | 100 | 32 | 100 | 10 | 100 | 216 | 100 |

**S1 Table 35. Number of reduction surfaces and platforms on non-carinated cores.**

| No. of reduction surfaces | D1/2 | | D3 | | D4 | | D | | Total | |
| --- | --- | --- | --- | --- | --- | --- | --- | --- | --- | --- |
| a) | n | % | n | % | n | % | n | % | n | % |
| 1 | 74 | 76.3 | 67 | 87 | 25 | 78.1 | 7 | 70 | 173 | 80.1 |
| 2 | 13 | 13.4 | 6 | 7.8 | 3 | 9.4 | 2 | 20 | 24 | 11.1 |
| 3 | 7 | 7.2 | 4 | 5.2 | 1 | 3.1 | 1 | 10 | 13 | 6 |
| 4 | 3 | 3.1 |  |  | 3 | 9.4 |  |  | 6 | 2.8 |
| Total | 97 | 100 | 77 | 100 | 32 | 100 | 10 | 100 | 216 | 100 |
|  |  |  |  |  |  |  |  |  |  |  |
| No. of platforms | D1/2 | | D3 | | D4 | | D | | Total | |
| b) | n | % | n | % | n | % | n | % | n | % |
| 1 | 69 | 71.1 | 63 | 81.8 | 27 | 84.4 | 7 | 70 | 166 | 76.9 |
| 2 | 18 | 18.6 | 10 | 13 | 2 | 6.3 | 2 | 20 | 32 | 14.8 |
| 3 | 7 | 7.2 | 4 | 5.2 | 3 | 9.4 | 1 | 10 | 15 | 6.9 |
| 4 | 3 | 3.1 |  |  |  |  |  |  | 3 | 1.4 |
| Total | 97 | 100 | 77 | 100 | 32 | 100 | 10 | 100 | 216 | 100 |

**S1 Table 36. Burning, possible heat-treatment, breakage, and twisting of non-carinated cores.**

|  | D1/2 | | D3 | | D4 | | D | | Total | |
| --- | --- | --- | --- | --- | --- | --- | --- | --- | --- | --- |
|  | n | % | n | % | n | % | n | % | n | % |
| Burned |  |  |  |  |  |  |  |  |  |  |
| yes | 22 | 22.7 | 26 | 33.8 | 7 | 21.9 | 2 | 20 | 57 | 26.4 |
| no | 75 | 77.3 | 51 | 66.2 | 25 | 78.1 | 8 | 80 | 159 | 73.6 |
| Total | 97 | 100 | 77 | 100 | 32 | 100 | 10 | 100 | 216 | 100 |
| Possibly heat- treated |  |  |  |  |  |  |  |  |  |  |
| yes | 18 | 18.6 | 8 | 10.4 | 3 | 9.4 | 1 | 10 | 30 | 13.9 |
| no | 79 | 81.4 | 69 | 89.6 | 29 | 90.6 | 9 | 90 | 186 | 86.1 |
| Total | 97 | 100 | 77 | 100 | 32 | 100 | 10 | 100 | 216 | 100 |
| Broken |  |  |  |  |  |  |  |  |  |  |
| yes | 7 | 7.2 | 4 | 5.2 | 1 | 3.1 | 0 | 0 | 12 | 5.6 |
| no | 90 | 92.8 | 73 | 94.8 | 31 | 96.9 | 10 | 100 | 204 | 94.4 |
| Total | 97 | 100 | 77 | 100 | 32 | 100 | 10 | 100 | 216 | 100 |
| Twisted |  |  |  |  |  |  |  |  |  |  |
| yes | 16 | 16.5 | 9 | 11.7 | 1 | 3.1 | 1 | 10 | 27 | 12.5 |
| no | 81 | 83.5 | 68 | 88.3 | 31 | 96.9 | 9 | 90 | 189 | 87.5 |
| Total | 97 | 100 | 77 | 100 | 32 | 100 | 10 | 100 | 216 | 100 |
