# Supplementary material for: The use of carinated items in the Levantine Aurignacian—Insights from layer D, Hayonim Cave, W. Galilee, Israel
Source: PLoS One. 2024 Jul 24;19(7):e0301102. doi: 10.1371/journal.pone.0301102 (PMC11268594; doi:10.1371/journal.pone.0301102)
Supplement: S2 File — (DOCX) [file pone.0301102.s002.docx]

**S2) Type List**

| **A** | **End-scraper** |
| --- | --- |
| A01 | Single end-scraper |
| A02 | Atypical end-scraper |
| A03 | Double flat end-scraper |
| A04 | Alternate flat end-scraper |
| A05 | Ogival end-scraper |
| A06 | End-scraper on a retouched blade |
| A07 | End-scraper on an Aurignacian blade |
| A08 | Fan-shaped end-scraper |
| A09 | Flake scraper |
| A10 | End-scraper on a Levallois point |
| A11 | Circular (end) scraper |
| A12 | Thumb-nail end-scraper |
| A13 | Carinated end-scraper |
| A14 | Atypical carinated end-scraper |
| A15 | Lateral carinated end-scraper |
| A16 | Thick nosed end-scraper |
| A17 | Flat nosed or shouldered end-scraper |
| A18 | Micro-carinated end-scraper |
| A19 | Multiple carinated end-scraper |
| A20 | Rabot or thick end-scraper on a core |
| A21 | Massive Endscraper |
| A22 | Broken Endscraper |
| A23 | Double mixed endscraper |
| A93 | Inverse Endscraper |
| A94 | Other endscraper |
| A95 | Thick shouldered scraper |
| A96 | Endscraper on a notch |
| A97 | Single Endscraper, lateral |
| A98 | Bilateral endscraper |
| A99 | Unilateral endscraper |
| **B** | **Burins** |
| B01 | Straight dihedral burin |
| B02 | Offset dihedral burin |
| B03 | Angle dihedral burin |
| B04 | Dihedral burin on a break |
| B05 | Multiple dihedral burin |
| B06 | Beaked burin |
| B07 | Carinated burin |
| B08 | Flat-faced carinated burin |
| B09 | Right angle straight truncation burin |
| B10 | Burin on a straight oblique truncation |
| B11 | Burin on a concave truncation |
| B12 | Burin on a convex truncation |
| B13 | Oblique burin on lateral preparation |
| B14 | Transverse burin on lateral preparation |
| B15 | Transverse burin on a notch |
| B16 | Multiple burin on truncation |
| B17 | Mixed multiple burin |
| B18 | Flat burin |
| B86 | Burin on multiple truncation |
| B87 | Burin on previous facet |
| B88 | varia Burin |
| B89 | Burin, multiple, dihedral, multifaceted |
| B90 | Burin on concave truncation multifaceted |
| B91 | Burin on oblique truncation, multifaceted |
| B92 | Burin on straight truncation, multifaceted |
| B93 | Burin on snap, multifaceted |
| B94 | Burin on old surface, multifaceted |
| B95 | Burin, dihedral, angle, multifaceted |
| B96 | Burin dihedral multifaceted |
| B97 | Burin on natural surface |
| B98 | Corbiac Burin |
| B99 | Transverse Burin on lateral truncation |
| **C** | **Borers** |
| C01 | Spike |
| C02 | Beak |
| C03 | Borer |
| C04 | Heavy borer |
| C05 | Curved borer |
| C06 | Micro borer |
| C07 | Multiple borer |
| C08 | Awl |
| **D** | **Knives and backed pieces** |
| D01 | Naturally-backed piece |
| D02 | Atypical backed knife |
| D03 | Knife with curved back |
| D04 | Knife with straight back |
| D05 | Piece with irregular back |
| D06 | Piece with two backed edge |
| D07 | Partially backed piece |
| D08 | Backed point (cf Falita point) |
| D09 | Backed fragment |
| D10 | Shouldered piece |
| D12 | pointed piece |
| D99 | backed piece |
| **E** | **Truncations** |
| E01 | Truncated flake |
| E02 | Truncated blade |
| E03 | piece with curved truncation |
| E04 | Bitruncated piece |
| E05 | Backed and truncated piece |
| E98 | Straight truncation |
| E99 | Oblique Truncation |
| **F** | **Notches and denticulates** |
| F01 | Clactonian notch |
| F02 | Retouched notch |
| F03 | Blade or bladelet with multiple notches |
| F04 | Denticulate |
| F05 | Alternate burinant beak |
| F06 | Denticulate scraper |
| F99 | Notched piece |
| **G** | **Composites** |
| G01 | Flat end-scraper/burin |
| G02 | Carinated end-scraper/burin |
| G03 | Endscraper-truncated piece |
| G04 | Burin-truncated piece |
| G05 | Borer-truncated piece |
| G06 | Borer/end-scraper |
| G07 | Borer-burin |
| G85 | Notch/Denticulate |
| G86 | Scraper/Notch |
| G87 | Scraper/Denticulate |
| G88 | Other combined tools |
| G89 | Burin/Notched piece |
| G90 | Aurignacian blade/multiple burin |
| G91 | carinated burin/splintered piece |
| G92 | Carinated burin/ Notched piece |
| G93 | Blade with Aurignacian retouch/ carinated burin |
| G94 | Nosed/shouldered endscraper/burin |
| G95 | Splintered piece/endscraper |
| G96 | Truncation/Blade with Aurignacian retouch |
| G97 | carinated burin/endscraper |
| G98 | carinated burin/carinated endscraper |
| G99 | Burin/ret. Blade |
| **H** | **Retouched pieces** |
| H01 | Flake with continuous retouch |
| H02 | Blade with continuous retouch on one edge |
| H03 | Blade with continuous retouch on both edge |
| H04 | Aurignacian blade |
| H05 | Strangled blade or blade with a wide notch |
| H06 | Piece with inverse or alternate retouch |
| H07 | Pointed piece |
| H74 | retouched piece |
| H75 | Helwan blade |
| H76 | Fragment of backed blade |
| H77 | Blade with two backed edges |
| H78 | Blade with irregular backing |
| H79 | partially backed blade |
| H80 | backed blade |
| H81 | Fragment of retouched blade |
| H82 | partially retouched flake |
| H83 | partially retouched blade |
| H84 | Finely retouched blade |
| H85 | Flake bilaterally retouched |
| H86 | piece with Aurignacian retouch |
| H87 | retouched piece, unspecified |
| H88 | retouched flake, inverse |
| H89 | retouched flake, obverse |
| H90 | retouched flake, alternate |
| H91 | retouched flake, transverse |
| H92 | retouched blade, transverse |
| H93 | retouched blade, bilateral |
| H94 | retouched blade, inverse |
| H95 | retouched blade, obverse |
| H96 | retouched blade, alternate |
| H98 | retouched flake |
| H99 | retouched blade |
| **I** | **Special tools** |
| I01 | El Wad point |
| I02 | Splintered piece |
| I03 | Raclette |
| I04 | Denticulated scraper (Ksar Akil scraper) |
| I05 | Divers |
| I06 | Side scraper |
| I07 | Chamfered piece |
| I92 | Emireh Point |
| I93 | Pointe à face plane |
| I94 | Massive burin |
| I95 | massive battered piece |
| I96 | pick/chopping tool |
| I97 | Chisel/retoucher |
| I98 | Massive scraper |
| I99 | heavy duty denticulate |
| **J** | **Non-geometric microlith** |
| J01 | Pointed bladelet with fine retouch |
| J02 | Blunt bladelet with fine retouch |
| J03 | Bladelet with back curved by fine retouch |
| J04 | Fragment of bladelet with fine retouch |
| J05 | Pointed bladelet with abrupt retouch |
| J06 | Blunt bladelet with abrupt retouch |
| J07 | Bladelet with back curved by abrupt retouch |
| J08 | Short bladelet with back curved by abrupt retouch |
| J09 | Fragment of bladelet with abrupt retouch |
| J10 | Bladelet with inverse retouch |
| J11 | Bladelet with alternate retouch |
| J12 | Microgravette |
| J13 | Bladelet with Helwan retouch |
| J14 | Bladelet with Barajné retouch |
| J15 | Truncated bladelet |
| J16 | Backed and truncated bladelet |
| J17 | Backed bladelet with alternate truncation |
| J18 | Backed bladelet with a truncation forming an acute angle or a point |
| J68 | obliquely truncated |
| J69 | partially backed bladelet |
| J70 | retouched and truncated bladelet |
| J71 | retouched/backed bladelet fragments |
| J72 | Double truncated Helwan bladelet |
| J73 | Helwan Point |
| J74 | Atypical Ramon Point |
| J75 | Ramon Point with basal modifications |
| J76 | Ramon Point |
| J77 | La Mouillah Point with basal modifications |
| J78 | La Mouillah Point |
| J79 | Arched backed bladelet with basal modifications |
| J80 | Scalene Bladelet with basal modifications |
| J81 | Scalene bladelet |
| J82 | Obliquely truncated and backed bladelet |
| J83 | Obliquely truncated bladelet (Jiita point, Kebara Point) |
| J84 | Micropoint with basal modifications |
| J85 | Micropoint |
| J86 | pointed backed bladelet with basal modifications |
| J87 | Falita Point |
| J88 | Varia |
| J89 | Lamelle Dufour |
| J90 | backed bladelet |
| J91 | Partially retouched bladelet |
| J92 | Bladelet with Ouchtata Retouch |
| J93 | Bladelet with bilateral retouch |
| J94 | Micro awl |
| J95 | retouched bladelet |
| J96 | retouched bladelet, obverse |
| J97 | partially backed bladelet |
| J98 | Dufour bladelet, inverse |
| J99 | Dufour bladelet, alternate |
| **K** | **Geometric microlith** |
| K01 | Bitruncated bladelet |
| K02 | Narrow backed and bitruncated bladelet |
| K03 | Trapeze-rectangle |
| K04 | Trapeze-rectangle with an end broken |
| K05 | Truncated triangle |
| K06 | Trapeze-rectangle with Helwan retouch |
| K07 | Short abruptly-retouched isoceles or scalene triangle |
| K08 | Elongated scalene triangle with abrupt retouch |
| K09 | Triangle with Helwan retouch |
| K10 | Triangle with Barajné retouch |
| K11 | Lunate with abrupt retouch |
| K12 | Atypical lunate |
| K13 | Lunate with Helwan retouch |
| K80 | Parallelogram |
| K81 | unbacked trapeze |
| K82 | Proto-Rectangle |
| K83 | Abu Maadi Point |
| K84 | Khiam Point |
| K85 | Ounan point |
| K86 | Harif Point |
| K87 | Proto-harif Point |
| K88 | Shunera Point |
| K89 | Atypical triangle |
| K90 | Isoceles triangle |
| K91 | Lunate |
| K92 | Atypical Helwan Lunate |
| K93 | Trapeze with one convex end |
| K94 | Asymmetrical trapeze B |
| K95 | Asymmetrical trapeze A |
| K96 | Trapeze |
| K97 | Proto-trapeze |
| K98 | Rectangle |
| K99 | Straight truncated and backed |
| **L** | **Microburin technique** |
| L01 | Microburin |
| L02 | Krukowski microburin |
| L03 | "Piquant trièdre" |
| L04 | Backed bladelet with " piquant trièdre |
| L99 | Microburin with "piquant trièdre" |
| **M** | **Divers** |
| M01 | Shouldered bladelet |
| M02 | Sickle-blade element |
| M03 | Arrowhead |
| M98 | Limestone disc |
| M99 | Hammerstone |
