# Supplementary material for: The use of carinated items in the Levantine Aurignacian—Insights from layer D, Hayonim Cave, W. Galilee, Israel
Source: PLoS One. 2024 Jul 24;19(7):e0301102. doi: 10.1371/journal.pone.0301102 (PMC11268594; doi:10.1371/journal.pone.0301102)
Supplement: S3 File — (DOCX) [file pone.0301102.s003.docx]

**S3) Attributes recorded in the analysis**

Blanks

Site
Square
Quarter square
Find-Number
Level
Elevation
x
y
z
Datum
Blank as recorded single plotted
L(ength)
W(idth)
Th(ickness)
w/g (weight)
Blank
 1) Blade
 2) Bladelet
 3) Flake
 4) Core
 5) Chunk artificial
 6) Chunk natural
 7) Chip
 8) indeterminable fragment
 9) raw nodule/manuport

Blank fragmented? Yes/No
Tool? Yes/No
CTE? Yes/No
Clear Carinated product? Yes/No
Carinated item? Yes/No
Carinated preform? Yes/No
CO-Phase
 0) indeterminable
 1) I – Raw material acquisition
 2) II – Initial Opening
 3) III – Decortification
 4) IV – Preparation
 5) V – Reduction
 6) VI – Maintenance
 7) VII – Discard
 8) VIII – Use
CO-Product type
 0) indeterminable
 1) raw nodule
 2) fully cortical opening
 3) natural crest
 4) unilateral primary crest
 5) bilateral primary crest
 6) unilateral secondary crest
 7) bilateral secondary crest
 8) lateral core trimming
 9) reduction target
 10) reduction by-product
 11) core tablet primary
 12) core tablet secondary
 13) primary neocresting
 14) secondary neocresting
 15) tool
 16) core
 17) lateral cortical bladelet
 18) Initial burin spall
Preparation/Maintenance typology, see text and Fig. 5
Cortex
 1) none
 2) lateral
 3) distal
 4) proximal
 5) complete
 6) lateral and distal
 7) lateral and proximal
 8) medial
 9) distal and proximal
 10) lateral, distal and proximal
 11) lateral and medial
 12) bilateral
 13) back
 14) back and lateral
Blank preservation
 1) complete
 2) distal
 3) proximal
 4) medial
 5) proximal and medial
 6) distal and medial
Platform
 1) plain
 2) primary facetted
 3) secondary facetted
 4) linear/ridge
 5) punctiform
 6) dihedral
 7) splintered
 8) indeterminable
 9) not present
Bulb
 1) developed
 2) diffuse
 3) Hertzian cone
 4) double
 5) ridge
 6) not present
 7) proximal end not preserved

Scar
 1) proximal end not preserved
 2) transverse
 3) aligned
 4) not present
Lip
 1) proximal end not preserved
 2) present
 3) not present
Abrasion Yes/No
Knapping angle
 1) acute
 2) blunt
 3) 90°
 4) indeterminable
 5) proximal end not preserved
Longitudinal section
 1) straight
 2) curved smoothly
 3) curved proximally
 4) curved medially
 5) curved distally
 6) indeterminable
Cross section
 1) equilateral triangular
 2) flat triangular lateralised
 3) flat triangular central
 4) trapezoidal central
 5) trapezoidal lateralised
 6) rectangular
Twisting
 1) right
 2) left
 3) straight
 4) interminable
Ventral remains? Yes/No
Location ventral remains
 1) right
 2) left
 3) not present
 4) indeterminable
Direction dorsal negatives
 1) aligned/same direction
 2) perpendicular
 3) mixed
 4) aligned and cross
 5) perpendicular and cross
 6) mixed and cross
 7) converging
 8) oblique
 9) unidirectional cross
 10) bidirectional cross
 11) other
 12) indeterminable
 13) no dorsal negatives/cortex
 14) converging and aligned
Distal preparation
 1) initial unilateral continuous
 2) initial unilateral distal primary
 3) initial unilateral distal secondary
 4) initial bilateral continuous
 5) initial bilateral distal primary
 6) initial bilateral distal secondary
 7) neocrest unilateral continuous
 8) neocrest unilateral distal primary
 9) neocrest unilateral distal secondary
 10) neocrest bilateral continuous
 11) neocrest bilateral distal primary
 12) neocrest bilateral distal secondary
 13) not present
 14) indeterminable
 15) neocrest unilateral continuous secondary
 16) neocrest bilateral continuous secondary
Proximal preparation/core table Yes/No
Fracture termination
 1) not present
 2) flat
 3) hinge
 4) overshot
 5) thick
 6) indeterminable
Tool type cf. Type list S2)

Carinated Cores

Site
Square
Find No
# Reduction surfaces (each one separately recorded in the case of multiple carinated elements)
Blank type see blanks above
Core type
 1) carinated burin (CB)
 2) preform
 3) bladelet core
 4) multiple CB
 5) maybe CB
 6) flat faced CB
 7) Vachons
 8) beaked CB
 9) carinated endscraper (CE)
 10) nosed enscraper
 11) maybe CE
 12) blade core
 13) flake core
 14) mixed core
 15) amorphous
 16) bidirectional opposite
 17) Levallois-like
 18) 90° angle
Cortex see blanks
n Reduction surfaces
Core preparation
 1) platform preparation
 2) distal preparation
 3) back preparation
 4) platform and distal
 5) platform and back
 6) distal and back
 7) platform, distal and back
 8) not present
 9) lateral
 10) lateral and back
 11) lateral and platform
 12) lateral, back and platform
Platform preparation
 1) negatives from ventral
 2) negatives from dorsal
 3) negatives bilateral
 4) tablet/burin spall
 5) tablet and negatives
 6) multiple tablet
 7) multiple tablet and negatives
 8) natural/cortical
 9) none
 10) facetted (from frontal)
Reduction surface lateralisation
 1) straight
 2) ventral right
 3) ventral left
 4) dorsal left
 5) dorsal right
 6) indeterminable
 7) CE left
 8) CE right
Shape reduction surface
 1) twisted
 2) converging
 3) subparallel
 4) other
n core tablets
Length reduction surface
Width reduction surface
Abrasion last negative
 1) indeterminable
 2) present
 3) absent
Stopping retouch? Yes/No
Fracture termination last negative
 1) hinge
 2) flat
 3) overshot
 4) indeterminable
Comment: Why discarded?

Analysis non-carinated cores

Site
Square
Sub square
Find No
Level
Elevation
Burned? Yes/No
Heat treated? Yes/No
Cortex (see above)
Nodule type
 0) indeterminable
 1) full nodule
 2) tabular section
 3) irregular chunk
 4) flake
 5) biface
 6) intrusion
Core shape
 0) indeterminable
 1) pyramidal
 2) parallel
 3) amorphous
 4) discoidal
 5) prismatic
 6) core on flake
 7) centripetal
 8) tested
 9) endscraper-like
 10) burin-like
 11) narrow-fronted
Shape reduction surface
 1) twisted
 2) converging
 3) subparallel
 4) other
n reduction surfaces
n platforms
broken? Yes/No
twisted? Yes/No
Natufian? Yes/No
Comment
